# A biosensor toolbox for monitoring recombinant protein production in *Escherichia coli*

**DOI:** 10.3389/fbioe.2026.1956994

**Published:** 2026-10-07

**Authors:** Maurice Mürköster, Mateusz Balka, Christina J. Kamp, Judit Källström, Linus Karlsson, Claudia A. Rodriguez Aparicio, Erik Tunäng, Hanna-Louise Ögren, Silvia Theopold, Alister J. Cumming, Alex Toftgaard Nielsen, Daniel O. Daley

**Affiliations:** 1 Department of Biochemistry and Biophysics, Stockholm University, Stockholm, Sweden; 2 The Novo Nordisk Foundation Biotechnology Research Institute for the Green Transition (BRIGHT), Technical University of Denmark, Lyngby, Denmark

**Keywords:** biosensor, Cpx stress response, heat shock response, recombinant protein production, SecM secretion monitor

## Abstract

Recombinant protein production can be a challenging process. To help users troubleshoot and optimize experiments in *Escherichia coli* we have compiled and characterized a toolbox of biosensor plasmids. It contains a biosensor that monitors the translational efficiency of the recombinant protein and quality control biosensors that detect unwanted events, such as protein misfolding in the cytoplasm, inefficient translocation through the Sec translocon, or protein misfolding in the periplasm. The biosensors were combined with different fluorescent proteins so that multiple events could be measured in parallel, in the same cell. The toolbox is a resource for diagnosing protein production problems and evaluating solutions that mitigate these problems.

## Introduction

*Escherichia coli* is widely used for the production of recombinant proteins. These proteins may be purified for use as biopharmaceuticals, vaccines, diagnostic tools, biotechnology reagents, foods and food additives, or research objects (Walsh, 2018; Gavrilescu and Chisti, 2005; McElwain et al., 2022; De Pinho Favaro et al., 2022; Choi et al., 2024; Graham and Ledesma-Amaro, 2023). Alternatively, they may be used within the cell to drive biosynthetic or metabolic pathways for the production of high-value chemicals (Lee et al., 2019) or biofuels (Wang et al., 2017). Across these applications, it is critically important that recombinant proteins are localized to the correct compartment, folded and functional.

The Protein Data Bank contains structural information for more than 128,000 proteins produced recombinantly in *E. coli* (Heyde and Nørholm, 2021), highlighting its broad utility for recombinant protein production. However, a deeper analysis of data from the platforms that produced these recombinant proteins paints a different picture. Only approximately 50% of bacterial targets and 10% of eukaryotic targets could be produced in sufficient quality and titers (Structural Genomi cs Consortium et al., 2008; Vincentelli et al., 2003; Braun and LaBaer, 2003). A range of unwanted events can limit recombinant protein production in *E. coli*, including ribosome stalling, protein misfolding, protein aggregation, and inefficient translocation to the periplasm (Vazulka et al., 2024; Wang et al., 2013; Frenzel et al., 2013; Pirkalkhoran et al., 2023). These events can activate cellular stress responses, which trigger transcriptional reprogramming and divert resources away from growth and biomass accumulation (Vazulka et al., 2024; Wild et al., 1993; Jones et al., 2003). Stress responses can, in turn, interfere with protein synthesis and plasmid stability (Snoeck et al., 2024; Cumming et al., 2024), potentially resulting in reduced production titers and loss of production capacity during prolonged cultivation. In severe cases, stress responses can lead to cell death (Hunke and Betton, 2003). Consequently, identifying and mitigating the aforementioned unwanted events is critical for efficient recombinant protein production.

Several strategies can be used to mitigate unwanted events and the stress they cause. One relatively simple approach is to optimize induction conditions, such as inducer concentration, temperature, and growth rate, to ensure that the rate of recombinant protein synthesis does not exceed the cellular capacity for protein folding and quality control (Karyolaimos et al., 2019; Horga et al., 2018). Alternatively, the recombinant DNA sequence can be engineered to improve protein folding or promote efficient translocation to the periplasm (Low et al., 2013; Zutz et al., 2021). The host cell can also be engineered by supplementing key chaperones (Wang et al., 2013; De Marco et al., 2007), or removing proteases that contribute to recombinant protein degradation (Rosano and Ceccarelli, 2014). Evaluating the effectiveness of these strategies typically requires extensive screening of protein titers and quality to determine if they have been successful. Traditionally this has been done using biochemical approaches that report on the steady-state levels of the proteins, such as SDS-PAGE of whole cells or fractionated samples followed by Coomassie staining or Western blotting. Although these approaches are informative, they are laborious and time-consuming and not compatible with single cell screening of sequence libraries. There is a need for approaches that enable rapid, quantitative, and scalable monitoring of recombinant protein production and the cellular stresses associated with it.

In recent years biosensor plasmids have been developed that allow users to monitor recombinant protein production in *E. coli*. For example, plasmids that contain a translation coupling device and the coding sequence for a reporter protein can be used to quantify the translational efficiency of a recombinant protein (Zutz et al., 2021; Mendez-Perez et al., 2012). Similarly, plasmids containing stress-activated promoters fused to reporter genes can be used to detect cellular stress caused by protein misfolding. Misfolded proteins in the cytoplasm activate the heat shock response (Yura, 2019), whereas misfolded proteins in the periplasm activate the Cpx extracellular stress response (Hunke and Betton, 2003). Activation of these stress responses results in the transcriptional upregulation of specific stress-responsive promoters in the production host. Biosensor plasmids are designed to exploit this phenomenon by fusing a promoter and 5′ UnTranslated Region (5′UTR) for a stress induced gene to the coding sequence for a reporter protein (i.e., luciferase, β-galactosidase, GFP, mCherry) (Cumming et al., 2024; Zutz et al., 2021; Andersen et al., 1998; Christensen et al., 2023; Kraft et al., 2007a; Kraft et al., 2007b; Götzke et al., 2014; Keller et al., 2015).

Although a number of biosensor plasmids have been constructed and characterized, they have been described across different publications and lack a standardized design. In particular, these plasmids differ in key characteristics, including plasmid copy number, antibiotic resistance marker, and reporter protein. This lack of standardization can make it difficult to directly compare biosensor performance and select an appropriate system for monitoring recombinant protein production. In this study we have addressed this problem, by assembling a toolbox of standardized biosensor plasmids for monitoring recombinant protein production in *E. coli*. The toolbox comprises two expression plasmids containing a translational biosensor, as well as a set of quality control biosensors that enable the monitoring of multiple unwanted events within the same cell. These include, protein misfolding in the cytoplasm, protein misfolding in periplasm, and inefficient co-translational translocation through the Sec translocon. To construct this toolbox, we first improved the sensitivity of an existing translational biosensor by replacing the mCherry coding sequence with mScarlet-I3. We also developed a new biosensor for monitoring the efficiency of translocation through the Sec translocon and evaluated previously described biosensors for protein misfolding in the cytoplasm.

## Results

### A biosensor for detecting translation efficiency

The toolbox contains a pBAD expression plasmid with a biosensor for monitoring the translational efficiency of a recombinant protein in live *E. coli* cells. The biosensor was constructed using a Translational Coupling Device (TCD) and the coding sequence of a red fluorescent protein (either mCherry or mScarlet-I3) (Zutz et al., 2021) (Figure 1A). The TCD is downstream of the coding sequence of interest and was designed so that the region encoding the C-terminal poly-His tag on the coding sequence would be sequestered into an mRNA hairpin with the Shine-Dalgarno (SD) sequence and AUG start codon of the downstream coding sequence for the red fluorescent protein (Mendez-Perez et al., 2012). This hairpin blocks translation of the red fluorescent protein as the ribosome cannot access the SD sequence. However, when the upstream coding sequence of interest is translated, the TCD is temporarily unfolded by the ribosome and the SD sequence for the red fluorescent protein becomes accessible. The [TCD-mCherry] biosensor has been characterized previously (Zutz et al., 2021), whereas the [TCD-mScarlet-I3] biosensor was constructed in this study. We observed that [TCD-mScarlet-I3] was more sensitive for detecting protein translation when we titrated the expression of a well-expressing and a poorly-expressing protein (Δss-MalE and Mth1 respectively; Figure 2). This can most likely be attributed to the fact that mScarlet-I3 is brighter and matures more rapidly than mCherry (see (Gadella et al., 2023) and Supplementary Material, Supplementary Figure S1). We did not observe significant differences in the OD_600_, or the performance of the [P_ibpA/fxsA_-gfp] misfolding biosensor (described below) when [TCD-mCherry] and [TCD-mScarlet-I3] were compared.

**FIGURE 1:**
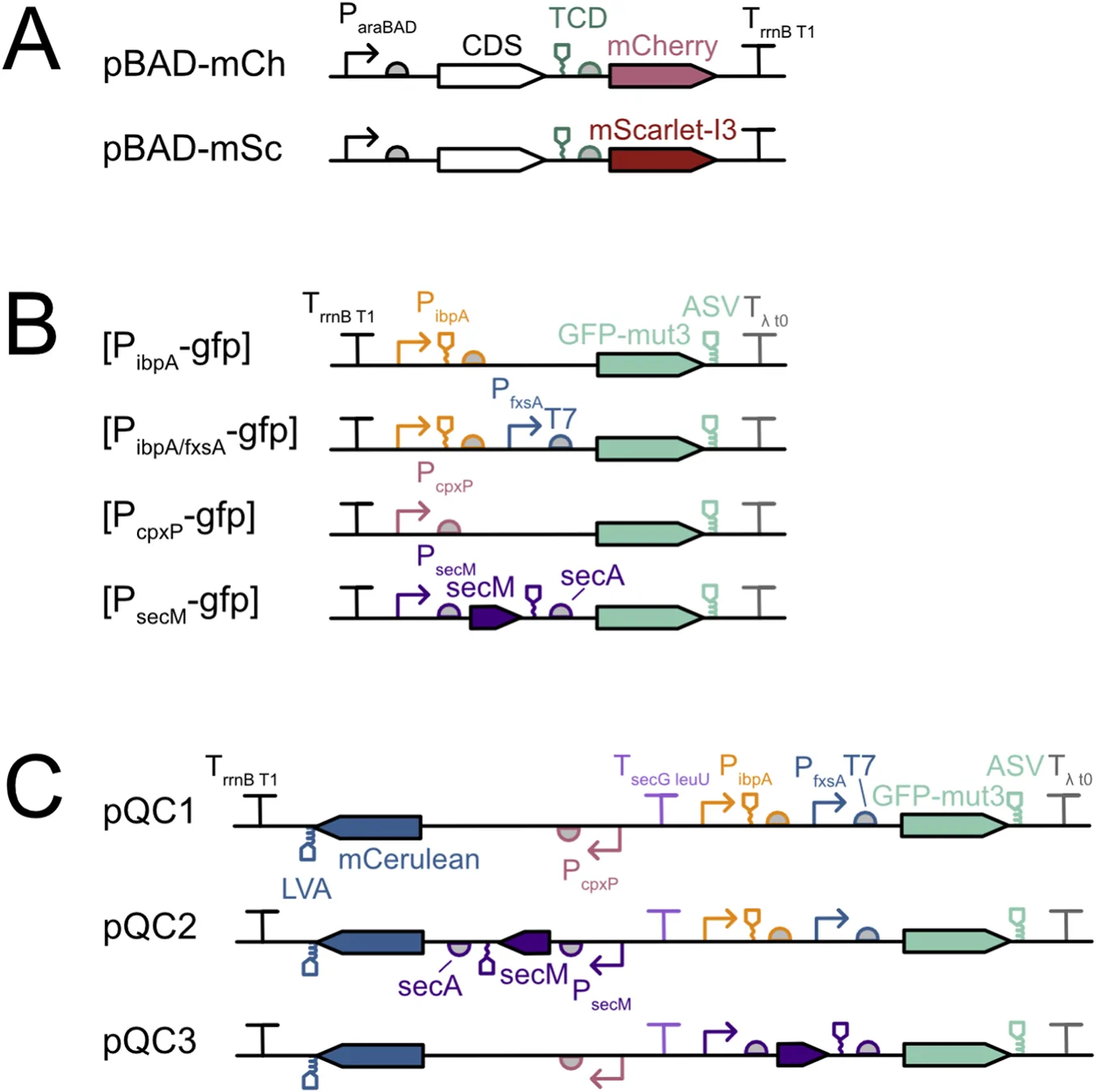
Cartoon representations of the biosensor toolbox. **(A)** Coding sequences being expressed were cloned into a pBAD expression plasmid, upstream of a Translational Coupling Device (TCD) and the coding sequence of either mCherry or mScarlet-I3. The TCD encodes a poly-His purification and detection tag. **(B)** Biosensors containing single modules were harbored in the pSEVA631(sp) backbone. [P_ibpA_-gfp] and [P_ibpA/fxsA_-gfp] can be used to detect the misfolding of proteins in the cytoplasm. [P_cpxP_-gfp] can be used to detect the misfolding of proteins in the periplasm. [P_secM_-gfp] can be used to detect inefficient translocation of proteins through the Sec translocon. **(C)** Biosensors containing two modules were also harbored in the pSEVA631(sp) backbone. pQC1 contains the [P_ibpA/fxsA_-gfp] and [P_cpxP_-mCerulean] modules. pQC2 contains the [P_ibpA/fxsA_-gfp] and [P_secM_-mCerulean] modules. pQC3 contains the [P_secM_-gfp] and [P_cpxP_-mCerulean] modules. All cartoons were drawn according to the Synthetic Biology Open Language (SBOL) visual standard (https://sbolstandard.org/visual/). The nucleotide sequences are available in Supplementary Material, Supplementary Table S2. The [TCD-mCherry] module in pBAD-mCh, [P_ibpA_-gfp] and [P_cpxP_-gfp] have been described previously (Cumming et al., 2024; Zutz et al., 2021). All other biosensors were constructed in this study.

**FIGURE 2:**
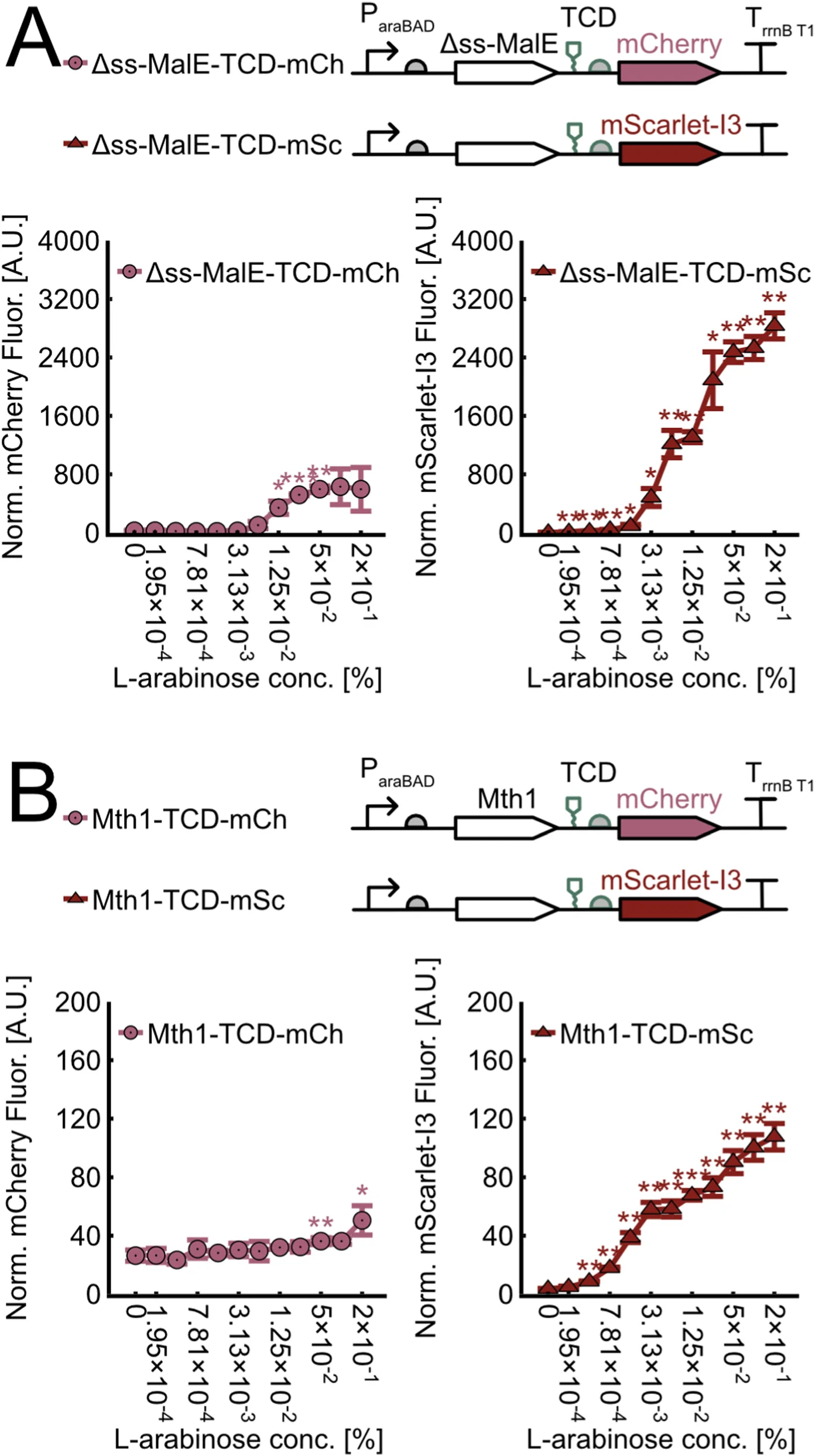
A more sensitive biosensor module for monitoring translational efficiency. Cartoons of plasmids used in the experiments are shown above each panel. **(A)** A comparison of the [TCD-mCherry] and [TCD-mScarlet-I3] expression cassettes was carried out by titrating Δss-MalE. The fluorescence output from the translationally coupled mCherry and mScarlet-I3 was normalized by the OD_600_. OD_600_ values are presented in Supplementary Material, Supplementary Table S1. All data presented as mean ± standard deviation (s.d.) (n ≥ 3). A statistically significant difference between the uninduced sample (0% (w/v) L-arabinose) and the induced sample is marked. P < 0.05 is denoted by *, P < 0.01 by **, and P < 0.001 by *** (two-tailed Student’s t-test). Data where no statistical difference was observed is not marked. **(B)** as per panel **(A)** except that Mth1 was expressed.

### Biosensors for detecting protein misfolding

When recombinant proteins misfold in the cytoplasm of *E. coli* (Rong et al., 2023; Baneyx and Mujacic, 2004) the cell activates the heat shock response that releases the Sigma 32 (σ^32^) transcription factor (Yura, 2019). The toolbox contains two biosensors that detect when the heat shock response has been activated (Zutz et al., 2021). [P_ibpA_-gfp] contains the σ^32^-dependent promoter and 5′UTR for the gene encoding the inclusion body associated binding protein A (*ibpA*) fused to the coding sequence of a fast-folding and fast-degrading version of the Green Fluorescent Protein (GFP-mut3_ASV_; maturation time (*t*_*1/2*_) = 4.1 min (Balleza et al., 2018) and degradation time (*t*_*1/2*_) = 110 min (Andersen et al., 1998)). [P_ibpA/fxsA_-gfp] contains the σ^32^-dependent promoter and the 5′UTR for the *fxsA* gene to the 3′ end of the promoter and 5′UTR of *ibpA* fused to the coding sequence of GFP-mut3_ASV_. Both biosensors are harbored in a low-copy backbone constructed using the Standard European Vector Architecture (pSEVA631(sp)) (Martínez-García et al., 2023) (Figure 1B). These biosensors have been characterized previously (Zutz et al., 2021; Kraft et al., 2007a; Englund et al., 2023). When we compared them in control experiments with a cytoplasmic protein that folds (Δss-MalE) and another that misfolds (*M*th1), we noted that they could distinguish the folded protein from the misfolded protein (Supplementary Material, Supplementary Figure S2). We also observed that the background and the maximum output from [P_ibpA/fxsA_-gfp] was higher than that from [P_ibpA_-gfp]. [P_ibpA/fxsA_-gfp] was therefore used in future work where we constructed dual-color biosensors (see below).

Recombinant proteins are sometimes engineered with an N-terminal signal peptide that traffics them to the Sec translocon, and facilitates their translocation to the periplasm of *E. coli* (De Geyte et al., 2016; Tsirigotaki et al., 2017). Recombinant proteins that misfold in the periplasm activate the Cpx extracellular stress response (Hunke and Betton, 2003). This results in the phosphorylation of the CpxR transcription factor, which guides the RNA polymerase (RNAP) to designated promoters in the genome of the production host (Price and Raivio, 2009; Raivio, 2014). In the toolbox there is a biosensor that detects when the Cpx stress response is activated. [P_cpxP_-gfp] contains the CpxR-dependent promoter and 5′UTR for the gene encoding the Cpx regulator protein (*cpxP*) fused to the coding sequence of GFP-mut3_ASV_ (Figure 1B). It was constructed and characterized previously (Cumming et al., 2024). In this study we screened additional Cpx-based biosensors but we were unable to find one that performed better than [P_cpxP_-gfp] in control experiments (Supplementary Figures S3 and S4).

### A biosensor for detecting secretion problems

The toolbox contains a biosensor that monitors the efficiency of co-translational secretion through the Sec translocon. This biosensor was designed to mimic the SecM-SecA regulatory system in *E. coli* (Nakatogawa et al., 2004; Ito et al., 2018). SecM is a periplasmic protein that is co-translationally targeted to the Sec translocon, where it is translocated to the periplasm and subsequently degraded. When the capacity of the Sec translocon is exceeded, SecM cannot be co-translationally targeted to the Sec translocon and it becomes stalled on the ribosome. The stalling disrupts an mRNA hairpin downstream of the *secM* coding sequence which enables translation of the downstream *secA* coding sequence. SecA is then able to alleviate the translocation bottleneck at the translocon. The [P_secM_-gfp] biosensor we engineered contains the promoter, the 5′UTR and the coding sequence for *secM* fused to the coding sequence for GFP-mut3_ASV_ (Figure 1B). It was designed to produce GFP when a recombinant periplasmic protein prevents SecM from being efficiently targeted to the Sec translocon.

We evaluated the performance of [P_secM_-gfp] by expressing the maltose binding protein (MalE), and a single chain antibody fragment (scFv). These proteins were engineered with the signal peptide from MalE (pre^MalE^) and should therefore be post-translationally targeted to the Sec translocon (Bassford, 1990). We also engineered MalE and scFv with the signal peptide from DsbA (pre^DsbA^-) so they should be co-translationally targeted to the Sec translocon (Schierle et al., 2003). All four coding sequences were cloned into a pBAD plasmid upstream of the [TCD-mCherry] biosensor and expressed in MC1061 (which cannot metabolize L-arabinose) by induction with increasing concentrations of L-arabinose. We reasoned that at high induction concentrations the capacity of the Sec translocon would be exceeded. Fluorescence from the translationally-coupled mCherry indicated that pre^MalE^-scFv was not efficiently translated but that all others were (Figures 3A,B). [P_secM_-gfp] was activated when the co-translationally targeted pre^DsbA^-MalE and pre^DsbA^-scFv were induced, but not when the post-translationally targeted pre^MalE^-MalE and pre^MalE^-scFv were induced (Figures 3A,B). The final OD_600_ of the cells producing pre^MalE^-MalE and pre^MalE^-scFv did not give any indication that the proteins were toxic to the cells. However, the production of pre^DsbA^-MalE and pre^DsbA^-scFv did affect the final OD_600_ of the cells (Supplementary Table S1). SDS-PAGE analysis of whole cells indicated that the retained version (containing the signal peptide, denoted P) was visible when all four proteins when cells were induced with 2 × 10^−3^% (w/v) L-arabinose (Figures 3C,D). Notably, [P_secM_-gfp] was not activated when the signal peptide was omitted from MalE (Δss-MalE) and scFv (Δss-scFv) as the proteins were not targeted to the Sec translocon (Supplementary Figure S5). Taken together the data indicate that [P_secM_-gfp] can detect when fusions using the DsbA signal peptide (pre^DsbA^-) are retained in the cytoplasm, but not when fusions using the MalE signal peptide (pre^MalE^-) are retained. This observation suggests that the [P_secM_-gfp] biosensor can detect when a co-translationally targeted recombinant protein is inefficiently secreted, but not when a post-translationally targeted recombinant protein is inefficiently secreted. This could be because the SecM secretion sensor is itself co-translationally secreted, or because post-translationally targeted recombinant proteins are more efficiently secreted and do not block the SecM secretion sensor. Further work is required to fully understand the limitations of this biosensor.

**FIGURE 3:**
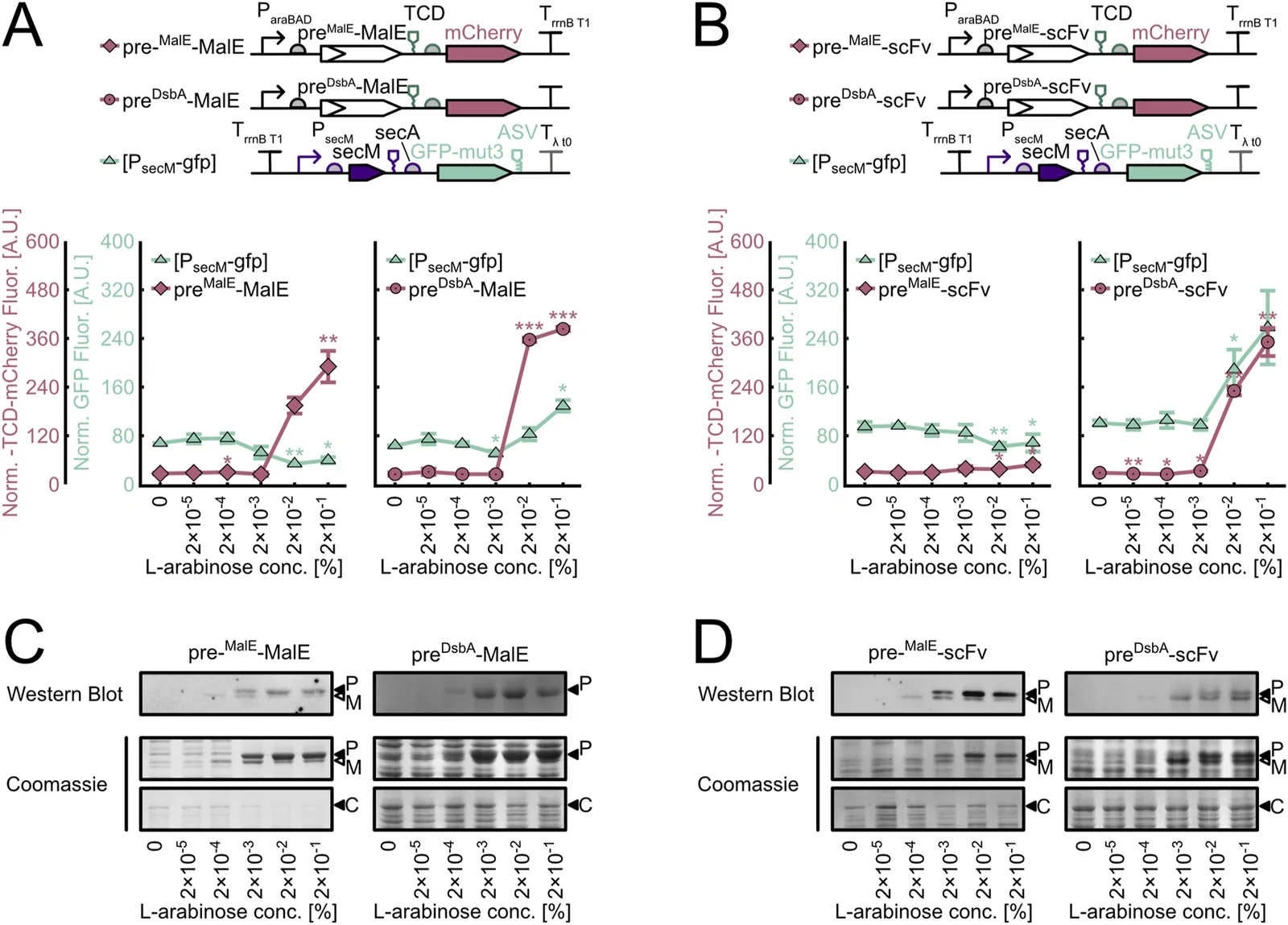
The efficiency of protein translocation to the periplasm was monitored with [P_secM_-gfp]. Cartoons of plasmids used in the experiments are shown above each panel. **(A)** pre^MalE^-MalE and pre^DsbA^-MalE were expressed with increasing concentrations of L-arabinose. The fluorescence output from the translationally coupled mCherry was normalized by the OD_600_ (red). The fluorescence output from [P_secM_-gfp] was normalized by the OD_600_ (green). All data presented as mean ± standard deviation (s.d.) (n ≥ 3). A statistically significant difference between the uninduced sample (0% (w/v) L-arabinose) and the induced sample is marked. P < 0.05 is denoted by *, P < 0.01 by **, and P < 0.001 by *** (two-tailed Student’s t-test). Data where no statistical difference was observed is not marked. **(B)** As per panel **(A)** except that pre^MalE^-scFv and pre^DsbA^-scFv were expressed. **(C)** The steady state levels of pre^MalE^-MalE and pre^DsbA^-MalE were monitored by separating proteins by SDS-PAGE and Western blotting. This experiment also indicated whether the signal peptide had been retained (indicative of a cytoplasmic location). As proteins enter the periplasm, the signal peptide is cleaved off (Paetzel, 2014), thus the presence/absence of the signal peptide can be used as a proxy for the location of the protein (Cumming et al., 2024). Signal peptide containing versions of each protein (localized to the cytoplasm) are denoted P, and the mature versions (localized to the periplasm) are denoted M. As all proteins contained a C-terminal poly-His tag they were detected with either the HIS Lite™ Cy3 Bis NTA-Ni Complex (proteins containing the pre^MalE^ signal peptide) or the HIS Lite™ Cy5 Bis NTA-Ni Complex (proteins containing the pre^DsbA^ signal peptide). A loading control on the Coomassie stained SDS-PAGE is denoted C. **(D)** As per panel **(C)** except that pre^MalE^-scFv and pre^DsbA^-scFv were expressed.

### Biosensors for detecting multiple events simultaneously

To monitor multiple unwanted events simultaneously we constructed plasmids containing two biosensor modules. In order to do so we first needed to identify a fluorescent protein that was spectrally distinct from GFP-mut3, yet behaved similarly with regards to folding times and turnover when fused to a degradation tag. We chose mCerulean as it fit all criteria when fused to an LVA degradation tag (Supplementary Figure S6). Moreover, it was also spectrally distinct from mCherry/mScarlet-I3, so it could be used in parallel with the translational biosensor.

We used GFP-mut3_ASV_ and mCerulean_LVA_ to engineer 3 Quality Control plasmids (pQCs) with multiple biosensors modules (Figure 1C). pQC1 contains the [P_ibpA/fxsA_-gfp]/[P_cpxP_-mCerulean] biosensor modules and is designed to monitor protein misfolding in the cytoplasm and periplasm. It is intended to replace a similar biosensor that we engineered previously called pQC, which is not compatible with the expression cassettes containing mCherry/mScarlet-I3 because it contains [P_cpxP_-mCherry] (Cumming et al., 2024). pQC2 contains the [P_ibpA/fxsA_-gfp]/[P_secM_-mCerulean] biosensor modules and is designed to monitor inefficient targeting to the Sec translocon by detecting aggregation in the cytoplasm and monitoring the Sec translocon. pQC3 contains the [P_secM_-gfp]/[P_cpxP_-mCerulean] biosensor modules and is designed to monitor the Sec translocon and protein aggregation in the periplasm.

pQC1, pQC2 and pQC3 were evaluated in control experiments with a periplasmic protein that folded (pre^MalE^-MalE) and another that misfolded (pre^MalE^-scFv) (Figure 4). In these experiments the coding sequence of interest was cloned into pBAD-mSc, upstream of the [TCD-mScarlet-I3] biosensor. The expression plasmid was then co-transformed into the MC1061 strain (which cannot metabolize L-arabinose) together with one of the pQC biosensor plasmids and recombinant proteins were expressed at 37 °C by induction with different concentrations of L-arabinose. Fluorescence in the red, green and blue spectrum was then captured from whole cells. When pre^MalE^-MalE was expressed, we observed increasing signal from the [TCD-mScarlet-I3] biosensor (Figures 4A,C,E). When pQC1 was used to monitor the quality of pre^MalE^-MalE, we observed that [P_ibpA/fxsA_-gfp] was activated (Figure 4A). This observation is consistent with previous work showing that at high expression levels pre^MalE^-MalE accumulates in the cytosol and activates the heat shock response (Cumming et al., 2024). We also observed that the [P_cpxP_-mCerulean] biosensor was not activated. This observation is also consistent with previous work showing that MalE is folded in the periplasm ((Cumming et al., 2024) and above). When pQC2 was used to monitor the quality of pre^MalE^-MalE, we again observed increasing signal from the [P_ibpA/fxsA_-gfp] biosensor (as observed with pQC1) (Figure 4C). We also observed that the [P_secM_-mCerulean] biosensor was not activated. This observation indicates that pre^MalE^-MalE is not disrupting traffic through the Sec translocon. When pQC3 was used to monitor the quality of pre^MalE^-MalE, we observed that the [P_secM_-gfp]/[P_cpxP_-mCerulean] biosensors performed similarly to those in pQC1 and pQC2 (Figure 4E). Taken together, these data from the pQC biosensor plasmids indicate that pre^MalE^-MalE is efficiently expressed, but at high expression levels it aggregates in the cytoplasm. It does not experience a problem at the Sec translocon, or a problem folding in the periplasm.

**FIGURE 4:**
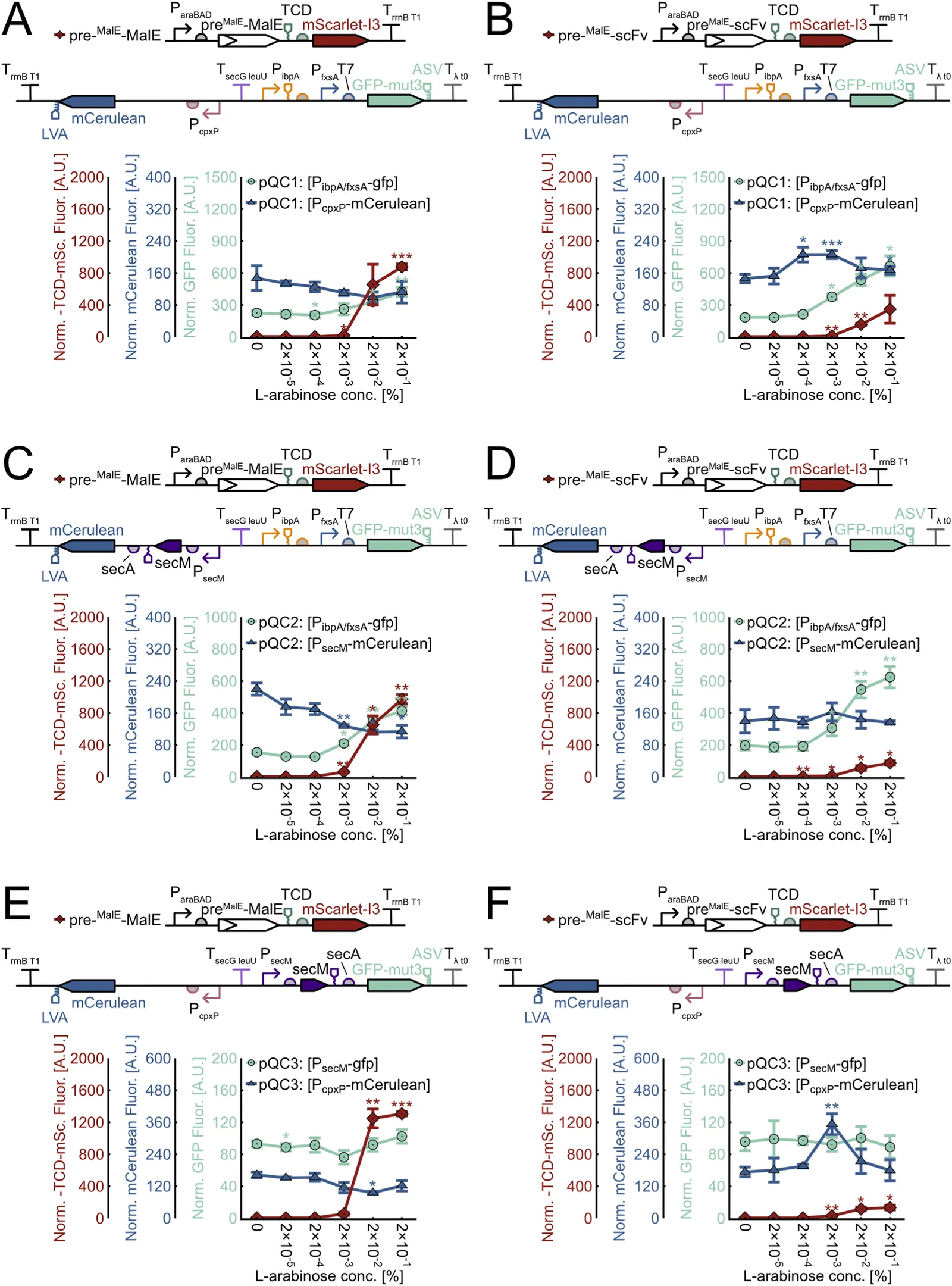
Biosensors that detect multiple events. Cartoons of plasmids used in the experiments are shown above each panel. **(A)** pre^MalE^-MalE was cloned upstream of the translationally coupled mScarlet-I3 and pQC1 was used to monitor its quality. In the experiment pre^MalE^-MalE was expressed with increasing concentrations of L-arabinose and the fluorescence output from the translationally coupled mScarlet-I3 was normalized by the OD_600_ (red). The fluorescence output from the biosensor modules in pQC1 was normalized by the OD_600_ (green and blue). OD_600_ values are presented in Supplementary Material, Supplementary Table S1. All data presented as mean ± standard deviation (s.d.) (n ≥ 3). A statistically significant difference between the uninduced sample (0% (w/v) L-arabinose) and the induced sample is marked. P < 0.05 is denoted by *, P < 0.01 by **, and P < 0.001 by *** (two-tailed Student’s t-test). Data where no statistical difference was observed is not marked. **(B)** As for **(A)**, except that pre^MalE^-scFv was expressed. **(C)** As for **(A)**, except that pQC2 was used. **(D)** As for **(B)**, except that pQC2 was used. **(E)** As for **(A)**, except that pQC3 was used. **(F)** As for **(B)**, except that pQC3 was used.

When pre^MalE^-scFv was expressed, we also observed increasing signal from the [TCD-mScarlet-I3] biosensor (Figures 4B,D,F). pQC1 indicated that the [P_ibpA/fxsA_-gfp] biosensor was activated, which is consistent with previous work showing that at high expression levels pre^MalE^-scFv accumulates in the cytosol and activates the heat shock response (Figure 4B). We also observed that the [P_cpxP_-mCerulean] biosensor was activated, indicating that scFv was misfolding in the periplasm. This observation is consistent with our previous data (see above). pQC2 confirmed that pre^MalE^-scFv activated the [P_ibpA/fxsA_-gfp] biosensor (as observed with pQC1) (Figure 4D). It also indicated that the [P_secM_-mCerulean] biosensor was not activated. This observation indicates that pre^MalE^-scFv is not disrupting traffic through the Sec translocon. When pQC3 was used to monitor the quality of pre^MalE^-scFv, we observed that the [P_secM_-gfp]/[P_cpxP_-mCerulean] biosensors performed similarly to those in pQC1 and pQC2 (Figure 4F). Taken together, these data indicate that pre^MalE^-scFv is efficiently expressed, but at high expression levels it aggregates in the cytoplasm and the periplasm. It does not experience a problem at the Sec translocon.

### Background signal from the biosensors

We monitored the background signal from the biosensors in the toolbox in wild type cells (i.e., cells not producing a recombinant protein) during the growth cycle at 37 °C. We observed that all quality control biosensors were activated during the exponential growth phase, but that the background fluorescence decreased to a low activation level as the cells approached the stationary phase (between 4 and 6 h post inoculation) (Supplementary Figure S7). Users could therefore expect the signal-to-noise ratio to be higher in this window and appropriate controls should be included.

### Induction at lower temperature

Many recombinant protein production experiments are carried out at lower growth temperatures (Structural Genomics Consortium et al., 2008). Some of the experiments presented above were therefore replicated at 25 °C (Supplementary Figure S8). As expected, the biosensors behaved in a similar manner to that observed at 37 °C.

## Conclusions

Herein we present a collection of biosensor plasmids (a toolbox) that can assist researchers to produce recombinant proteins in *E. coli*. The collection is depicted in Figure 1 and its applications summarized in Table 1.

**TABLE 1:** A summary of the biosensors in the toolbox and their applications.

Biosensor	Main applications	Source
[TCD-mCherry]	Calibration of translational output of a recombinant protein	(Zutz et al., 2021)
[TCD-mScarlet-I3]	Calibration of translational output of a recombinant protein. More sensitive than [TCD-mCherry]	This study
[P_ibpA_-gfp]	Monitors the heat shock response. Can be used to detect misfolding of recombinant proteins in the cytoplasm. Can also be used to detect periplasmic proteins that have misfolded before translocation	(Cumming et al., 2024; Zutz et al., 2021)
[P_ibpA/fxsA_-gfp]	Monitors the heat shock response. Can be used to detect misfolding of recombinant proteins in the cytoplasm. Can also be used to detect periplasmic proteins that have misfolded before translocation. More sensitive and higher background than [P_ibpA_-gfp]	(Zutz et al., 2021; Kraft et al., 2007a)
[P_cpxP_-gfp]	Monitors the Cpx response. Can be used to detect misfolding of recombinant proteins in the periplasm	(Cumming et al., 2024)
[P_secM_-gfp]	Monitors the efficiency of co-translational translocation through the Sec translocon	This study
pQC1	Contains the [P_ibpA/fxsA_-gfp]/[P_cpxP_-mCerulean] modules and is designed to monitor protein misfolding in the cytoplasm and periplasm. Improved version of pQC that was published in (Zutz et al., 2021)	This study
pQC2	Contains the [P_ibpA/fxsA_-gfp]/[P_secM_-mCerulean] biosensor modules and is designed to monitor protein misfolding in the cytoplasm and secretion through the Sec translocon	This study
pQC3	Contains the [P_secM_-gfp]/[P_cpxP_-mCerulean] biosensor modules and is designed to monitor secretion through the Sec translocon and protein misfolding in the periplasm	This study

It is important for users to note that there are some limitations when using the biosensors. First of all, there is most likely a threshold for their activation (Cumming et al., 2024; Sørensen and Mortensen, 2005). Low levels of misfolding or inefficient translocation may not immediately activate them. This may be because the cell does not immediately mount a stress response, or because turnover of the fluorescent reporter protein may mask low signals. We have attempted to probe the activation thresholds by titrating recombinant proteins into the cell and looking at the dose response relationship. However, we noted that the threshold response is dependent on the recombinant protein. It is therefore hard to make a general statement about a threshold level. A second limitation is that the biosensors have a limited linear range. Furthermore, strong induction conditions can reduce the biosensor signal, likely due to cellular resource limitations, thereby limiting its use under conditions of high protein expression. Thirdly, the biosensors cannot report on the fate of a misfolded protein, which can change over time with the help of the proteostasis network (Balchin et al., 2016). For example, a protein that is initially misfolded may subsequently be refolded by cellular disaggregases and chaperones, or degraded by proteases. Thus, the output from the biosensors indicates the initial folding state in the cell, and may not always align with experiments that capture the steady-state levels of the recombinant protein. Finally, our data indicate that the background readings in the cell fluctuate (from high to low) during the exponential growth phase. Users are therefore advised to carry out readings in the late exponential phase of growth and to run appropriate controls.

Despite the aforementioned limitations, we believe that the toolbox will be useful for researchers wanting to stream-line the production of recombinant proteins in *E. coli,* as it will enable them to determine when unwanted events have occurred and screen constructs and induction conditions that avoid them. This will reduce cellular stress and have positive effects on protein titers and the production of biomass. It will also be useful to synthetic biologists wanting to screen single cells or build self-regulating genetic circuits designed to avoid unwanted events. These applications have not been explored in this study, but have been demonstrated in other publications (see (Zutz et al., 2021; Dragosits et al., 2012)). Finally, it can be used in parallel with other resources that are used for obtaining folded recombinant proteins in *E. coli*. For example, fusion proteins that increase solubility (Zutz et al., 2021; Sørensen and Mortensen, 2005) or that monitor protein folding (Waldo et al., 1999; Mansell et al., 2010), plasmids that overexpress chaperones and proteases (De Marco et al., 2007; de Marco, 2009; De Marco and De Marco, 2004; De Marco, 2007; Zhang et al., 2014), or strains that monitor cellular capacity (Ceroni et al., 2018).

The biosensor toolbox is available through Addgene (https://www.addgene.org/Daniel_Daley/). In addition to the biosensors, the collection also contains the expression constructs we used. This will allow the community to replicate our experiments and evaluate the performance of other biosensors so that the toolbox can be expanded.

## Methods

### Molecular cloning

Full sequences of the biosensor plasmids used in the study are available in Supplementary Table S2. The construction of the [P_ibpA_-gfp] biosensor was described in (Zutz et al., 2021), and the [P_cpxP_-gfp] biosensor in (Cumming et al., 2024). The construction of all other biosensors is summarized in Supplementary Table S3. Full sequences of the expression plasmids containing the TCD are available in Supplementary Table S4. The construction of these constructs is summarized in Supplementary Table S5. Full sequences of the expression plasmids used to express fluorescent proteins and monitor their maturation and degradation are available in Supplementary Table S6. The construction of these constructs is summarized in Supplementary Table S7. All molecular cloning was carried out using PCR products that had been *DpnI* treated and gel-purified, then ligated using the *in vivo* DNA assembly method (Watson and García-Nafría, 2019) in the *E. coli* MC1061 strain [str.K-12F^−^λ^–^ Δ*(ara-leu)7697* [*araD139*]B/rΔ*(codB-lacI)3 galK16 galE15* e14^–^ *mcrA0 relA1 rpsL150*(Str^R^) *spoT1 mcrB1 hsdR2*(*r* ^–^ *m* ^+^)]. Oligonucleotides were obtained from Eurofins Genomics (Germany) or Genscript (USA). PCR was carried out using the Q5-Polymerase (New England Biolabs, USA). DNA sequencing was performed by Eurofins Genomics (Germany).

### Protein expression

Expression and biosensor plasmids were co-transformed into chemically competent MC1061 cells and selected on LB-Miller agar containing 50 μg/mL kanamycin and 50 μg/mL spectinomycin. Pre-cultures were started by inoculating single colonies into 5 mL of LB-Miller with antibiotics in a 24-well plate and incubated for approximately 16 h at 37 °C with shaking at 180 rpm. The pre-culture was subsequently back-diluted 1:100 into 5 mL of fresh LB-Miller with antibiotics in a 24-well plate and incubated at 37 °C with shaking 180 rpm until the OD_600_ was approximately 0.5 (approx. 3 h). Recombinant proteins were produced by induction with different concentrations of L-arabinose (see text for details) for 3 h at either 25 or 37 °C with shaking 180 rpm. OD_600_ was determined from a 200 µL aliquot of bacterial culture using a SpectraMax *m2e* plate reader (Molecular Devices, U.K.).

### Quantification of fluorescence

Fluorescence was measured from cells only, as we did not detect fluorescence in the culture media. A 1 mL aliquot of the bacterial culture was transferred to a 2.2 mL 96-well plate and cells were pelleted by centrifugation at 3220 *g* for 10 min. The culture medium was removed, and the cells were resuspended in 200 μL of buffer (50 mM Tris–HCl pH 8.0, 200 mM NaCl, 15 mM EDTA). The cell suspension was transferred to a 96-well optical bottom black-wall plate (Thermo Scientific) and the fluorescence was determined in a SpectraMax Gemini EM plate reader (Molecular Devices, U.K.) using the settings listed in Table 2. Fluorescence values were normalized by the optical density of the cultures (OD_600_).

**TABLE 2:** Excitation, emission and filter wavelengths for different fluorescent proteins used in this study.

Fluorescent protein	Excitation wavelength	Filter	Emission wavelength
GFPmut3	485 nm	495 nm	513 nm
mCherry	585 nm	590 nm	610 nm
mScarlet-I3	568 nm	590 nm	600 nm
mCerulean	433 nm	455 nm	475 nm

### Protein maturation and degradation assays

Expression plasmids harboring the coding sequence for a fluorescent protein and degradation tag were transformed into chemically competent MC1061 cells and selected on LB-Miller agar containing 50 μg/mL kanamycin. Pre-cultures were started by inoculating single colonies into 5 mL of LB-Miller with antibiotics in a 24-well plate and incubated for approximately 16 h at 37 °C with shaking at 180 rpm. The pre-culture was subsequently back-diluted 1:100 into 5 mL of fresh LB-Miller with antibiotics in a 24-well plate and incubated at 37 °C with shaking 180 rpm until the OD_600_ was approximately 0.5 (approx. 3 h). Fluorescent proteins were produced by induction with a final concentration of 0.02% (w/v) of L-arabinose for 2 h at 37 °C. For maturation assays: OD_600_ and fluorescence were measured as described above, but 33 μg/mL chloramphenicol was added to the buffer to stop protein production. Fluorescence and OD_600_ readings were then taken every 30 min for 5 h, and in between measurements, the plate was stored at 37 °C covered with PCR foil to avoid evaporation. For degradation assays: protein production was downshifted by centrifugation at 3220 *g* for 10 min, the media was discarded and replaced with fresh LB-Miller medium containing 50 μg/mL kanamycin and 2% (w/v) glucose. The OD_600_ was measured as described above. Fluorescence was measured by directly transferring the cell suspension into a 96-well optical bottom black-wall plate (Thermo Scientific). For background determination plasmid free cultures of MC1061 in LB-Miller were processed the same way.

### Cellular fractionations

Cultures were scaled up to 25 mL in 250 mL Erlenmeyer flasks. Cell pellets were harvested at 3220 x *g* for 15 min at 4 °C and resuspended in 25 mL of Tris-buffer (50 mM Tris, pH 8.3, 100 mM NaCl). The suspension was lysed for 3 cycles using an Emulsiflex C3 pressure homogenizer between 1000 and 1500 bar. Lysed samples were centrifuged at 3220 *g* for 15 min at 4 °C to remove unbroken cells. A 150 µL aliquot of the supernatant was then saved as the total fraction. A 15 mL aliquot of the remaining supernatant was transferred to a Beckman centrifuge tube and centrifuged at 22000 *g* for 1 h at 4 °C (Avanti J-26S with JA-25.50 rotor, Beckman coulter) and a 150 µL aliquot of the supernatant was saved as the soluble fraction. The pellet was resuspended in 15 mL of Tris-buffer, and a 150 µL aliquot was saved as the insoluble fraction.

### SDS-PAGE and western blotting

A 200 µL aliquot of the culture was pelleted by centrifugation at 17000 *g* for 3 min. The cell pellet was then resuspended in 200 µL 1x Laemmli buffer and heated at 95 °C for 10 min. An aliquot corresponding to 0.05 OD_600_ units was then analyzed by SDS-PAGE. When cells had been fractionated, a 150 µL aliquot of the sample was mixed with 50 µL 4x Laemmli buffer. SDS-PAGE was performed using 1 mm 12% Tris-glycine acrylamide gels run on the Hoefer Mighty Small II Mini Vertical Electrophoresis System and all gels were run at 180 V for 70 min (running buffer: 25 mM Tris, 192 mM glycine 1% SDS (w/v)).

### Western blotting

Proteins were transferred from the SDS-PAGE to a nitrocellulose membrane using a semidry Trans-Blot SD cell (Bio-Rad) for 45 min at 15 V. The membrane was then blocked overnight in 5% (w/v) non-fat milk (PanReac AppliChem) in Tris-buffered saline (TBS) (50 mM Tris pH 7.4, 200 mM NaCl) then incubated with HIS Lite™ Cy3 Bis NTA-Ni Complex or HIS Lite™ Cy5 Bis NTA-Ni Complex (AAT Bioquest) for 1 h at a working concentration of 1 µM. Membranes were imaged using the appropriate channel on an Azure c600 Imaging System.

## Data Availability

The original contributions presented in the study are included in the article/Supplementary Material, further inquiries can be directed to the corresponding author.
