# Supplementary material for: A biosensor toolbox for monitoring recombinant protein production in Escherichia coli
Source: Front Bioeng Biotechnol. 2026 Oct 7;14:1956994. doi: 10.3389/fbioe.2026.1956994 (PMC13683788; doi:10.3389/fbioe.2026.1956994)
Supplement: Supplementary file 1 [file DataSheet1.pdf]

A Biosensor Toolbox for Monitoring Recombinant Protein Production in *Escherichia coli*

Maurice Mürköster[1], Mateusz Balka[1], Christina J. Kamp[1], Judit Källström[1], Linus Karlsson[1], Claudia Rodriguez Aparicio[1], Erik Tunäng[1], Hanna-Louise Ögren[1], Silvia Theopold[1], Alister J. Cumming[1], Alex Toftgaard Nielsen[2] and Daniel O. Daley[1]

[1] Department of Biochemistry and Biophysics, Stockholm University, SE-19468, Sweden.

[2] The Novo Nordisk Foundation Center for Biosustainability, Technical University of Denmark, 2800 Kongens Lyngby, Denmark.

Correspondence: DOD (ddaley@dbb.su.se, +46 8 162 910)

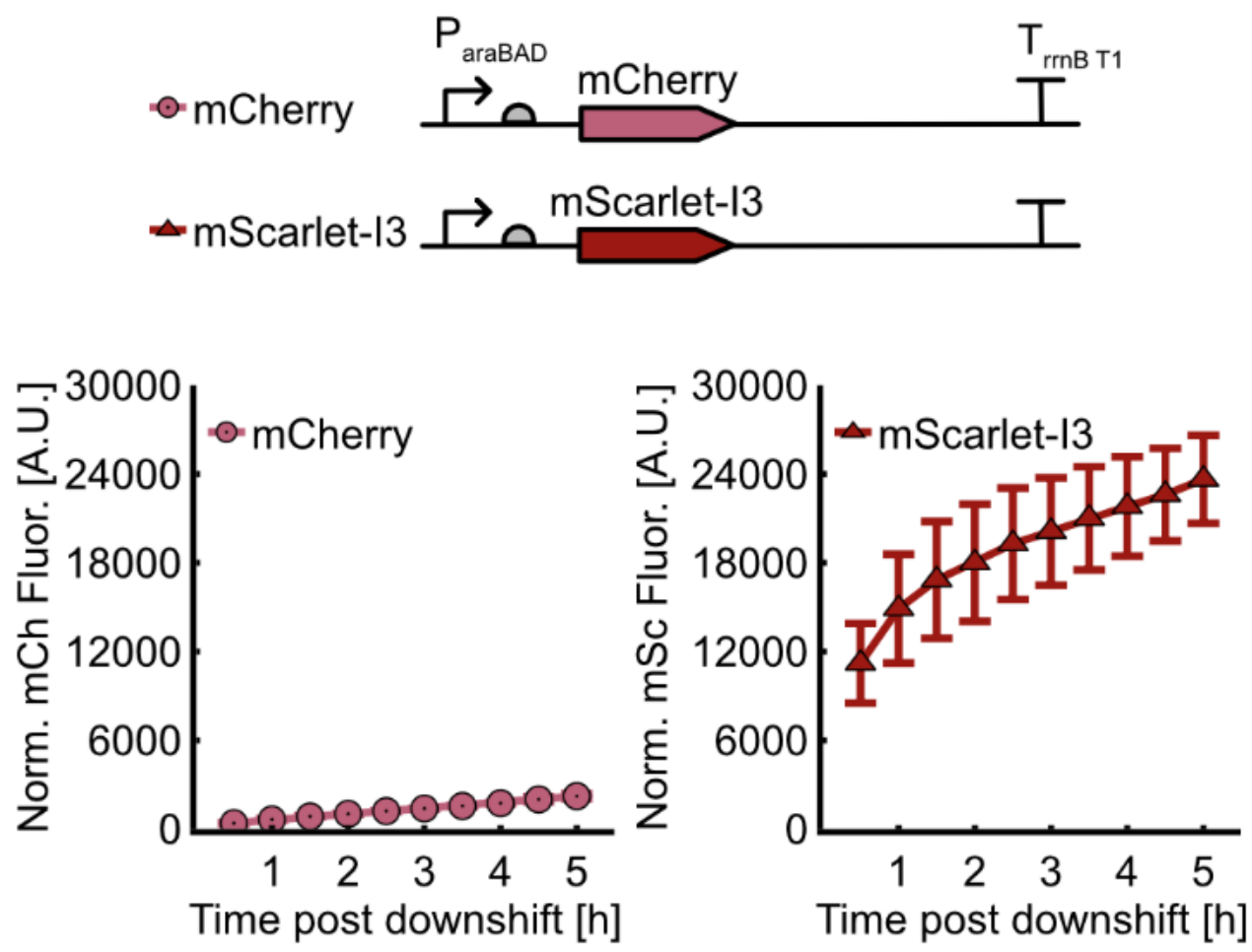

Figure S1. A comparison of the brightness and maturation of mCherry and mScarlet-I3. Cartoons of plasmids used in the experiments are shown above each panel. mCherry and mScarlet-I3 were expressed from a pBAD expression vector with 0,02 % (w/v) L-arabinose. After two hours of induction, chloramphenicol (Cm) was added to inhibit protein synthesis and fluorescence was followed in 30-minute intervals for 5 hours. Fluorescence data was normalized to the OD_{600}. All data presented as mean ± standard deviation (s.d.) (n ≥ 3).

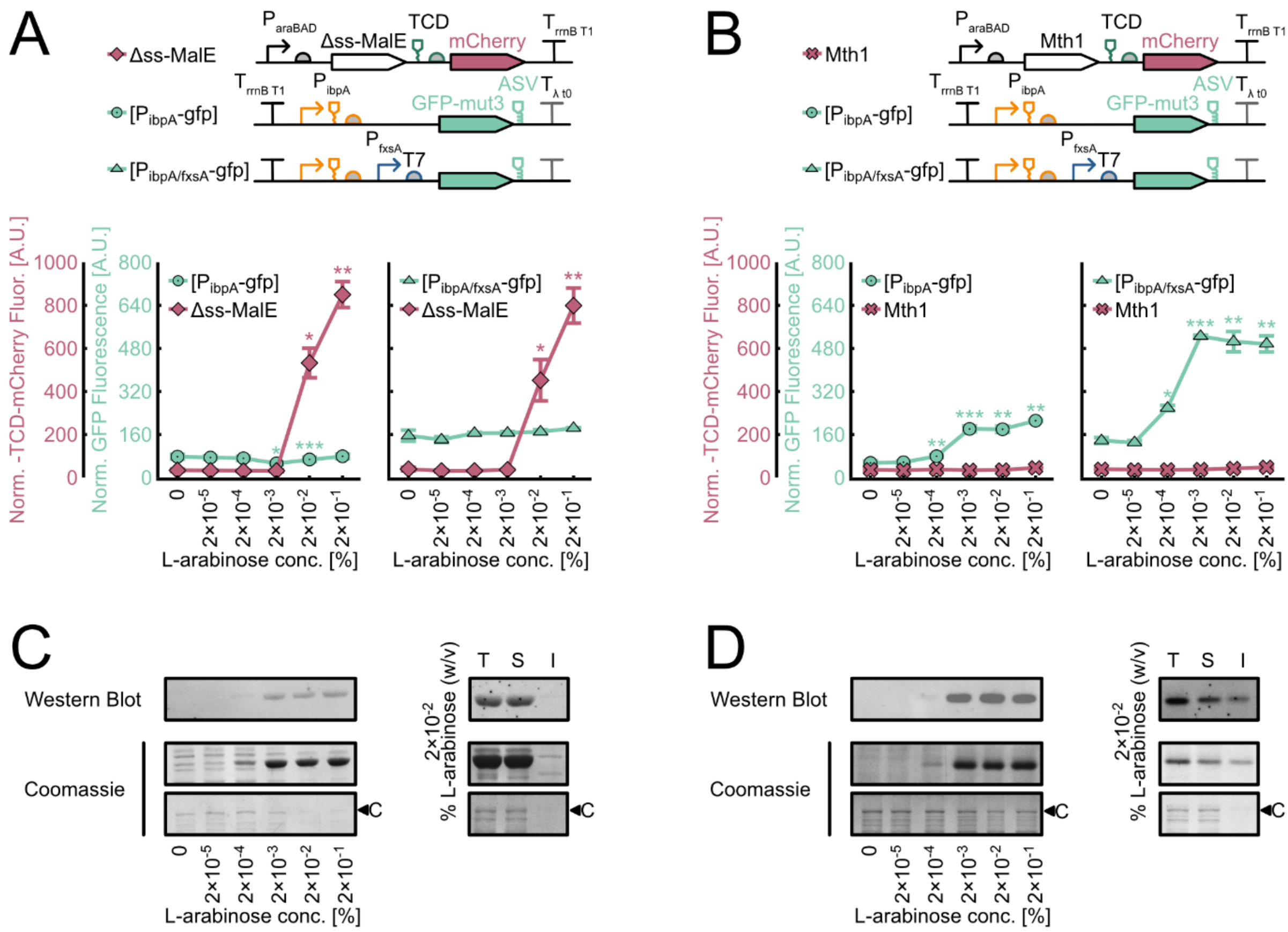

Figure S2. Misfolding of recombinant proteins in the cytoplasm can be detected with [P_{ibpA}-gfp] and [$P_{ibpA/fxsA}$-gfp]. Cartoons of plasmids used in the experiments are shown above each panel. **(A)** Δss-MalE (an example of a folded protein) and **(B)** Mth1 (an example of a misfolding protein) were expressed in the cytoplasm with increasing concentrations of L-arabinose. The fluorescence output from the translationally coupled mCherry was normalized by the OD_{600} (red). The fluorescence output from [P_{ibpA}-gfp] and [$P_{ibpA/fxsA}$-gfp] was normalized by the OD_{600} (green). OD_{600} values are presented in Supplementary information, Table S1. All data presented as mean ± standard deviation (s.d.) (n ≥ 3). A statistically significant difference between the uninduced sample (0 % (w/v) L-arabinose) and the induced sample is marked. $P < 0.05$ is denoted by *, $P < 0.01$ by **, and $P < 0.001$ by *** (two-tailed Student's t-test). Data where no statistical difference was observed is not marked. **(C)** The steady state levels of Δss-MalE and **(D)** Mth1 were monitored by separating proteins by SDS-PAGE followed by Coomassie staining or Western blotting. As all proteins contained a C-terminal poly-His tag they were detected with the HIS Lite™ Cy3 Bis NTA-Ni Complex. The solubility of Δss-MalE and Mth1 was investigated. Cells were lysed to obtain the total sample (T). T was then fractionated into soluble (S) and insoluble (I) fractions by centrifugation. The fractions were then analyzed by SDS-PAGE followed by Coomassie staining or Western blotting. A loading control on the Coomassie stained SDS-PAGE is denoted C.

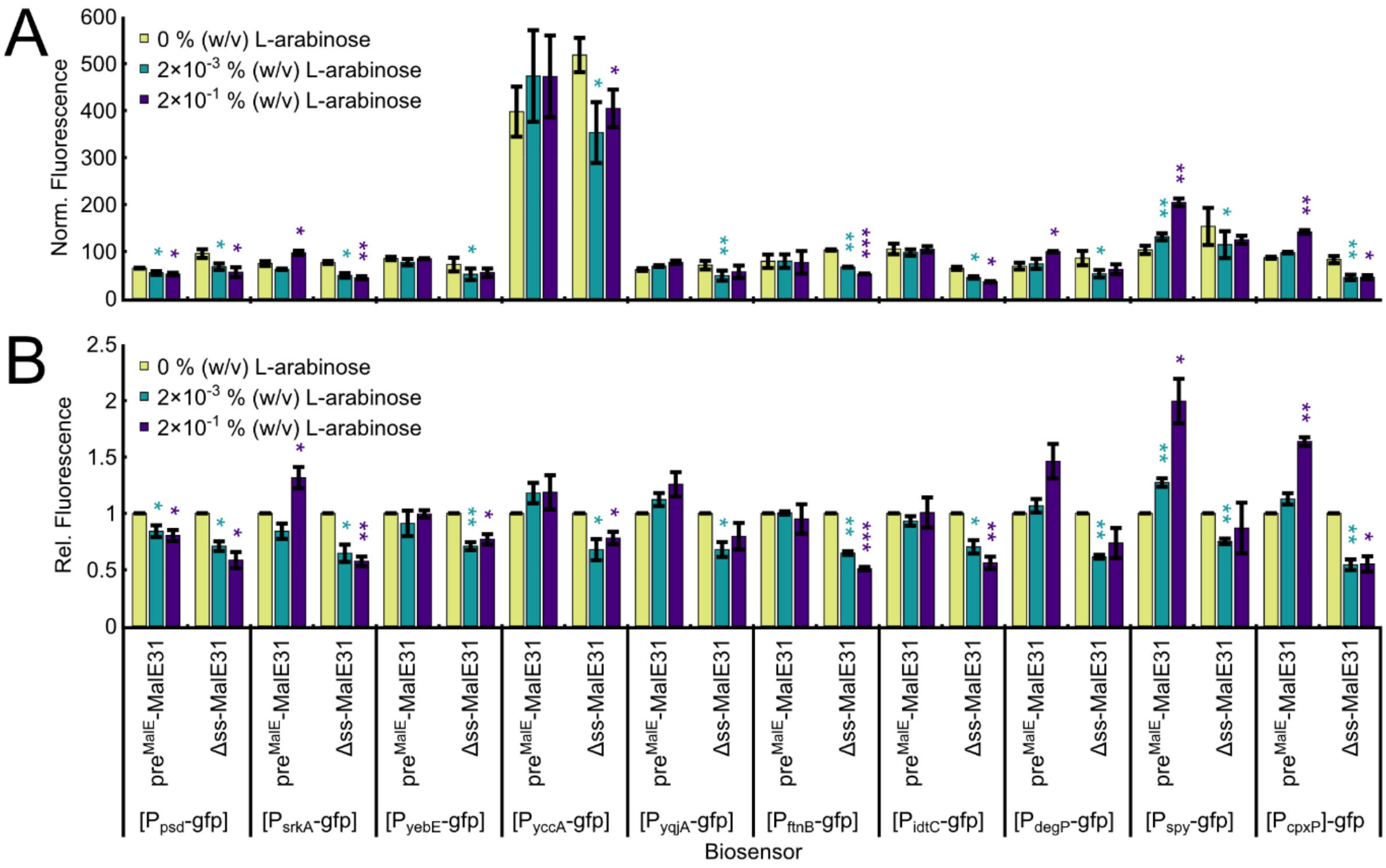

Figure S3. Evaluation of different Cpx-based biosensors. Ten Cpx-based biosensors were constructed by cloning known Cpx responsive promoters (chosen from [10]) upstream of the coding sequence of a fast-folding and fast-degrading version of the Green Fluorescent Protein (GFP-mut3$_{ASV}$) in the pSEVA631(sp) backbone. Each biosensor was evaluated by titrating pre^{MalE}-MalE31 with two different concentrations of L-arabinose. pre^{MalE}-MalE31 is targeted to the periplasm where it is known to aggregate and activate the Cpx envelope stress response [5,11]. As a negative control we titrated Δss-MalE31, which lacks the signal peptide and aggregates in the cytoplasm [5,11]. It is therefore not expected to activate the Cpx envelope stress response. **(A)** In each experiment fluorescence was measured using an excitation wavelength of 475 nm, a filter of 495 nm and emission wavelength of 515 nm and the output was normalized to the OD_{600}. All data presented as mean ± standard deviation (s.d.) (n ≥ 3). A statistically significant difference between the uninduced sample (0 % (w/v) L-arabinose) and the induced sample is marked. P < 0.05 is denoted by *, P < 0.01 by **, and P < 0.001 by *** (two-tailed Student's t-test). Data where no statistical difference was observed is not marked. **(B)** As per panel A except that fluorescence output per OD_{600} was expressed relative to the 0 % sample (i.e. no induction).

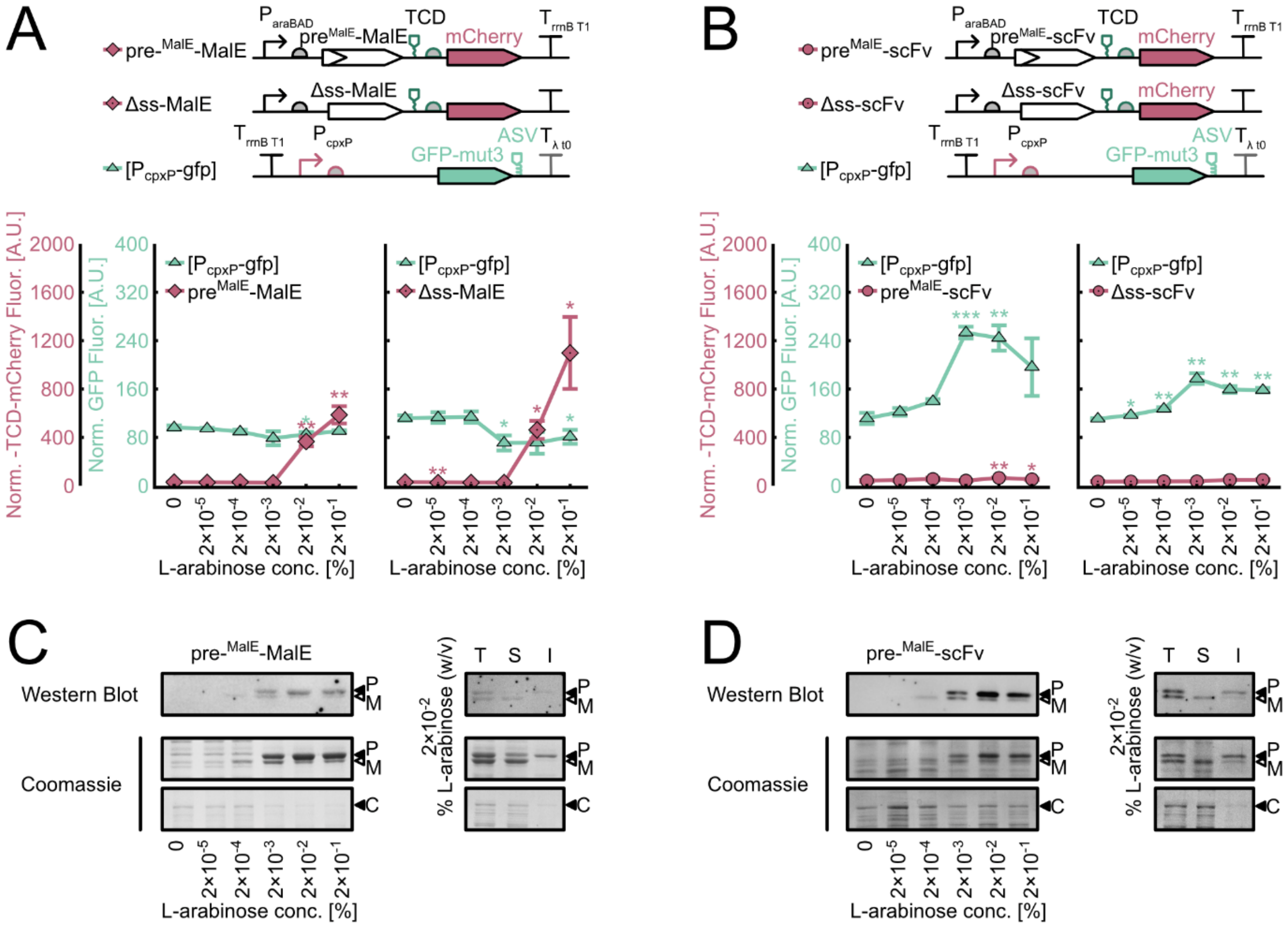

Figure S4. Misfolding of proteins in the periplasm can be detected with [P_{cpxP}-gfp]. Cartoons of plasmids used in the experiments are shown above each panel. **(A)** pre^{MalE}-MalE (an example of a folded periplasmic protein) and **(B)** pre^{MalE}-scFv (an example of a misfolding periplasmic protein) were expressed with increasing concentrations of L-arabinose. The fluorescence output from the translationally coupled mCherry was normalized by the OD_{600} (red). The fluorescence output from [P_{cpxP}-gfp] and [P_{spy}-gfp] was normalized by the OD_{600} (green). OD_{600} values are presented in Supplementary information, Table S1. All data presented as mean ± standard deviation (s.d.) ($n \geq 3$). A statistically significant difference between the uninduced sample (0 % (w/v) L-arabinose) and the induced sample is marked. $P < 0.05$ is denoted by *, $P < 0.01$ by **, and $P < 0.001$ by *** (two-tailed Student's t-test). Data where no statistical difference was observed is not marked. As a negative control the cytoplasmic control Δss-MalE and Δss-scFv, which should not activate the biosensor, were also expressed. **(C)** The steady state levels of pre^{MalE}-MalE and **(D)** pre^{MalE}-scFv were monitored by separating proteins by SDS-PAGE followed by Coomassie staining or Western blotting. This experiment also indicated whether the signal peptide had been retained (indicative of a cytoplasmic location). As proteins enter the periplasm, the signal peptide is cleaved off [61], thus the presence / absence of the signal peptide can be used as a proxy for the location of the protein [20]. Signal peptide containing versions of each protein (localized to the cytoplasm) are denoted P, and the mature versions (localized to the periplasm) are denoted M. As all proteins contained a C-terminal poly-His tag they were detected with the HIS Lite™ Cy3 Bis NTA-Ni Complex. The solubility of pre^{MalE}-MalE and pre^{MalE}-scFv was investigated. Cells were lysed to obtain the total sample (T). T was then fractionated into soluble (S) and insoluble (I) fractions by centrifugation. The fractions were then analyzed by SDS-PAGE followed by Coomassie staining or Western blotting. A loading control on the Coomassie stained SDS-PAGE is denoted C. pre^{MalE}-MalE and pre^{MalE}-scFv panels are the same as those shown in Figure 3C and 3D respectively. They were included so a comparison can be made.

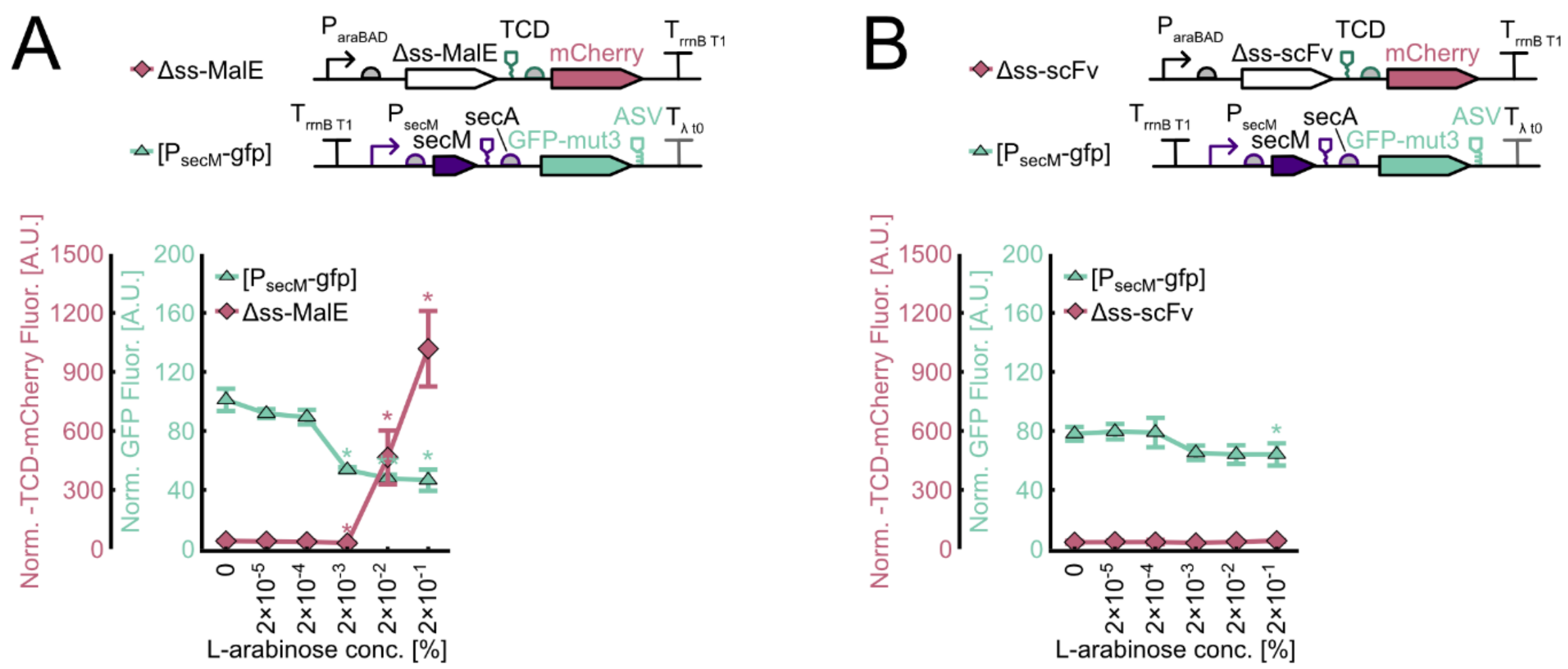

Figure S5. Control experiments for the [P_{secM}-gfp] biosensor. Cartoons of plasmids used in the experiments are shown above each panel. **(A)** Δss-MalE was expressed with increasing concentrations of L-arabinose. The fluorescence output from the translationally coupled mCherry was normalized by the OD_{600} (red). The fluorescence output from the [P_{secM}-gfp] biosensor was normalized by the OD_{600} (green). All data presented as mean ± standard deviation (s.d.) (n ≥ 3). A statistically significant difference between the uninduced sample (0 % (w/v) L-arabinose) and the induced sample is marked. $P < 0.05$ is denoted by *, $P < 0.01$ by **, and $P < 0.001$ by *** (two-tailed Student's t-test). Data where no statistical difference was observed is not marked. **(B)** As per (A) except that Δss-scFv was expressed.

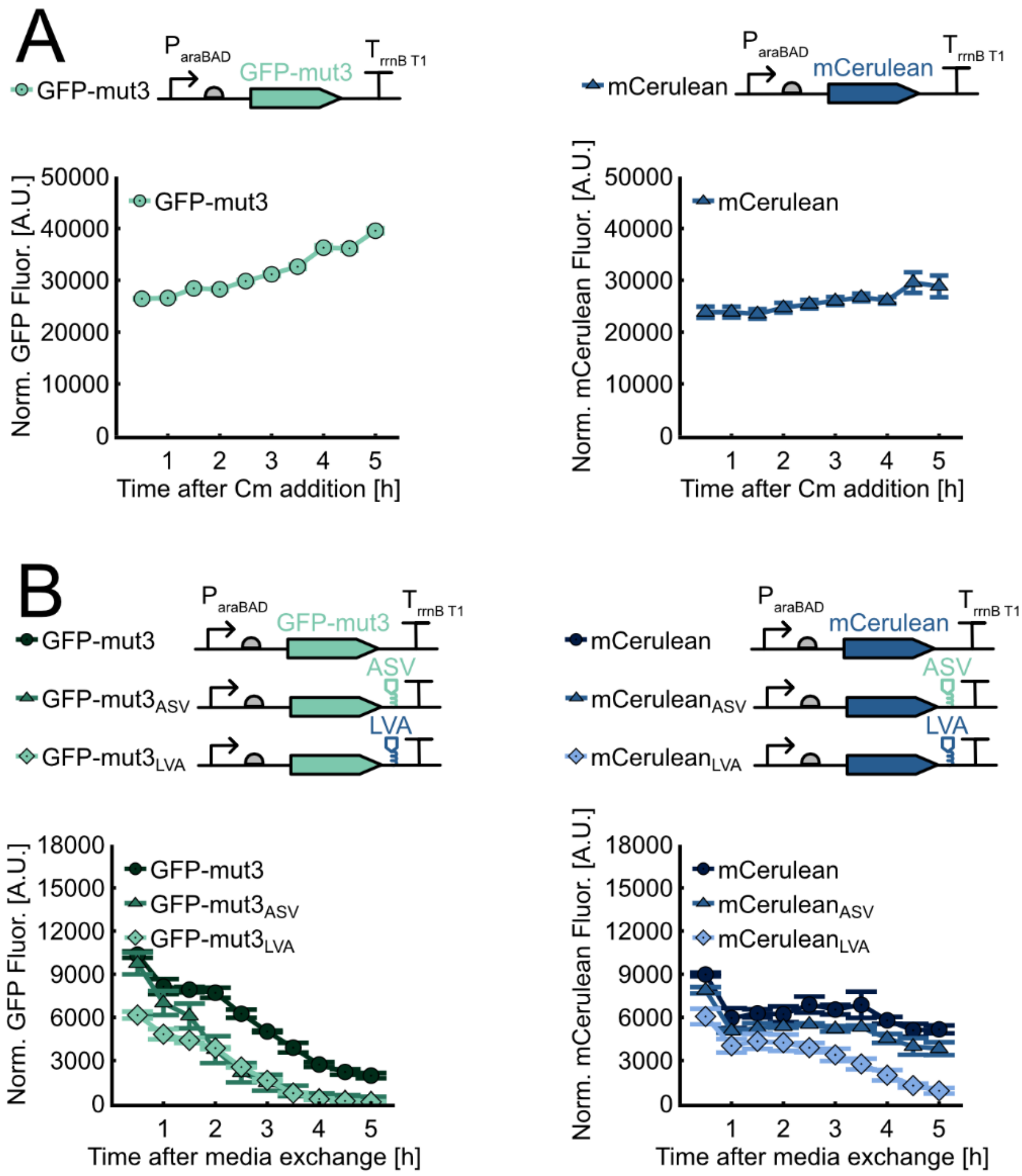

Figure S6. Evaluation of the mCerulean fluorescent protein for a 2-color biosensor. Cartoons of plasmids used in the experiments are shown above each panel. **(A)** To assess brightness and maturation, GFP-mut3 and mCerulean were expressed from a pBAD expression vector with 0,02 % (w/v) L-arabinose final concentration. After two hours of induction, chloramphenicol (Cm) was added to inhibit protein synthesis and fluorescence was followed in 30-minute intervals for 5 hours. **(B)** To shorten the half-life of GFP-mut3 and mCerulean, "ASV" and "LVA" ssrA degradation tags were added [29]. To assess the efficiency of degradation, GFP-mut3 and mCerulean were expressed from a pBAD expression vector with 0,02 % (w/v) L-arabinose final concentration. After two hours of induction, LB-medium containing L-arabinose was exchanged for LB-medium containing 2 % (w/v) glucose. OD_{600} and fluorescence was measured in 30-minute intervals for 5 hours.

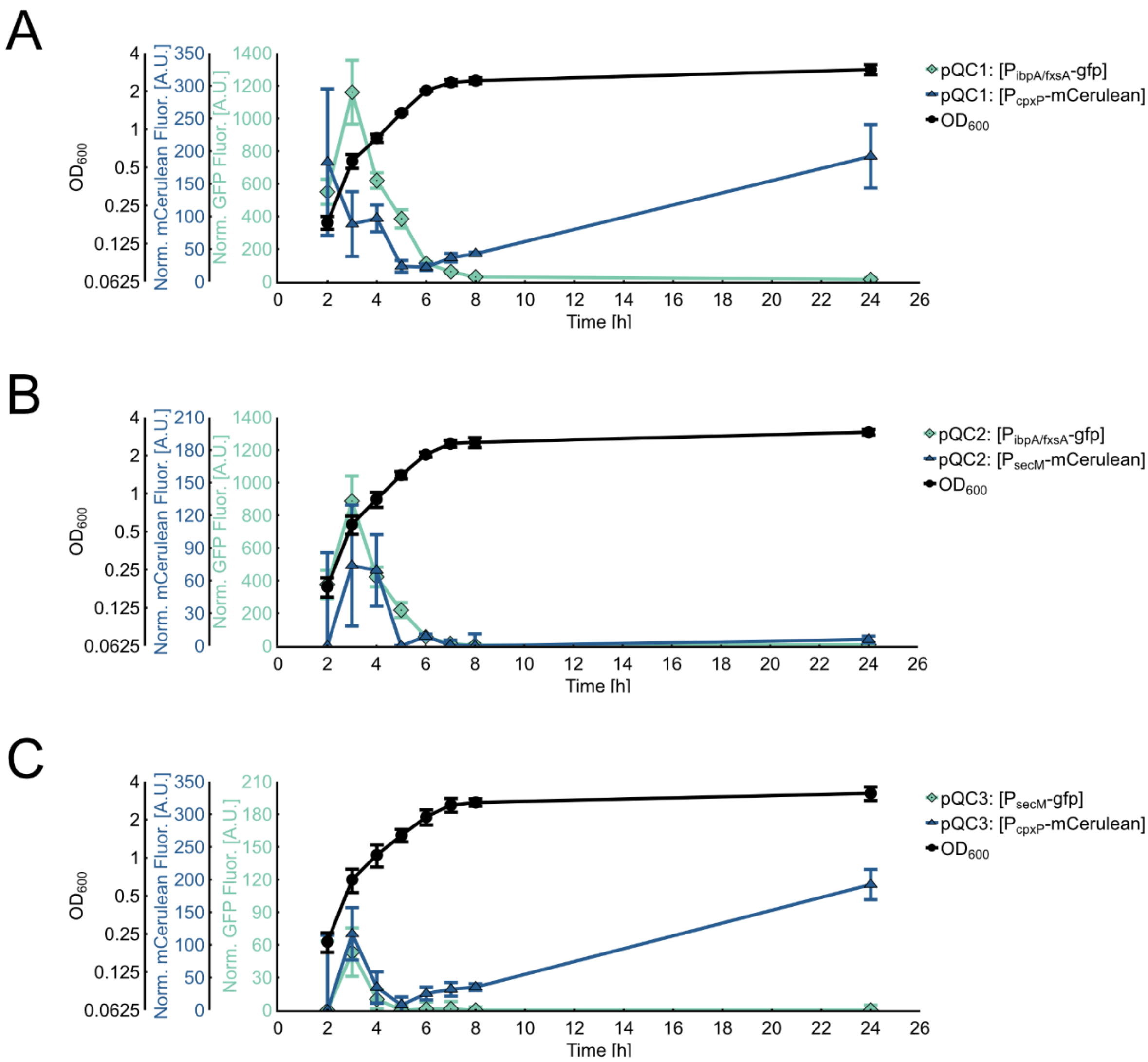

Figure S7. Background fluorescence from each biosensor in the collection was measured during growth of *E. coli*. Fluorescence output from each biosensor module was normalized by OD_{600}. A representative growth curve is marked in black. Data presented as mean ± standard deviation (s.d.) (n ≥ 3). **(A)** pQC1, **(B)** pQC2 and **(C)** pQC3.

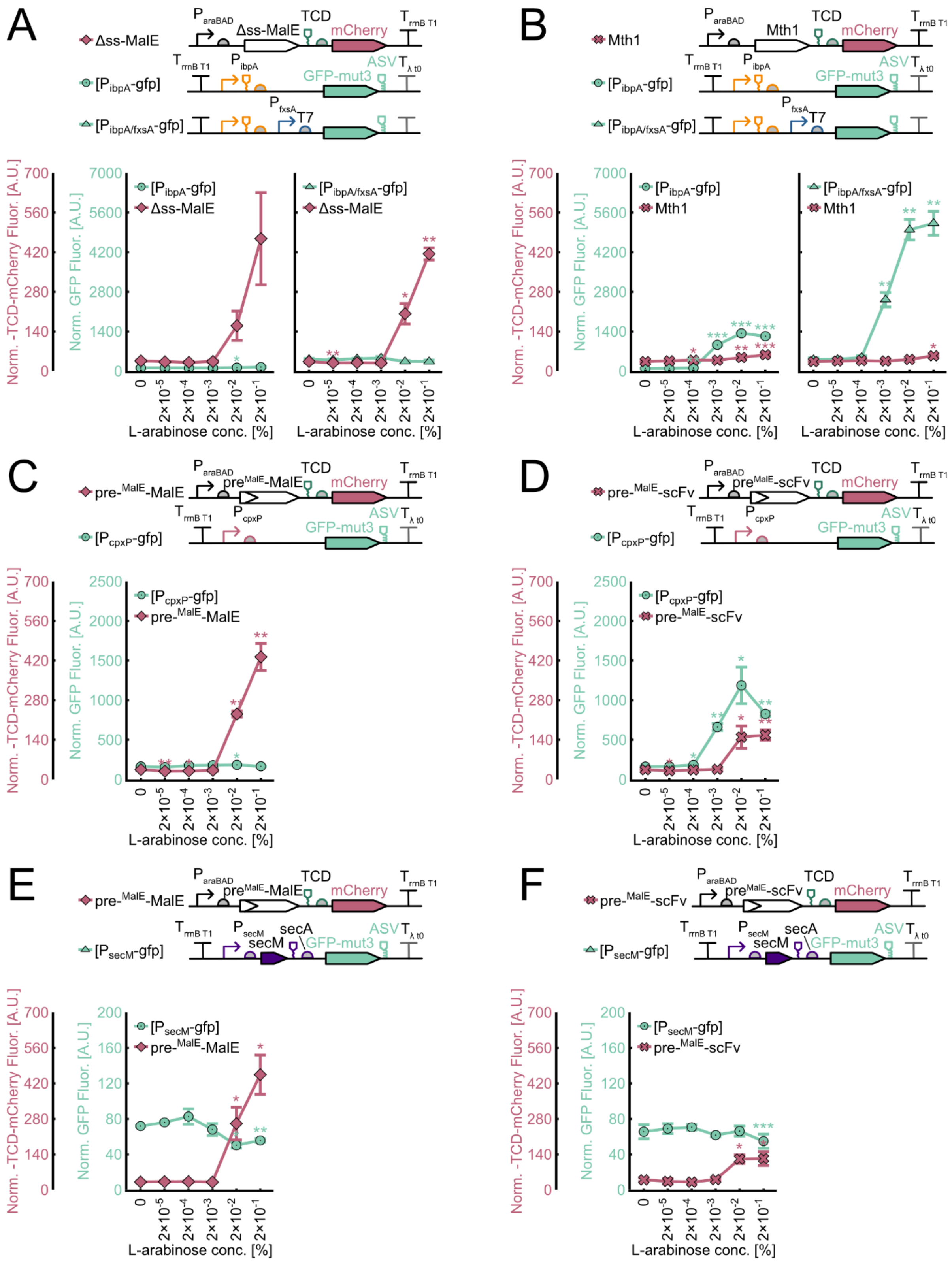

Figure S8. Evaluation of selected biosensors at 25 °C. Cartoons of plasmids used in the experiments are shown above each panel. **(A)** Δss-MalE (an example of a folded cytoplasmic protein) and **(B)** Mth1 (an example of a misfolding cytoplasmic protein) were expressed in the cytoplasm with increasing concentrations of L-arabinose. The fluorescence output from the translationally coupled mCherry was normalized by the OD_{600} (red). The fluorescence output from [P_{ibpA}-gfp] and [$P_{ibpA/fxsA}$-gfp] was normalized by the OD_{600} (green). All data presented as mean ± standard deviation (s.d.) (n ≥ 3). A statistically significant difference between the uninduced sample (0 % (w/v) L-arabinose) and the induced sample is marked. P < 0.05 is denoted by *, P < 0.01 by **, and P < 0.001 by *** (two-tailed Student's t-test). Data where no statistical difference was

observed is not marked. **(C)** As per panel except (A) that pre^{MalE}-MalE (an example of a folded periplasmic protein) was expressed and [P_{cpxP}-gfp] was used as a biosensor. **(D)** As per panel (C) except that pre^{MalE}-scFv (an example of a misfolding periplasmic protein) was expressed. **(E)** As per panel (C) except that [P_{secM}-gfp] was used as the biosensor. **(F)** As per panel (D) except that [P_{secM}-gfp] was used as the biosensor.

Table S1. Final OD_{600} values from experiments shown in the main manuscript.

Figure	Protein expressed	Biosensor	L-ara conc. [%]	OD	Standard deviation
2A	Δss-MalE	[TCD-mCherry]	0	1.497893	0.065558977
2A	Δss-MalE	[TCD-mCherry]	0.000195313	1.450872	0.047145783
2A	Δss-MalE	[TCD-mCherry]	0.000390625	1.610071	0.121035996
2A	Δss-MalE	[TCD-mCherry]	0.00078125	1.623893	0.085703663
2A	Δss-MalE	[TCD-mCherry]	0.0015625	1.840572	0.095245941
2A	Δss-MalE	[TCD-mCherry]	0.003125	1.974592	0.115621222
2A	Δss-MalE	[TCD-mCherry]	0.00625	2.336225	0.103808807
2A	Δss-MalE	[TCD-mCherry]	0.0125	2.208761	0.128865325
2A	Δss-MalE	[TCD-mCherry]	0.025	2.193085	0.044561928
2A	Δss-MalE	[TCD-mCherry]	0.05	2.063784	0.147711134
2A	Δss-MalE	[TCD-mCherry]	0.1	1.925357	0.157540624
2A	Δss-MalE	[TCD-mCherry]	0.2	2.01842	0.18106103
2A	Δss-MalE	[TCD-mScarlet-I3]	0	1.494904	0.079285676
2A	Δss-MalE	[TCD-mScarlet-I3]	0.000195313	1.612812	0.053318141
2A	Δss-MalE	[TCD-mScarlet-I3]	0.000390625	1.593779	0.02892449
2A	Δss-MalE	[TCD-mScarlet-I3]	0.00078125	1.688571	0.022239212
2A	Δss-MalE	[TCD-mScarlet-I3]	0.0015625	1.688189	0.104694959
2A	Δss-MalE	[TCD-mScarlet-I3]	0.003125	1.71998	0.058313271
2A	Δss-MalE	[TCD-mScarlet-I3]	0.00625	1.945033	0.086124859
2A	Δss-MalE	[TCD-mScarlet-I3]	0.0125	1.708465	0.135076729
2A	Δss-MalE	[TCD-mScarlet-I3]	0.025	1.705296	0.115338003
2A	Δss-MalE	[TCD-mScarlet-I3]	0.05	1.660321	0.03976668
2A	Δss-MalE	[TCD-mScarlet-I3]	0.1	1.66588	0.064019413
2A	Δss-MalE	[TCD-mScarlet-I3]	0.2	1.718377	0.081645738
2B	Mth1	[TCD-mCherry]	0	1.496545	0.015146969
2B	Mth1	[TCD-mCherry]	0.000195313	1.396615	0.035081023
2B	Mth1	[TCD-mCherry]	0.000390625	1.356061	0.006908564
2B	Mth1	[TCD-mCherry]	0.00078125	1.453807	0.103943162
2B	Mth1	[TCD-mCherry]	0.0015625	1.552153	0.080781856
2B	Mth1	[TCD-mCherry]	0.003125	1.672247	0.03115789
2B	Mth1	[TCD-mCherry]	0.00625	1.893836	0.072493397
2B	Mth1	[TCD-mCherry]	0.0125	1.850048	0.06862294
2B	Mth1	[TCD-mCherry]	0.025	1.862331	0.050950891
2B	Mth1	[TCD-mCherry]	0.05	1.783201	0.08238737
2B	Mth1	[TCD-mCherry]	0.1	1.698849	0.091549702
2B	Mth1	[TCD-mCherry]	0.2	1.963229	0.160286106
2B	Mth1	[TCD-mScarlet-I3]	0	1.43398	0.029580001
2B	Mth1	[TCD-mScarlet-I3]	0.000195313	1.480804	0.035119389
2B	Mth1	[TCD-mScarlet-I3]	0.000390625	1.453569	0.054538784
2B	Mth1	[TCD-mScarlet-I3]	0.00078125	1.541049	0.096052284
2B	Mth1	[TCD-mScarlet-I3]	0.0015625	1.606296	0.120544391

2B	Mth1	[TCD-mScarlet-I3]	0.003125	1.905937	0.275664442
2B	Mth1	[TCD-mScarlet-I3]	0.00625	1.699313	0.071585064
2B	Mth1	[TCD-mScarlet-I3]	0.0125	1.735419	0.058203574
2B	Mth1	[TCD-mScarlet-I3]	0.025	1.597331	0.021581803
2B	Mth1	[TCD-mScarlet-I3]	0.05	1.637048	0.044697825
2B	Mth1	[TCD-mScarlet-I3]	0.1	1.636688	0.071000768
2B	Mth1	[TCD-mScarlet-I3]	0.2	1.649513	0.053996348
3A	pre-MalE-MalE	[PsecM-gfp]	0	1.445389	0.035714952
3A	pre-MalE-MalE	[PsecM-gfp]	0.00002	1.20348	0.132487841
3A	pre-MalE-MalE	[PsecM-gfp]	0.0002	1.210377	0.092743034
3A	pre-MalE-MalE	[PsecM-gfp]	0.002	1.560167	0.302799222
3A	pre-MalE-MalE	[PsecM-gfp]	0.02	1.647344	0.191937546
3A	pre-MalE-MalE	[PsecM-gfp]	0.2	1.584884	0.13825349
3A	pre-DsbA-MalE	[PsecM-gfp]	0	1.498019	0.04740954
3A	pre-DsbA-MalE	[PsecM-gfp]	0.00002	1.323856	0.056813138
3A	pre-DsbA-MalE	[PsecM-gfp]	0.0002	1.33842	0.031985847
3A	pre-DsbA-MalE	[PsecM-gfp]	0.002	1.415964	0.102424791
3A	pre-DsbA-MalE	[PsecM-gfp]	0.02	0.780388	0.06942822
3A	pre-DsbA-MalE	[PsecM-gfp]	0.2	0.571417	0.03594454
3B	pre-MalE-scFv	[PsecM-gfp]	0	1.442691	0.08093735
3B	pre-MalE-scFv	[PsecM-gfp]	0.00002	1.417789	0.103703158
3B	pre-MalE-scFv	[PsecM-gfp]	0.0002	1.452531	0.130819988
3B	pre-MalE-scFv	[PsecM-gfp]	0.002	1.447916	0.092749193
3B	pre-MalE-scFv	[PsecM-gfp]	0.02	1.524489	0.074695692
3B	pre-MalE-scFv	[PsecM-gfp]	0.2	1.455743	0.134493812
3B	pre-DsbA-scFv	[PsecM-gfp]	0	1.347359	0.062757666
3B	pre-DsbA-scFv	[PsecM-gfp]	0.00002	1.414904	0.130817731
3B	pre-DsbA-scFv	[PsecM-gfp]	0.0002	1.375541	0.11296812
3B	pre-DsbA-scFv	[PsecM-gfp]	0.002	1.192931	0.070049014
3B	pre-DsbA-scFv	[PsecM-gfp]	0.02	0.644305	0.063848195
3B	pre-DsbA-scFv	[PsecM-gfp]	0.2	0.473085	0.050460854
4A	pre-MalE-MalE	pQC1	0	1.293388	0.203056588
4A	pre-MalE-MalE	pQC1	0.00002	1.313408	0.070079738
4A	pre-MalE-MalE	pQC1	0.0002	1.362387	0.1068172
4A	pre-MalE-MalE	pQC1	0.002	1.377057	0.139151411
4A	pre-MalE-MalE	pQC1	0.02	1.534515	0.161130557
4A	pre-MalE-MalE	pQC1	0.2	1.561808	0.215468566
4B	pre-MalE-scFv	pQC1	0	1.128765	0.03721771
4B	pre-MalE-scFv	pQC1	0.00002	1.291729	0.071151797
4B	pre-MalE-scFv	pQC1	0.0002	1.317853	0.083354664
4B	pre-MalE-scFv	pQC1	0.002	1.325921	0.132234366
4B	pre-MalE-scFv	pQC1	0.02	1.135983	0.074636012
4B	pre-MalE-scFv	pQC1	0.2	1.061511	0.042344152
4C	pre-MalE-MalE	pQC2	0	1.156172	0.070887492

4C	pre-MalE-MalE	pQC2	0.00002	1.175111	0.0242991
4C	pre-MalE-MalE	pQC2	0.0002	1.133405	0.083001759
4C	pre-MalE-MalE	pQC2	0.002	1.401272	0.073510821
4C	pre-MalE-MalE	pQC2	0.02	1.468567	0.074615442
4C	pre-MalE-MalE	pQC2	0.2	1.519284	0.157055128
4D	pre-MalE-scFv	pQC2	0	1.077013	0.077183374
4D	pre-MalE-scFv	pQC2	0.00002	1.113103	0.015534455
4D	pre-MalE-scFv	pQC2	0.0002	1.128281	0.040958085
4D	pre-MalE-scFv	pQC2	0.002	1.0436	0.057267776
4D	pre-MalE-scFv	pQC2	0.02	0.973044	0.011687738
4D	pre-MalE-scFv	pQC2	0.2	1.022437	0.039239843
4E	pre-MalE-MalE	pQC3	0	1.150685	0.035147753
4E	pre-MalE-MalE	pQC3	0.00002	1.109973	0.014631564
4E	pre-MalE-MalE	pQC3	0.0002	1.190004	0.093579742
4E	pre-MalE-MalE	pQC3	0.002	1.304056	0.22064931
4E	pre-MalE-MalE	pQC3	0.02	1.35074	0.064434428
4E	pre-MalE-MalE	pQC3	0.2	1.392641	0.01746
4F	pre-MalE-scFv	pQC3	0	1.143712	0.146741146
4F	pre-MalE-scFv	pQC3	0.00002	1.1244	0.155869547
4F	pre-MalE-scFv	pQC3	0.0002	1.148775	0.167396662
4F	pre-MalE-scFv	pQC3	0.002	1.128832	0.11787825
4F	pre-MalE-scFv	pQC3	0.02	1.060252	0.104261274
4F	pre-MalE-scFv	pQC3	0.2	1.170248	0.093760222

Table S2. Sequences of the biosensor plasmids used in the study. *For [P_{ibpA}-gfp] the entire plasmid sequence is shown. The biosensor module is shaded grey. The promoter sequence and 5'UTR is marked in orange font and the sequence for GFP-mut3$_{ASV}$ in green font. For other individual biosensors, only the promoter and 5'UTR, sequence is shown, as the remainder of the sequence is the same. An exception is [P_{spy}-gfp], where the TTAATTAA sequence upstream of the promoter is absent. Note that [P_{secM}-gfp] also contains the coding sequence for secM (underlined). For pQC1, pQC2 and pQC3 the entire plasmid sequence is shown. Annotations are described above. In addition, the second biosensor module is shaded yellow and the sequence for mCerulean$_{LVA}$ in blue font.*

**Biosensor**	**Linear sequence**
[P_{ibpA}-gfp]	ATTTGTCCTACTCAGGAGAGCGTTCACCGACAAACAACAGATAAAACGAAAGGCCCAGTCTTT<br>CGACTGAGCCTTTCGTTTTATTTGATGCCTTTAATTAAAATTCATCTGTTGATCGTGGGTGTTGG<br>CCTGATGAGTTATAGCGATCCCTTGCTGAAAATAACATCATCATTACGTCGCACTGTGGCGGCT<br>ATCGCACTTTAACGTTTCGTGCTGCCCCCTCAGTCTATGCAATAGACCATAAACTGCAAAAAAA<br>AGTCCGCTGATAAGGCTTGAAAAGTTCATTTCCAGACCCATTTTTACATCGTAGCCGATGAGGA<br>CGCGCCTGATGGGTGTTCTGGCTACCTGACCTGTCCATTGTGGAAGGTCTTACATTCTCGCTGA<br>TTTCAGGAGCTATCTAGAATGCGTAAAGGAGAAGAACTTTTCACTGGAGTTGTCCCAATTCTTG<br>TTGAATTAGATGGTGATGTTAATGGGCACAAATTTTCTGTCAGTGGAGAGGGTGAAGGTGAT<br>GCAACATACGGAAAACTTACCCTTAAATTTATTTGCACTACTGGAAAACTACCTGTTCCATGGC<br>CAACACTTGTCACTACTTTCGGTTATGGTGTTCAATGCTTTGCGAGATACCCAGATCATATGAA<br>ACAGCATGACTTTTTCAAGAGTGCCATGCCCGAAGGTTATGTACAGGAAAGAACTATATTTTTC<br>AAAGATGACGGGAACTACAAGACACGTGCTGAAGTCAAGTTTGAAGGTGATACCCTTGTTAAT<br>AGAATCGAGTTAAAAGGTATTGATTTTAAAGAAGATGGAAACATTCTTGGACACAAATTGGAA<br>TACAACTATAACTCACACAATGTATACATCATGGCAGACAAACAAAAGAATGGAATCAAAGTT<br>AACTTCAAAATTAGACACAACATTGAAGATGGAAGCGTTCAACTAGCAGACCATTATCAACAA<br>AATACTCCAATTGGCGATGGCCCTGTCCTTTTACCAGACAACCATTACCTGTCCACACAATCTGC<br>CCTTTCGAAAGATCCCAACGAAAAGAGAGACCACATGGTCCTTCTTGAGTTTGTAACAGCTGCT<br>GGGATTACACATGGCATGGATGAACTATACAAAAGGCCTGCAGCAAACGACGAAAACTACGC<br>TGCATCAGTTTAAACTAGTCTTGGACTCCTGTTGATAGATCCAGTAATGACCTCAGAACTCCAT<br>CTGGATTTGTTCAGAACGCTCGGTTGCCGCCGGGCGTTTTTTATTGGTGAGAATCCAGGGGTC<br>CCCAATAATTACGATTTACGTATTTAAATGAACCTTGACCGAACGCAGCGGTGGTAACGGCGC<br>AGTGGCGGTTTTCATGGCTTGTTATGACTGTTTTTTTGGGGTACAGTCTATGCCTCGGGCATCC<br>AAGCAGCAAGCGCGTTACGCCGTGGGTCGATGTTTGATGTTATGGAGCAGCAACGATGTTAC<br>GCAGCAGGGCAGTCGCCCTAAAACAAAGTTAAACATCATGAGGGAAGCGGTGATCGCCGAAG<br>TATCGACTCAACTATCAGAGGTAGTTGGCGTCATCGAGCGCCATCTCGAACCGACGTTGCTGG<br>CCGTACATTTGTACGGCTCCGCAGTGGATGGCGGCCTGAAGCCACACAGTGATATTGATTTGC<br>TGGTTACGGTGACCGTAAGGCTTGATGAAACAACGCGGCGAGCTTTGATCAACGACCTTTTGG<br>AAACTTCGGCTTCCCCTGGAGAGAGCGAGATTCTCCGCGCTGTAGAAGTCACCATTGTTGTGC<br>ACGACGACATCATTCCGTGGCGTTATCCAGCTAAGCGCGAACTGCAATTTGGAGAATGGCAGC<br>GCAATGACATTCTTGCAGGTATCTTCGAGCCAGCCACGATCGACATTGATCTGGCTATCTTGCT<br>GACAAAAGCAAGAGAACATAGCGTTGCCTTGGTAGGTCCAGCGGCGGAGGAACTCTTTGATC<br>CGGTTCCTGAACAGGATCTATTTGAGGCGCTAAATGAAACCTTAACGCTATGGAACTCGCCGC<br>CCGACTGGGCTGGCGATGAGCGAAATGTAGTGCTTACGTTGTCCCGCATTTGGTACAGCGCAG<br>TAACCGGCAAAATCGCGCCGAAGGATGTCGCTGCCGACTGGGCAATGGAGCGCCTGCCGGCC<br>CAGTATCAGCCCGTCATACTTGAAGCTAGACAGGCTTATCTTGGACAAGAAGAAGATCGCTTG<br>GCCTCGCGCGCAGATCAGTTGGAAGAATTTGTCCACTACGTGAAAGGCGAGATCACCAAGGT<br>AGTCGGCAAATAAGACAAGGTCCGCGCGTTGTCCTTTTCCGCTGCATAACCCTGCTTCGGGGT<br>CATTATAGCGATTTTTTCGGTATATCCATCCTTTTTCGCACGATATACAGGATTTTGCCAAAGGG<br>TTCGTGTAGACTTTCCTTGGTGTATCCAACGGCGTCAGCCGGGCAGGATAGGTGAAGTAGGCC<br>CACCCGCGAGCGGGTGTTCCTTCTTCACTGTCCCTTATTCGCACCTGGCGGTGCTCAACGGGAA<br>TCCTGCTCTGCGAGGCTGGCCGTAGGCCGGCCCTACCGGCGCGGCAGCGTTACCCGTGTCGGC<br>GGCTCCAACGGCTCGCCATCGTCCAGAAAACACGGCTCATCGGGCATCGGCAGGCGCTGCTG<br>CCCGCGCCGTTCCCATTCCTCCGTTTCGGTCAAGGCTGGCAGGTCTGGTTCCATGCCCGGAATG<br>CCGGGCTGGCTGGGCGGCTCCTCGCCGGGGCCGGTCGGTAGTTGCTGCTCGCCCGGATACAG<br>GGTCGGGATGCGGCGCAGGTCGCCATGCCCCAACAGCGATTCGTCCTGGTCGTCGTGATCAAC<br>CACCACGGCGGCACTGAACACCGACAGGCGCAACTGGTCGCGGGGCTGGCCCCACGCCACGC<br>GGTCATTGACCACGTAGGCCGACACGGTGCCGGGGCCGTTGAGCTTCACGACGGAGATCCAG<br>CGCTCGGCCACCAAGTCCTTGACTGCGTATTGGACCGTCCGCAAAGAACGTCCGATGAGCTTG<br>GAAAGTGTCTTCTGGCTGACCACCACGGCGTTCTGGTGGCCCATCTGCGCCACGAGGTGATGC

	AGCAGCATTGCCGCCGTGGGTTTCCTCGCAATAAGCCCGGCCCACGCCTCATGCGCTTTGCGTT<br>CCGTTTGCACCCAGTGACCGGGCTTGTTCTTGGCTTGAATGCCGATTTCTCTGGACTGCGTGGC<br>CATGCTTATCTCCATGCGGTAGGGGTGCCGCACGGTTGCGGCACCATGCGCAATCAGCTGCAA<br>CTTTTCGGCAGCGCGACAACAATTATGCGTTGCGTAAAAGTGGCAGTCAATTACAGATTTTCTT<br>TAACCTACGCAATGAGCTATTGCGGGGGGTGCCGCAATGAGCTGTTGCGTACCCCCCTTTTTTA<br>AGTTGTTGATTTTTAAGTCTTTCGCATTTCGCCCTATATCTAGTTCTTTGGTGCCCAAAGAAGGG<br>CACCCCTGCGGGGTTCCCCCACGCCTTCGGCGCGGCTCCCCCTCCGGCAAAAAGTGGCCCCTC<br>CGGGGCTTGTTGATCGACTGCGCGGCCTTCGGCCTTGCCCAAGGTGGCGCTGCCCCCTTGGAA<br>CCCCCGCACTCGCCGCCGTGAGGCTCGGGGGGCAGGCGGGCGGGCTTCGCCCTTCGACTGCC<br>CCCACTCGCATAGGCTTGGGTCGTTCCAGGCGCGTCAAGGCCAAGCCGCTGCGCGGTCGCTGC<br>GCGAGCCTTGACCCGCCTTCCACTTGGTGTCCAACCGGCAAGCGAAGCGCGCAGGCCGCAGG<br>CCGGAGGCTTTTCCCCAGAGAAAATTAAAAAAATTGATGGGGCAAGGCCGCAGGCCGCGCAG<br>TTGGAGCCGGTGGGTATGTGGTCGAAGGCTGGGTAGCCGGTGGGCAATCCCTGTGGTCAAGC<br>TCGTGGGCAGGCGCAGCCTGTCCATCAGCTTGTCCAGCAGGGTTGTCCACGGGCCGAGCGAA<br>GCGAGCCAGCCGGTGGCCGCTCGCGGCCATCGTCCACATATCCACGGGCTGGCAAGGGAGCG<br>CAGCGACCGCGCAGGGCGAAGCCCGGAGAGCAAGCCCGTAGGGGGGGCGCGCCCAGCTGTC<br>TAGGGCGGCGG
[$P_{ibpA/fxsA}$-gfp]	AATTCATCTGTTGATCGTGGGTGTTGGCCTGATGAGTTATAGCGATCCCTTGCTGAAAATAACA<br>TCATCATTACGTCGCACTGTGGCGGCTATCGCACTTTAACGTTTCGTGCTGCCCCCTCAGTCTAT<br>GCAATAGACCATAAACTGCAAAAAAAAGTCCGCTGATAAGGCTTGAAAAGTTCATTTCCAGAC<br>CCATTTTTACATCGTAGCCGATGAGGACGCGCCTGATGGGTGTTCTGGCTACCTGACCTGTCCA<br>TTGTGGAAGGTCTTACATTCTCGCTGATTTAGTCGACAAATTCTGGGATTACTACCAAAAATAG<br>TTGCGCAAACATCTTGAAATTTTGCTAATGACCACAATATAAGCTAAACGCGATTCGCAACCCA<br>TTCAGGTAGCCGGGGTTAACCGGCTGCTATTAAGAAGGAGATATACCC
[P_{secM}-gfp]	TTTTTTTTTGAATTTTATAAGAGCTATAAAAAACGGTGCGAACGCTGTTTTCTTAAGCACTTTTC<br>CGCACAACTTATCTTCATTCGTGCTGTGGACTGCAGGCTTTAATGATAAGATTTGTGCGCTAAA<br>TACGTTTGAATATGATCGGGATGGCAATAACGTGAGTGGAATACTGACGCGCTGGCGACAGT<br>TTGGTAAACGCTACTTCTGGCCGCATCTCTTATTAGGGATGGTTGCGGCGAGTTTAGGTTTGCC<br>TGCGCTCAGCAACGCCGCCGAACCAAACGCGCCCGCAAAAGCGACAACCCGCAACCACGAGC<br>CTTCAGCCAAAGTTAACTTTGGTCAATTGGCCTTGCTGGAAGCGAACACACGCCGCCCGAATTC<br>GAACTATTCCGTTGATTACTGGCATCAACATGCCATTCGCACGGTAATCCGTCATCTTTCTTTCG<br>CAATGGCACCGCAAACACTGCCCGTTGCTGAAGAATCTTTGCCTCTTCAGGCGCAACATCTTGC<br>ATTACTGGATACGCTCAGCGCGCTGCTGACCCAGGAAGGCACGCCGTCTGAAAAGGGTTATC<br>GCATTGATTATGCGCATTTTACCCCACAAGCAAAATTCAGCACGCCCGTCTGGATAAGCCAGG<br>CGCAAGGCATCCGTGCTGGCCCTCAACGCCTCACCTAACAACAATAAACCTTTACTTCATTTTAT<br>TAACTCCGCAACGCGGGGCGTTTGAGATTTTATT
[P_{cpxP}-gfp]	CGCTAATCCATGACTTTACGTTGTTTTACACCCCCTGACGCATGTTTGCAGCCTGAATCGTAAAC<br>TCTCTATCGTTGAATCGCGACAGAAAGATTTTGGGAGCAAATG
[P_{spy}-gfp]	ATCACCAGTCATCCGGTATAGTTCTTCATAATCTCTGCAAAATCATCGTGTTGTAATATTCTCTC<br>ATCACTCTCCATCAAATTTTCTTTTTTTCTCCATAATTGGCGCAAAAGTGTTTTTTACACTTTCATT<br>GTTTTACCGTTGCTCTGATTAATTGACGCTAAAGTCAGTAAAGTTAATCTCGTCAACACGGCAC<br>GCTACTTAAGAAAGCCGTAATAAATAACTGAAAGGAAGGATATAGAAT
[P_{psd}-gfp]	GGTAAAAACGCGTAAAAACTTTTCTGATACGGATGACTGACAATTAAGCTAACCGTCGTTAGA<br>ATCGTCCCCTTTTTTTCAGGATCCGGCATGTAGGCCGGATCAGGAACGACAAAACAATGGCCT<br>GGAGGCTACC
[P_{srkA}-gfp]	GTAAAAGCTTGTAAGCGGCGCCACCAAAATCATCGTGAAATGATATCCTTCGTCATTCGTAATG<br>TTTTCCGGATGATGGG
[P_{yebE}-gfp]	TTTTGTCTCGATATACATTTTCTGACATCCTCATCCGCTATGCTACAGGCACTAAAAATGGTTAT<br>TGAGGAGCAAAT
[P_{yccA}-gfp]	CGTAAAGATGGGTAAAACTTCTGGGTGCCCTTACGCATTATCATTATGCTGCTTAATTAATTAC<br>ATCTGTCATAGAGAGTGACTCA
[P_{yqjA}-gfp]	AAATTCCTGTCCGACAGGCGCTGCAATGCGCCTGTTTGTCTCGTCGGTAAGCGAAAAGTATAA<br>AGTGTCAGCCTGTGTAAATCCTCTCGCCACCCTCCCCTGCATTCAGCAAAATCAGACTCCACGG<br>ACATGTAATTTTGATAACGAACAACGTTGACCTTTGTTACAATTAGATTCAATTTGAATTTATGT<br>TTTTGAATGCTTTCTTATCTCACGATTTAACAGGGAATAGTTCAGGCTGTGTTGATGTATCAAAC

	CCGCAGAACATACCAAAACAGCAATAACATTGCGGTAGTGCATCTTTTAAAACCAGCGTGGCG<br>TTAACCGATTCACCAGGAATAATGA
[P_{ftnB}-gfp]	GTAAAAATATATAAACTTGATGATTTAAGCATTTTCTTATACCCGTTCAGACGTTATTCTTATTTC<br>AGATCATCGTCAGAATTGACTCCACGATCACATTTCGGACCGGCAGAAAGGAATTATTCTGCA<br>AACAGTAATTATGGTGTTTTGATTTATCTTGCACCTCTCCACTTCTGGATATAAGGATATTAGGT
[P_{idtC}-gfp]	ATTTACAAATTCTTTGCACTTCCCTGCACTATCCGACACTGTCACCATCCATAATTCAGGCTTATC<br>TGTTTATTACAATAACCTTATATTTATTATGGATTTTTG
[P_{degP}-gfp]	CTATGTCGAGGCTGTCAGTAAATTACCGTCAGATTCTCCTGAGTTTCCGCTATGGGAATATTAT<br>TACCGTTGCCGCCTGCTGCAGGATTATATCAGCGGTATGACCGACCTCTATGCGTGGGATGAA<br>TACCGACGTCTGATGGCCGTAGAACAATAACCAGGCTTTTGTAAAGACGAACAATAAATTTTTA<br>CCTTTTGCAGAAACTTTAGTTCGGAACTTCAGGCTATAAAACGAATCTGAAGAACACAGCAATT<br>TTGCGTTATCTGTTAATCGAGACTGAAATAC
pQC1	AACTGCAATTTGGAGAATGGCAGCGCAATGACATTCTTGCAGGTATCTTCGAGCCAGCCACGA<br>TCGACATTGATCTGGCTATCTTGCTGACAAAAGCAAGAGAACATAGCGTTGCCTTGGTAGGTC<br>CAGCGGCGGAGGAACTCTTTGATCCGGTTCCTGAACAGGATCTATTTGAGGCGCTAAATGAAA<br>CCTTAACGCTATGGAACTCGCCGCCCGACTGGGCTGGCGATGAGCGAAATGTAGTGCTTACGT<br>TGTCCCGCATTTGGTACAGCGCAGTAACCGGCAAAATCGCGCCGAAGGATGTCGCTGCCGACT<br>GGGCAATGGAGCGCCTGCCGGCCCAGTATCAGCCCGTCATACTTGAAGCTAGACAGGCTTATC<br>TTGGACAAGAAGAAGATCGCTTGGCCTCGCGCGCAGATCAGTTGGAAGAATTTGTCCACTACG<br>TGAAAGGCGAGATCACCAAGGTAGTCGGCAAATAAGACAAGGTCCGCGCGTTGTCCTTTTCCG<br>CTGCATAACCCTGCTTCGGGGTCATTATAGCGATTTTTTCGGTATATCCATCCTTTTTCGCACGA<br>TATACAGGATTTTGCCAAAGGGTTCGTGTAGACTTTCCTTGGTGTATCCAACGGCGTCAGCCG<br>GGCAGGATAGGTGAAGTAGGCCCACCCGCGAGCGGGTGTTCCTTCTTCACTGTCCCTTATTCG<br>CACCTGGCGGTGCTCAACGGGAATCCTGCTCTGCGAGGCTGGCCGTAGGCCGGCCCTACCGG<br>CGCGGCAGCGTTACCCGTGTCGGCGGCTCCAACGGCTCGCCATCGTCCAGAAAACACGGCTCA<br>TCGGGCATCGGCAGGCGCTGCTGCCCGCGCCGTTCCCATTCCTCCGTTTCGGTCAAGGCTGGC<br>AGGTCTGGTTCCATGCCCGGAATGCCGGGCTGGCTGGGCGGCTCCTCGCCGGGGCCGGTCGG<br>TAGTTGCTGCTCGCCCGGATACAGGGTCGGGATGCGGCGCAGGTCGCCATGCCCCAACAGCG<br>ATTCGTCCTGGTCGTCGTGATCAACCACCACGGCGGCACTGAACACCGACAGGCGCAACTGGT<br>CGCGGGGCTGGCCCCACGCCACGCGGTCATTGACCACGTAGGCCGACACGGTGCCGGGGCCG<br>TTGAGCTTCACGACGGAGATCCAGCGCTCGGCCACCAAGTCCTTGACTGCGTATTGGACCGTC<br>CGCAAAGAACGTCCGATGAGCTTGGAAAGTGTCTTCTGGCTGACCACCACGGCGTTCTGGTGG<br>CCCATCTGCGCCACGAGGTGATGCAGCAGCATTGCCGCCGTGGGTTTCCTCGCAATAAGCCCG<br>GCCCACGCCTCATGCGCTTTGCGTTCCGTTTGCACCCAGTGACCGGGCTTGTTCTTGGCTTGAA<br>TGCCGATTTCTCTGGACTGCGTGGCCATGCTTATCTCCATGCGGTAGGGGTGCCGCACGGTTG<br>CGGCACCATGCGCAATCAGCTGCAACTTTTCGGCAGCGCGACAACAATTATGCGTTGCGTAAA<br>AGTGGCAGTCAATTACAGATTTTCTTTAACCTACGCAATGAGCTATTGCGGGGGGTGCCGCAA<br>TGAGCTGTTGCGTACCCCCCTTTTTTAAGTTGTTGATTTTTAAGTCTTTCGCATTTCGCCCTATAT<br>CTAGTTCTTTGGTGCCCAAAGAAGGGCACCCCTGCGGGGTTCCCCCACGCCTTCGGCGCGGCT<br>CCCCCTCCGGCAAAAAGTGGCCCCTCCGGGGCTTGTTGATCGACTGCGCGGCCTTCGGCCTTG<br>CCCAAGGTGGCGCTGCCCCCTTGGAACCCCCGCACTCGCCGCCGTGAGGCTCGGGGGGCAGG<br>CGGGCGGGCTTCGCCCTTCGACTGCCCCCACTCGCATAGGCTTGGGTCGTTCCAGGCGCGTCA<br>AGGCCAAGCCGCTGCGCGGTCGCTGCGCGAGCCTTGACCCGCCTTCCACTTGGTGTCCAACCG<br>GCAAGCGAAGCGCGCAGGCCGCAGGCCGGAGGCTTTTCCCCAGAGAAAATTAAAAAAATTGA<br>TGGGGCAAGGCCGCAGGCCGCGCAGTTGGAGCCGGTGGGTATGTGGTCGAAGGCTAGGTAG<br>CCGGTGGGCAATCCCTGTGGTCAAGCTCGTGGGCAGGCGCAGCCTGTCCATCAGCTTGTCCAG<br>CAGGGTTGTCCACGGGCCGAGCGAAGCGAGCCAGCCGGTGGCCGCTCGCGGCCATCGTCCAC<br>ATATCCACGGGCTGGCAAGGGAGCGCAGCGACCGCGCAGGGCGAAGCCCGGAGAGCAAGCC<br>CGTAGGGGGGGCGCGCCCAGCTGTCTAGGGCGGCGGATTTGTCCTACTCAGGAGAGCGTTCA<br>CCGACAAACAACAGATAAAACGAAAGGCCCAGTCTTTCGACTGAGCCTTTCGTTTTATTTGATG<br>CCTTTAAGCTACTAAAGCGTAGTTTTCGTCGTTTGCTGCAGGCCTTTTGTATAGTTCATCCATGC<br>CTAGTGTAATCCCGGCGGCGGTCACGAACTCCAGCAGGACCATGTGATCGCGCTTCTCGTTGG<br>GGTCTTTGCTCAGCTTGGACTGGGTGCTCAGGTAGTGGTTGTCGGGCAGCAGCACGGGGCCG<br>TCGCCGATGGGGGTGTTCTGCTGGTAGTGGTCGGCGAGCTGCACGCTGCCGTCCTCGATGTTG<br>TGGCGGATCTTGAAGTTGGCCTTGATGCCGTTCTTCTGCTTGTCGGCGGTGATATAGACGTTGT<br>CGCTGATGGCGTTGTACTCCAGCTTGTGCCCCAGGATGTTGCCGTCCTCCTTGAAGTCGATGCC<br>CTTCAGCTCGATGCGGTTCACCAGGGTGTCGCCCTCGAACTTCACCTCGGCGCGGGTCTTGTA<br>GTTGCCGTCGTCCTTGAAGAAGATGGTGCGCTCCTGGACGTAGCCTTCGGGCATGGCGGACTT<br>GAAGAAGTCGTGCTGCTTCATGTGGTCGGGGTAGCGGGCGAAGCACTGCACGCCCCAGGTCA<br>GGGTGGTCACGAGGGTGGGCCAGGGCACGGGCAGCTTGCCGGTGGTGCAGATGAACTTCAG

	GGTCAGCTTGCCGTAGGTGGCATCGCCCTCGCCCTCGCCGGACACGCTGAACTTGTGGCCGTT<br>TACGTCGCCGTCCAGCTCGACCAGGATGGGCACCACCCCGGTGAAAAGTTCTTCTCCTTTACTC<br>ATAATAATCTCCCAAAATCTTTCTGTCGCGATTCAACGATAGAGAGTTTACGATTCAGGCTGCA<br>AACATGCGTCAGGGGGTGTAAAACAACGTAAAGTCATGGATTAGCGTTAACCTCAGACCAGG<br>ACCAAAACGAAAAAAGACGCTTTTCAGCGTCTCTTTTCTGGAATTTGGTACCGAGGACGGGAC<br>TTTTAAAATTCATCTGTTGATCGTGGGTGTTGGCCTGATGAGTTATAGCGATCCCTTGCTGAAA<br>ATAACATCATCATTACGTCGCACTGTGGCGGCTATCGCACTTTAACGTTTCGTGCTGCCCCCTCA<br>GTCTATGCAATAGACCATAAACTGCAAAAAAAAGTCCGCTGATAAGGCTTGAAAAGTTCATTT<br>CCAGACCCATTTTTACATCGTAGCCGATGAGGACGCGCCTGATGGGTGTTCTGGCTACCTGAC<br>CTGTCCATTGTGGAAGGTCTTACATTCTCGCTGATTTAGTCGACAAATTCTGGGATTACTACCA<br>AAAATAGTTGCGCAAACATCTTGAAATTTTGCTAATGACCACAATATAAGCTAAACGCGATTCG<br>CAACCCATTCAGGTAGCCGGGGTTAACCGGCTGCTATTAAGAAGGAGATATACCCATGCGTAA<br>AGGAGAAGAACTTTTCACTGGAGTTGTCCCAATTCTTGTTGAATTAGATGGTGATGTTAATGG<br>GCACAAATTTTCTGTCAGTGGAGAGGGTGAAGGTGATGCAACATACGGAAAACTTACCCTTAA<br>ATTTATTTGCACTACTGGAAAACTACCTGTTCCATGGCCAACACTTGTCACTACTTTCGGTTATG<br>GTGTTCAATGCTTTGCGAGATACCCAGATCATATGAAACAGCATGACTTTTTCAAGAGTGCCAT<br>GCCCGAAGGTTATGTACAGGAAAGAACTATATTTTTCAAAGATGACGGGAACTACAAGACACG<br>TGCTGAAGTCAAGTTTGAAGGTGATACCCTTGTTAATAGAATCGAGTTAAAAGGTATTGATTTT<br>AAAGAAGATGGAAACATTCTTGGACACAAATTGGAATACAACTATAACTCACACAATGTATAC<br>ATCATGGCAGACAAACAAAAGAATGGAATCAAAGTTAACTTCAAAATTAGACACAACATTGAA<br>GATGGAAGCGTTCAACTAGCAGACCATTATCAACAAAATACTCCAATTGGCGATGGCCCTGTC<br>CTTTTACCAGACAACCATTACCTGTCCACACAATCTGCCCTTTCGAAAGATCCCAACGAAAAGA<br>GAGACCACATGGTCCTTCTTGAGTTTGTAACAGCTGCTGGGATTACACATGGCATGGATGAAC<br>TATACAAAAGGCCTGCAGCAAACGACGAAAACTACGCTGCATCAGTTTAAACTAGTCTTGGAC<br>TCCTGTTGATAGATCCAGTAATGACCTCAGAACTCCATCTGGATTTGTTCAGAACGCTCGGTTG<br>CCGCCGGGCGTTTTTTATTGGTGAGAATCCAGGGGTCCCCAATAATTACGATTTACGTATTTAA<br>ATGAACCTTGACCGAACGCAGCGGTGGTAACGGCGCAGTGGCGGTTTTCATGGCTTGTTATGA<br>CTGTTTTTTTGGGGTACAGTCTATGCCTCGGGCATCCAAGCAGCAAGCGCGTTACGCCGTGGG<br>TCGATGTTTGATGTTATGGAGCAGCAACGATGTTACGCAGCAGGGCAGTCGCCCTAAAACAAA<br>GTTAAACATCATGAGGGAAGCGGTGATCGCCGAAGTATCGACTCAACTATCAGAGGTAGTTG<br>GCGTCATCGAGCGCCATCTCGAACCGACGTTGCTGGCCGTACATTTGTACGGCTCCGCAGTGG<br>ATGGCGGCCTGAAGCCACACAGTGATATTGATTTGCTGGTTACGGTGACCGTAAGGCTTGATG<br>AAACAACGCGGCGAGCTTTGATCAACGACCTTTTGGAAACTTCGGCTTCCCCTGGAGAGAGCG<br>AGATTCTCCGCGCTGTAGAAGTCACCATTGTTGTGCACGACGACATCATTCCGTGGCGTTATCC<br>AGCTAAGCGCG
pQC2	AACTGCAATTTGGAGAATGGCAGCGCAATGACATTCTTGCAGGTATCTTCGAGCCAGCCACGA<br>TCGACATTGATCTGGCTATCTTGCTGACAAAAGCAAGAGAACATAGCGTTGCCTTGGTAGGTC<br>CAGCGGCGGAGGAACTCTTTGATCCGGTTCCTGAACAGGATCTATTTGAGGCGCTAAATGAAA<br>CCTTAACGCTATGGAACTCGCCGCCCGACTGGGCTGGCGATGAGCGAAATGTAGTGCTTACGT<br>TGTCCCGCATTTGGTACAGCGCAGTAACCGGCAAAATCGCGCCGAAGGATGTCGCTGCCGACT<br>GGGCAATGGAGCGCCTGCCGGCCCAGTATCAGCCCGTCATACTTGAAGCTAGACAGGCTTATC<br>TTGGACAAGAAGAAGATCGCTTGGCCTCGCGCGCAGATCAGTTGGAAGAATTTGTCCACTACG<br>TGAAAGGCGAGATCACCAAGGTAGTCGGCAAATAAGACAAGGTCCGCGCGTTGTCCTTTTCCG<br>CTGCATAACCCTGCTTCGGGGTCATTATAGCGATTTTTTCGGTATATCCATCCTTTTTCGCACGA<br>TATACAGGATTTTGCCAAAGGGTTCGTGTAGACTTTCCTTGGTGTATCCAACGGCGTCAGCCG<br>GGCAGGATAGGTGAAGTAGGCCCACCCGCGAGCGGGTGTTCCTTCTTCACTGTCCCTTATTCG<br>CACCTGGCGGTGCTCAACGGGAATCCTGCTCTGCGAGGCTGGCCGTAGGCCGGCCCTACCGG<br>CGCGGCAGCGTTACCCGTGTCGGCGGCTCCAACGGCTCGCCATCGTCCAGAAAACACGGCTCA<br>TCGGGCATCGGCAGGCGCTGCTGCCCGCGCCGTTCCCATTCCTCCGTTTCGGTCAAGGCTGGC<br>AGGTCTGGTTCCATGCCCGGAATGCCGGGCTGGCTGGGCGGCTCCTCGCCGGGGCCGGTCGG<br>TAGTTGCTGCTCGCCCGGATACAGGGTCGGGATGCGGCGCAGGTCGCCATGCCCCAACAGCG<br>ATTCGTCCTGGTCGTCGTGATCAACCACCACGGCGGCACTGAACACCGACAGGCGCAACTGGT<br>CGCGGGGCTGGCCCCACGCCACGCGGTCATTGACCACGTAGGCCGACACGGTGCCGGGGCCG<br>TTGAGCTTCACGACGGAGATCCAGCGCTCGGCCACCAAGTCCTTGACTGCGTATTGGACCGTC<br>CGCAAAGAACGTCCGATGAGCTTGGAAAGTGTCTTCTGGCTGACCACCACGGCGTTCTGGTGG<br>CCCATCTGCGCCACGAGGTGATGCAGCAGCATTGCCGCCGTGGGTTTCCTCGCAATAAGCCCG<br>GCCCACGCCTCATGCGCTTTGCGTTCCGTTTGCACCCAGTGACCGGGCTTGTTCTTGGCTTGAA<br>TGCCGATTTCTCTGGACTGCGTGGCCATGCTTATCTCCATGCGGTAGGGGTGCCGCACGGTTG<br>CGGCACCATGCGCAATCAGCTGCAACTTTTCGGCAGCGCGACAACAATTATGCGTTGCGTAAA<br>AGTGGCAGTCAATTACAGATTTTCTTTAACCTACGCAATGAGCTATTGCGGGGGGTGCCGCAA<br>TGAGCTGTTGCGTACCCCCCTTTTTTAAGTTGTTGATTTTTAAGTCTTTCGCATTTCGCCCTATAT<br>CTAGTTCTTTGGTGCCCAAAGAAGGGCACCCCTGCGGGGTTCCCCCACGCCTTCGGCGCGGCT

	CCCCCTCCGGCAAAAAGTGGCCCCTCCGGGGCTTGTTGATCGACTGCGCGGCCTTCGGCCTTG<br>CCCAAGGTGGCGCTGCCCCCTTGGAACCCCCGCACTCGCCGCCGTGAGGCTCGGGGGGCAGG<br>CGGGCGGGCTTCGCCCTTCGACTGCCCCCACTCGCATAGGCTTGGGTCGTTCCAGGCGCGTCA<br>AGGCCAAGCCGCTGCGCGGTCGCTGCGCGAGCCTTGACCCGCCTTCCACTTGGTGTCCAACCG<br>GCAAGCGAAGCGCGCAGGCCGCAGGCCGGAGGCTTTTCCCCAGAGAAAATTAAAAAAATTGA<br>TGGGGCAAGGCCGCAGGCCGCGCAGTTGGAGCCGGTGGGTATGTGGTCGAAGGCTAGGTAG<br>CCGGTGGGCAATCCCTGTGGTCAAGCTCGTGGGCAGGCGCAGCCTGTCCATCAGCTTGTCCAG<br>CAGGGTTGTCCACGGGCCGAGCGAAGCGAGCCAGCCGGTGGCCGCTCGCGGCCATCGTCCAC<br>ATATCCACGGGCTGGCAAGGGAGCGCAGCGACCGCGCAGGGCGAAGCCCGGAGAGCAAGCC<br>CGTAGGGGGGGCGCGCCCAGCTGTCTAGGGCGGCGGATTTGTCCTACTCAGGAGAGCGTTCA<br>CCGACAAACAACAGATAAAACGAAAGGCCCAGTCTTTCGACTGAGCCTTTCGTTTTATTTGATG<br>CCTTTAAGCTACTAAAGCGTAGTTTTCGTCGTTTGCTGCAGGCCTTTTGTATAGTTCATCCATGC<br>CTAGTGTAATCCCGGCGGCGGTCACGAACTCCAGCAGGACCATGTGATCGCGCTTCTCGTTGG<br>GGTCTTTGCTCAGCTTGGACTGGGTGCTCAGGTAGTGGTTGTCGGGCAGCAGCACGGGGCCG<br>TCGCCGATGGGGGTGTTCTGCTGGTAGTGGTCGGCGAGCTGCACGCTGCCGTCCTCGATGTTG<br>TGGCGGATCTTGAAGTTGGCCTTGATGCCGTTCTTCTGCTTGTCGGCGGTGATATAGACGTTGT<br>CGCTGATGGCGTTGTACTCCAGCTTGTGCCCCAGGATGTTGCCGTCCTCCTTGAAGTCGATGCC<br>CTTCAGCTCGATGCGGTTCACCAGGGTGTCGCCCTCGAACTTCACCTCGGCGCGGGTCTTGTA<br>GTTGCCGTCGTCCTTGAAGAAGATGGTGCGCTCCTGGACGTAGCCTTCGGGCATGGCGGACTT<br>GAAGAAGTCGTGCTGCTTCATGTGGTCGGGGTAGCGGGCGAAGCACTGCACGCCCCAGGTCA<br>GGGTGGTCACGAGGGTGGGCCAGGGCACGGGCAGCTTGCCGGTGGTGCAGATGAACTTCAG<br>GGTCAGCTTGCCGTAGGTGGCATCGCCCTCGCCCTCGCCGGACACGCTGAACTTGTGGCCGTT<br>TACGTCGCCGTCCAGCTCGACCAGGATGGGCACCACCCCGGTGAAAAGTTCTTCTCCTTTACTC<br>ATAATAATAATAAAATCTCAAACGCCCCGCGTTGCGGAGTTAATAAAATGAAGTAAAGGTTTA<br>TTGTTGTTAGGTGAGGCGTTGAGGGCCAGCACGGATGCCTTGCGCCTGGCTTATCCAGACGG<br>GCGTGCTGAATTTTGCTTGTGGGGTAAAATGCGCATAATCAATGCGATAACCCTTTTCAGACG<br>GCGTGCCTTCCTGGGTCAGCAGCGCGCTGAGCGTATCCAGTAATGCAAGATGTTGCGCCTGAA<br>GAGGCAAAGATTCTTCAGCAACGGGCAGTGTTTGCGGTGCCATTGCGAAAGAAAGATGACGG<br>ATTACCGTGCGAATGGCATGTTGATGCCAGTAATCAACGGAATAGTTCGAATTCGGGCGGCGT<br>GTGTTCGCTTCCAGCAAGGCCAATTGACCAAAGTTAACTTTGGCTGAAGGCTCGTGGTTGCGG<br>GTTGTCGCTTTTGCGGGCGCGTTTGGTTCGGCGGCGTTGCTGAGCGCAGGCAAACCTAAACTC<br>GCCGCAACCATCCCTAATAAGAGATGCGGCCAGAAGTAGCGTTTACCAAACTGTCGCCAGCGC<br>GTCAGTATTCCACTCACGTTATTGCCATCCCGATCATATTCAAACGTATTTAGCGCACAAATCTT<br>ATCATTAAAGCCTGCAGTCCACAGCACGAATGAAGATAAGTTGTGCGGAAAAGTGCTTAAGAA<br>AACAGCGTTCGCACCGTTTTTTATAGCTCTTATAAAATTCAAAAAAAAATTAACCTCAGACCAG<br>GACCAAAACGAAAAAAGACGCTTTTCAGCGTCTCTTTTCTGGAATTTGGTACCGAGGACGGGA<br>CTTTTAAAATTCATCTGTTGATCGTGGGTGTTGGCCTGATGAGTTATAGCGATCCCTTGCTGAA<br>AATAACATCATCATTACGTCGCACTGTGGCGGCTATCGCACTTTAACGTTTCGTGCTGCCCCCTC<br>AGTCTATGCAATAGACCATAAACTGCAAAAAAAAGTCCGCTGATAAGGCTTGAAAAGTTCATT<br>TCCAGACCCATTTTTACATCGTAGCCGATGAGGACGCGCCTGATGGGTGTTCTGGCTACCTGAC<br>CTGTCCATTGTGGAAGGTCTTACATTCTCGCTGATTTAGTCGACAAATTCTGGGATTACTACCA<br>AAAATAGTTGCGCAAACATCTTGAAATTTTGCTAATGACCACAATATAAGCTAAACGCGATTCG<br>CAACCCATTCAGGTAGCCGGGGTTAACCGGCTGCTATTAAGAAGGAGATATACCCATGCGTAA<br>AGGAGAAGAACTTTTCACTGGAGTTGTCCCAATTCTTGTTGAATTAGATGGTGATGTTAATGG<br>GCACAAATTTTCTGTCAGTGGAGAGGGTGAAGGTGATGCAACATACGGAAAACTTACCCTTAA<br>ATTTATTTGCACTACTGGAAAACTACCTGTTCCATGGCCAACACTTGTCACTACTTTCGGTTATG<br>GTGTTCAATGCTTTGCGAGATACCCAGATCATATGAAACAGCATGACTTTTTCAAGAGTGCCAT<br>GCCCGAAGGTTATGTACAGGAAAGAACTATATTTTTCAAAGATGACGGGAACTACAAGACACG<br>TGCTGAAGTCAAGTTTGAAGGTGATACCCTTGTTAATAGAATCGAGTTAAAAGGTATTGATTTT<br>AAAGAAGATGGAAACATTCTTGGACACAAATTGGAATACAACTATAACTCACACAATGTATAC<br>ATCATGGCAGACAAACAAAAGAATGGAATCAAAGTTAACTTCAAAATTAGACACAACATTGAA<br>GATGGAAGCGTTCAACTAGCAGACCATTATCAACAAAATACTCCAATTGGCGATGGCCCTGTC<br>CTTTTACCAGACAACCATTACCTGTCCACACAATCTGCCCTTTCGAAAGATCCCAACGAAAAGA<br>GAGACCACATGGTCCTTCTTGAGTTTGTAACAGCTGCTGGGATTACACATGGCATGGATGAAC<br>TATACAAAAGGCCTGCAGCAAACGACGAAAACTACGCTGCATCAGTTTAAACTAGTCTTGGAC<br>TCCTGTTGATAGATCCAGTAATGACCTCAGAACTCCATCTGGATTTGTTCAGAACGCTCGGTTG<br>CCGCCGGGCGTTTTTTATTGGTGAGAATCCAGGGGTCCCCAATAATTACGATTTACGTATTTAA<br>ATGAACCTTGACCGAACGCAGCGGTGGTAACGGCGCAGTGGCGGTTTTCATGGCTTGTTATGA<br>CTGTTTTTTTGGGGTACAGTCTATGCCTCGGGCATCCAAGCAGCAAGCGCGTTACGCCGTGGG<br>TCGATGTTTGATGTTATGGAGCAGCAACGATGTTACGCAGCAGGGCAGTCGCCCTAAAACAAA<br>GTTAAACATCATGAGGGAAGCGGTGATCGCCGAAGTATCGACTCAACTATCAGAGGTAGTTG<br>GCGTCATCGAGCGCCATCTCGAACCGACGTTGCTGGCCGTACATTTGTACGGCTCCGCAGTGG<br>ATGGCGGCCTGAAGCCACACAGTGATATTGATTTGCTGGTTACGGTGACCGTAAGGCTTGATG

	AAACAACGCGGCGAGCTTTGATCAACGACCTTTTGGAAACTTCGGCTTCCCCTGGAGAGAGCG<br>AGATTCTCCGCGCTGTAGAAGTCACCATTGTTGTGCACGACGACATCATTCCGTGGCGTTATCC<br>AGCTAAGCGCG
pQC3	AACTGCAATTTGGAGAATGGCAGCGCAATGACATTCTTGCAGGTATCTTCGAGCCAGCCACGA<br>TCGACATTGATCTGGCTATCTTGCTGACAAAAGCAAGAGAACATAGCGTTGCCTTGGTAGGTC<br>CAGCGGCGGAGGAACTCTTTGATCCGGTTCCTGAACAGGATCTATTTGAGGCGCTAAATGAAA<br>CCTTAACGCTATGGAACTCGCCGCCCGACTGGGCTGGCGATGAGCGAAATGTAGTGCTTACGT<br>TGTCCCGCATTTGGTACAGCGCAGTAACCGGCAAAATCGCGCCGAAGGATGTCGCTGCCGACT<br>GGGCAATGGAGCGCCTGCCGGCCCAGTATCAGCCCGTCATACTTGAAGCTAGACAGGCTTATC<br>TTGGACAAGAAGAAGATCGCTTGGCCTCGCGCGCAGATCAGTTGGAAGAATTTGTCCACTACG<br>TGAAAGGCGAGATCACCAAGGTAGTCGGCAAATAAGACAAGGTCCGCGCGTTGTCCTTTTCCG<br>CTGCATAACCCTGCTTCGGGGTCATTATAGCGATTTTTTCGGTATATCCATCCTTTTTCGCACGA<br>TATACAGGATTTTGCCAAAGGGTTCGTGTAGACTTTCCTTGGTGTATCCAACGGCGTCAGCCG<br>GGCAGGATAGGTGAAGTAGGCCCACCCGCGAGCGGGTGTTCCTTCTTCACTGTCCCTTATTCG<br>CACCTGGCGGTGCTCAACGGGAATCCTGCTCTGCGAGGCTGGCCGTAGGCCGGCCCTACCGG<br>CGCGGCAGCGTTACCCGTGTCGGCGGCTCCAACGGCTCGCCATCGTCCAGAAAACACGGCTCA<br>TCGGGCATCGGCAGGCGCTGCTGCCCGCGCCGTTCCCATTCCTCCGTTTCGGTCAAGGCTGGC<br>AGGTCTGGTTCCATGCCCGGAATGCCGGGCTGGCTGGGCGGCTCCTCGCCGGGGCCGGTCGG<br>TAGTTGCTGCTCGCCCGGATACAGGGTCGGGATGCGGCGCAGGTCGCCATGCCCCAACAGCG<br>ATTCGTCCTGGTCGTCGTGATCAACCACCACGGCGGCACTGAACACCGACAGGCGCAACTGGT<br>CGCGGGGCTGGCCCCACGCCACGCGGTCATTGACCACGTAGGCCGACACGGTGCCGGGGCCG<br>TTGAGCTTCACGACGGAGATCCAGCGCTCGGCCACCAAGTCCTTGACTGCGTATTGGACCGTC<br>CGCAAAGAACGTCCGATGAGCTTGGAAAGTGTCTTCTGGCTGACCACCACGGCGTTCTGGTGG<br>CCCATCTGCGCCACGAGGTGATGCAGCAGCATTGCCGCCGTGGGTTTCCTCGCAATAAGCCCG<br>GCCCACGCCTCATGCGCTTTGCGTTCCGTTTGCACCCAGTGACCGGGCTTGTTCTTGGCTTGAA<br>TGCCGATTTCTCTGGACTGCGTGGCCATGCTTATCTCCATGCGGTAGGGGTGCCGCACGGTTG<br>CGGCACCATGCGCAATCAGCTGCAACTTTTCGGCAGCGCGACAACAATTATGCGTTGCGTAAA<br>AGTGGCAGTCAATTACAGATTTTCTTTAACCTACGCAATGAGCTATTGCGGGGGGTGCCGCAA<br>TGAGCTGTTGCGTACCCCCCTTTTTTAAGTTGTTGATTTTTAAGTCTTTCGCATTTCGCCCTATAT<br>CTAGTTCTTTGGTGCCCAAAGAAGGGCACCCCTGCGGGGTTCCCCCACGCCTTCGGCGCGGCT<br>CCCCCTCCGGCAAAAAGTGGCCCCTCCGGGGCTTGTTGATCGACTGCGCGGCCTTCGGCCTTG<br>CCCAAGGTGGCGCTGCCCCCTTGGAACCCCCGCACTCGCCGCCGTGAGGCTCGGGGGGCAGG<br>CGGGCGGGCTTCGCCCTTCGACTGCCCCCACTCGCATAGGCTTGGGTCGTTCCAGGCGCGTCA<br>AGGCCAAGCCGCTGCGCGGTCGCTGCGCGAGCCTTGACCCGCCTTCCACTTGGTGTCCAACCG<br>GCAAGCGAAGCGCGCAGGCCGCAGGCCGGAGGCTTTTCCCCAGAGAAAATTAAAAAAATTGA<br>TGGGGCAAGGCCGCAGGCCGCGCAGTTGGAGCCGGTGGGTATGTGGTCGAAGGCTAGGTAG<br>CCGGTGGGCAATCCCTGTGGTCAAGCTCGTGGGCAGGCGCAGCCTGTCCATCAGCTTGTCCAG<br>CAGGGTTGTCCACGGGCCGAGCGAAGCGAGCCAGCCGGTGGCCGCTCGCGGCCATCGTCCAC<br>ATATCCACGGGCTGGCAAGGGAGCGCAGCGACCGCGCAGGGCGAAGCCCGGAGAGCAAGCC<br>CGTAGGGGGGGCGCGCCCAGCTGTCTAGGGCGGCGGATTTGTCCTACTCAGGAGAGCGTTCA<br>CCGACAAACAACAGATAAAACGAAAGGCCCAGTCTTTCGACTGAGCCTTTCGTTTTATTTGATG<br>CCTTTAAGCTACTAAAGCGTAGTTTTCGTCGTTTGCTGCAGGCCTTTTGTATAGTTCATCCATGC<br>CTAGTGTAATCCCGGCGGCGGTCACGAACTCCAGCAGGACCATGTGATCGCGCTTCTCGTTGG<br>GGTCTTTGCTCAGCTTGGACTGGGTGCTCAGGTAGTGGTTGTCGGGCAGCAGCACGGGGCCG<br>TCGCCGATGGGGGTGTTCTGCTGGTAGTGGTCGGCGAGCTGCACGCTGCCGTCCTCGATGTTG<br>TGGCGGATCTTGAAGTTGGCCTTGATGCCGTTCTTCTGCTTGTCGGCGGTGATATAGACGTTGT<br>CGCTGATGGCGTTGTACTCCAGCTTGTGCCCCAGGATGTTGCCGTCCTCCTTGAAGTCGATGCC<br>CTTCAGCTCGATGCGGTTCACCAGGGTGTCGCCCTCGAACTTCACCTCGGCGCGGGTCTTGTA<br>GTTGCCGTCGTCCTTGAAGAAGATGGTGCGCTCCTGGACGTAGCCTTCGGGCATGGCGGACTT<br>GAAGAAGTCGTGCTGCTTCATGTGGTCGGGGTAGCGGGCGAAGCACTGCACGCCCCAGGTCA<br>GGGTGGTCACGAGGGTGGGCCAGGGCACGGGCAGCTTGCCGGTGGTGCAGATGAACTTCAG<br>GGTCAGCTTGCCGTAGGTGGCATCGCCCTCGCCCTCGCCGGACACGCTGAACTTGTGGCCGTT<br>TACGTCGCCGTCCAGCTCGACCAGGATGGGCACCACCCCGGTGAAAAGTTCTTCTCCTTTACTC<br>ATAATAATCTCCCAAAATCTTTCTGTCGCGATTCAACGATAGAGAGTTTACGATTCAGGCTGCA<br>AACATGCGTCAGGGGGTGTAAAACAACGTAAAGTCATGGATTAGCGTTAACCTCAGACCAGG<br>ACCAAAACGAAAAAAGACGCTTTTCAGCGTCTCTTTTCTGGAATTTGGTACCGAGGACGGGAC<br>TTTTAATTTTTTTTTGAATTTTATAAGAGCTATAAAAAACGGTGCGAACGCTGTTTTCTTAAGCA<br>CTTTTCCGCACAACTTATCTTCATTCGTGCTGTGGACTGCAGGCTTTAATGATAAGATTTGTGCG<br>CTAAATACGTTTGAATATGATCGGGATGGCAATAACGTGAGTGGAATACTGACGCGCTGGCG<br>ACAGTTTGGTAAACGCTACTTCTGGCCGCATCTCTTATTAGGGATGGTTGCGGCGAGTTTAGG<br>TTTGCCTGCGCTCAGCAACGCCGCCGAACCAAACGCGCCCGCAAAAGCGACAACCCGCAACCA<br>CGAGCCTTCAGCCAAAGTTAACTTTGGTCAATTGGCCTTGCTGGAAGCGAACACACGCCGCCC

	GAATTCGAACTATTCCGTTGATTACTGGCATCAACATGCCATTCGCACGGTAATCCGTCATCTTT<br>CTTTCGCAATGGCACCGCAAACACTGCCCGTTGCTGAAGAATCTTTGCCTCTTCAGGCGCAACA<br>TCTTGCATTACTGGATACGCTCAGCGCGCTGCTGACCCAGGAAGGCACGCCGTCTGAAAAGGG<br>TTATCGCATTGATTATGCGCATTTTACCCCACAAGCAAAATTCAGCACGCCCGTCTGGATAAGC<br>CAGGCGCAAGGCATCCGTGCTGGCCCTCAACGCCTCACCTAACAACAATAAACCTTTACTTCAT<br>TTTATTAACTCCGCAACGCGGGGCGTTTGAGATTTTATTATGCGTAAAGGAGAAGAACTTTTCA<br>CTGGAGTTGTCCCAATTCTTGTTGAATTAGATGGTGATGTTAATGGGCACAAATTTTCTGTCAG<br>TGGAGAGGGTGAAGGTGATGCAACATACGGAAAACTTACCCTTAAATTTATTTGCACTACTGG<br>AAAACTACCTGTTCCATGGCCAACACTTGTCACTACTTTCGGTTATGGTGTTCAATGCTTTGCGA<br>GATACCCAGATCATATGAAACAGCATGACTTTTTCAAGAGTGCCATGCCCGAAGGTTATGTAC<br>AGGAAAGAACTATATTTTTCAAAGATGACGGGAACTACAAGACACGTGCTGAAGTCAAGTTTG<br>AAGGTGATACCCTTGTTAATAGAATCGAGTTAAAAGGTATTGATTTTAAAGAAGATGGAAACA<br>TTCTTGGACACAAATTGGAATACAACTATAACTCACACAATGTATACATCATGGCAGACAAACA<br>AAAGAATGGAATCAAAGTTAACTTCAAAATTAGACACAACATTGAAGATGGAAGCGTTCAACT<br>AGCAGACCATTATCAACAAAATACTCCAATTGGCGATGGCCCTGTCCTTTTACCAGACAACCAT<br>TACCTGTCCACACAATCTGCCCTTTCGAAAGATCCCAACGAAAAGAGAGACCACATGGTCCTTC<br>TTGAGTTTGTAACAGCTGCTGGGATTACACATGGCATGGATGAACTATACAAAAGGCCTGCAG<br>CAAACGACGAAAACTACGCTGCATCAGTTTAAACTAGTCTTGGACTCCTGTTGATAGATCCAGT<br>AATGACCTCAGAACTCCATCTGGATTTGTTCAGAACGCTCGGTTGCCGCCGGGCGTTTTTTATT<br>GGTGAGAATCCAGGGGTCCCCAATAATTACGATTTACGTATTTAAATGAACCTTGACCGAACG<br>CAGCGGTGGTAACGGCGCAGTGGCGGTTTTCATGGCTTGTTATGACTGTTTTTTTGGGGTACA<br>GTCTATGCCTCGGGCATCCAAGCAGCAAGCGCGTTACGCCGTGGGTCGATGTTTGATGTTATG<br>GAGCAGCAACGATGTTACGCAGCAGGGCAGTCGCCCTAAAACAAAGTTAAACATCATGAGGG<br>AAGCGGTGATCGCCGAAGTATCGACTCAACTATCAGAGGTAGTTGGCGTCATCGAGCGCCATC<br>TCGAACCGACGTTGCTGGCCGTACATTTGTACGGCTCCGCAGTGGATGGCGGCCTGAAGCCAC<br>ACAGTGATATTGATTTGCTGGTTACGGTGACCGTAAGGCTTGATGAAACAACGCGGCGAGCTT<br>TGATCAACGACCTTTTGGAAACTTCGGCTTCCCCTGGAGAGAGCGAGATTCTCCGCGCTGTAG<br>AAGTCACCATTGTTGTGCACGACGACATCATTCCGTGGCGTTATCCAGCTAAGCGCG

Table S3. Details on the construction of the biosensors.

**Biosensor**	**Construction notes**	**Primers used**
[P_{ibpA}-gfp]	Described in [1].	
[$P_{ibpA/fxsA}$-gfp]	[$P_{ibpA/fxsA}$-gfp] was inspired by the work of Kraft et al [2] and Englund et al [3]. It was constructed in four steps; (1) the [P_{ibpA}-gfp] backbone was amplified by PCR using the MM20 / MM23 primers. (2) P_{fxsA} was amplified from the *E. coli* MC1061 genome using the MM24 / MM26 primers. MM26 contains an overhang to add the T7 SD sequence. (3) PCR products were *DpnI* treated and gel-purified, then ligated using the *in vivo* DNA assembly method in the *E. coli* MC1061 strain [4].	MM20:<br>ATGCGTAAAGGAGAAGAACTTTTC<br><br>MM23:<br>GAATTTGTCGACTAAATCAGCGAGAATGTAAGACC<br><br>MM24:<br>AGTCGACAAATTCTGGGATTACTACCAAAAATAGTTGC<br><br>MM26:<br>AGTTCTTCTCCTTTACGCATGGGTATATCTCCTTCTTAAT AGCAGCCGGTTAACCC
[P_{secM}-gfp]	[P_{secM}-gfp] was constructed in three steps; (1) the [P_{ibpA}-gfp] backbone was amplified by PCR using the MB1 / MB2 primers. (2) P_{secM}-secM was amplified from the *E. coli* MC1061 genome using the MB3 / MB4 primers. (3) PCR products were *DpnI* treated and gel-purified, then ligated using the *in vivo* DNA assembly method in the *E. coli* MC1061 strain [4].	MB1:<br>GAGATTTTATTATGCGTAAAGGAGAAGAACTTTTCAC<br><br>MB2:<br>CTCTTATAAAATTCAAAAAAAAAAGGCATCAAATAAAAC GAAAGGCTC<br><br>MB3:<br>TTTTTGAATTTTATAAGAGCTATAAAAAACGGTGCGAAC GCTGTTTTC<br><br>MB4:<br>CTCCTTTACGCATAATAAAATCTCAAACGCCCCGCG
[P_{cpxP}-gfp]	Described in [5].	
[P_{spy}-gfp]	[P_{spy}-gfp] was constructed in three steps; (1) the [P_{ibpA}-gfp] backbone was amplified by PCR using the JC65/ JC85 primers. (2) P_{spy} was amplified from the *E. coli* MC1061 genome using the JC139 / JC140 primers. (3) PCR products were *DpnI* treated and gel-purified, then ligated using the *in vivo* DNA assembly method in the *E. coli* MC1061 strain [4].	JC65:<br>ATGCGTAAAGGAGAAGAACTTTTCAC<br><br>JC85:<br>AGGCATCAAATAAAACGAAAGGCTCAGTCG<br><br>JC139:<br>CTTCTCCTTTACGCATATTCTATATCCTTCCTTTCAGTTAT TTATTAC<br><br>JC140:<br>CGTTTTATTTGATGCCTATCACCAGTCATCCGGTATAGTT
pQC1	pQC1 was constructed in the following steps. (1) P_{fxsA} was added to the [P_{ibpA}-gfp] promoter on pQC (see [4]) as described under [$P_{ibpA/fxsA}$-gfp] above. (2) The pQC backbone containing [$P_{ibpA/fxsA}$-gfp] was amplified using the MM19 / JC80 primers. (3) $mCerulean_{LVA}$ was amplified from pBAD:$mCerulean_{LVA}$ (see below) using the MM180 / MM181 primers. (4) PCR products were *DpnI* treated and gel-purified, then ligated using the *in vivo* DNA assembly method in the *E. coli* MC1061 strain [4]	MM19:<br>CAAATAAAACGAAAGGCTCAGTCG<br><br>JC80:<br>CTCCCAAAATCTTTCTGTCGCGATTC<br><br>MM180:<br>GAATCGCGACAGAAAGATTTTGGGAGATTATTATGAGT AAAGGAGAAGAACTTTTCACCG<br><br>MM181:<br>CGACTGAGCCTTTCGTTTTATTTGATGCCTTTAAGCTACT AAAGCGTAGTTTTCGTCG
pQC2	pQC2 was constructed in the following steps. (1) The pQC1 backbone was amplified using the MM185 / MM186 primers. (2) P_{secM}-secM was amplified from	MM183:<br>CGTTTTGGTCCTGGTCTGAGGTTAATTTTTTTTTGAATTT TATAAGAGCTATAAAAAACGGTGCGAACGC

	[P_{secM}-gfp] using the MM183 / MM184 primers. (3) PCR products were *DpnI* treated and gel-purified, then ligated using the *in vivo* DNA assembly method in the *E. coli* MC1061 strain [4]	MM184:<br>CCGGTGAAAAGTTCTTCTCCTTTACTCATAATAATAATAAAATCTCAAACGCCCCGCG<br><br>MM185:<br>TTAACCTCAGACCAGGACCAAAACG<br><br>MM186:<br>ATGAGTAAAGGAGAAGAACTTTTCACCG
pQC3	pQC3 was constructed in the following steps. (1) The pQC1 backbone was amplified using the MM20 / MM187 primers. 2) P_{secM}-secM was amplified from [P_{secM}-gfp] using the MM188 / MM189 primers. (3) PCR products were *DpnI* treated and gel-purified, then ligated using the *in vivo* DNA assembly method in the *E. coli* MC1061 strain [4]	MM20:<br>ATGCGTAAAGGAGAAGAACTTTTC<br><br>MM187:<br>TTAAAAGTCCCGTCCTCGGTAC<br><br>MM188:<br>GGAATTTGGTACCGAGGACGGGACTTTTAATTTTTTTTTGAATTTTATAAGAGCTATAAAAAACGGTGCGAACGC<br><br>MM189:<br>CAACTCCAGTGA AAAGTTCTTCTCCTTTACGCATAATAAAATCTCAAACGCCCCGCG

Table S4. Sequences of the expression plasmids used to evaluate the biosensors. *The coding sequence that was expressed is marked in blue, the coding sequence for mCherry in red and the coding sequence for mScarlet-I3 in purple. The coding sequence for the translational coupling device is underlined.*

**Expression plasmid**	**Linear sequence**
pBAD:Mth1-TCD-mCherry	AAGAAACCAATTGTCCATATTGCATCAGACATTGCCGTCACTGCGTCTTTTACTGGCTCTTCTCGCTAAC<br>CAAACCGGTAACCCCGCTTATTAAAAGCATTCTGTAACAAAGCGGGACCAAAGCCATGACAAAAACG<br>CGTAACAAAAGTGTCTATAATCACGGCAGAAAAGTCCACATTGATTATTTGCACGGCGTCACACTTTG<br>CTATGCCATAGCATTTTTATCCATAAGATTAGCGGATCCTACCTGACGCTTTTTATCGCAACTCTCTACT<br>GTTTCTCCATACCCGTTTTTTTGGGCTAGAAATAATTTTGTTTAACTTTAAGAAGGAGATATACATATG<br>GGTGCAAGCCGTCTGTATACCCTGGTTCTGGTGCTGCAGCCGCAACGTGTTCTGCTGGGTATGAAAAA<br>ACGTGGTTTTGGTGCAGGTCGTTGGAATGGTTTTGGTGGTAAAGTTCAGGAAGGCGAAACCATTGAA<br>GATGGTGCACGTCGTGAACTGCAGGAAGAAAGCGGTCTGACCGTTGATGCACTGCATAAAGTTGGCC<br>AGATTGTGTTTGAATTTGTGGGTGAACCGGAACTGATGGATGTTCATGTGTTTTGCACCGATAGCATT<br>CAGGGTACACCGGTTGAATCTGATGAAATGCGTCCGTGTTGGTTTCAGCTGGATCAGATTCCGTTTAA<br>AGATATGTGGCCTGATGATAGCTATTGGTTTCCGCTGCTGCTGCAGAAAAAGAAATTCCATGGCTACT<br>TCAAATTTCAGGGCCAGGATACCATTCTGGATTATACCCTGCGTGAAGTTGATACCGTGACTAGTCATC<br>ATCACCACCATCATTAGGATGGTGGTGATGATAATGATGAGCAAGGGCGAGGAGGATAACATGGCC<br>ATCATCAAGGAGTTCATGCGCTTCAAGGTGCACATGGAGGGCTCCGTGAACGGCCACGAGTTCGAGA<br>TCGAGGGCGAGGGCGAGGGCCGCCCCTACGAGGGCACCCAGACCGCCAAGCTGAAGGTGACCAAG<br>GGTGGCCCCCTGCCCTTCGCCTGGGACATCCTGTCCCCTCAGTTCATGTACGGCTCCAAGGCCTACGT<br>GAAGCACCCCGCCGACATCCCCGACTACTTGAAGCTGTCCTTCCCCGAGGGCTTCAAGTGGGAGCGC<br>GTGATGAACTTCGAGGACGGCGGCGTGGTGACCGTGACCCAGGACTCCTCCCTGCAGGACGGCGAG<br>TTCATCTACAAGGTGAAGCTGCGCGGCACCAACTTCCCCTCCGACGGCCCCGTAATGCAGAAGAAGAC<br>CATGGGCTGGGAGGCCTCCTCCGAGCGGATGTACCCCGAGGACGGCGCCCTGAAGGGCGAGATCAA<br>GCAGAGGCTGAAGCTGAAGGACGGCGGCCACTACGACGCTGAGGTCAAGACCACCTACAAGGCCAA<br>GAAGCCCGTGCAGCTGCCCGGCGCCTACAACGTCAACATCAAGTTGGACATCACCTCCCACAACGAG<br>GACTACACCATCGTGGAACAGTACGAACGCGCCGAGGGCCGCCACTCCACCGGCGGCATGGACGAG<br>CTGTACAAGTGAAAGCTTACGGTCTCCAGCTTGGCTGTTTTGGCGGATGAGAGAAGATTTTCAGCCTG<br>ATACAGATTAAATCAGAACGCAGAAGCGGTCTGATAAAACAGAATTTGCCTGGCGGCAGTAGCGCGG<br>TGGTCCCACCTGACCCCATGCCGAACTCAGAAGTGAAACGCCGTAGCGCCGATGGTAGTGTGGGGTC<br>TCCCCATGCGAGAGTAGGGAACTGCCAGGCATCAAATAAAACGAAAGGCTCAGTCGAAAGACTGGG<br>CCTTTCGTTTTATCTGTTGTTTGTCGGTGAACGCTCTCCTGAGTAGGACAAATCCGCCGGGAGCGGATT<br>TGAACGTTGCGAAGCAACGGCCCGGAGGGTGGCGGGCAGGACGCCCGCCATAAACTGCCAGGCATC<br>AAATTAAGCAGAAGGCCATCCTGACGGATGGCCTTTTTGCGTTTCTACAAACTCTCATGAACAATAAA<br>ACTGTCTGCTTACATAAACAGTAATACAAGGGGTGTTATGAGCCATATTCAACGGGAAACGTCTTGCT<br>CTAGGCCGCGATTAAATTCCAACATGGATGCTGATTTATATGGGTATAAATGGGCTCGCGATAATGTC<br>GGGCAATCAGGTGCGACAATCTATCGATTGTATGGGAAGCCCGATGCGCCAGAGTTGTTTCTGAAAC<br>ATGGCAAAGGTAGCGTTGCCAATGATGTTACAGATGAGATGGTCAGACTAAACTGGCTGACGGAATT<br>TATGCCTCTTCCGACCATCAAGCATTTTATCCGTACTCCTGATGATGCATGGTTACTCACCACTGCGATC<br>CCCGGGAAAACAGCATTCCAGGTATTAGAAGAATATCCTGATTCAGGTGAAAATATTGTTGATGCGCT<br>GGCAGTGTTCCTGCGCCGGTTGCATTCGATTCCTGTTTGTAATTGTCCTTTTAACAGCGATCGCGTATT<br>TCGTCTCGCTCAGGCGCAATCACGAATGAATAACGGTTTGGTTGATGCGAGTGATTTTGATGACGAGC<br>GTAATGGCTGGCCTGTTGAACAAGTCTGGAAAGAAATGCATAAACTTTTGCCATTCTCACCGGATTCA<br>GTCGTCACTCATGGTGATTTCTCACTTGATAACCTTATTTTTGACGAGGGGAAATTAATAGGTTGTATT<br>GATGTTGGACGAGTCGGAATCGCAGACCGATACCAGGATCTTGCCATCCTATGGAACTGCCTCGGTG<br>AGTTTTCTCCTTCATTACAGAAACGGCTTTTTCAAAAATATGGTATTGATAATCCTGATATGAATAAATT<br>GCAGTTTCATTTGATGCTCGATGAGTTTTTCTAAGCTGTCAGACCAAGTTTACTCATATATACTTTAGAT<br>TGATTTAAAACTTCATTTTTAATTTAAAAGGATCTAGGTGAAGATCCTTTTTGATAATCTCATGACCAAA<br>ATCCCTTAACGTGAGTTTTCGTTCCACTGAGCGTCAGACCCCGTAGAAAAGATCAAAGGATCTTCTTGA<br>GATCCTTTTTTTCTGCGCGTAATCTGCTGCTTGCAAACAAAAAAACCACCGCTACCAGCGGTGGTTTGT<br>TTGCCGGATCAAGAGCTACCAACTCTTTTTCCGAAGGTAACTGGCTTCAGCAGAGCGCAGATACCAAA<br>TACTGTCCTTCTAGTGTAGCCGTAGTTAGGCCACCACTTCAAGAACTCTGTAGCACCGCCTACATACCT<br>CGCTCTGCTAATCCTGTTACCAGTGGCTGCTGCCAGTGGCGATAAGTCGTGTCTTACCGGGTTGGACT<br>CAAGACGATAGTTACCGGATAAGGCGCAGCGGTCGGGCTGAACGGGGGGTTCGTGCACACAGCCCA<br>GCTTGGAGCGAACGACCTACACCGAACTGAGATACCTACAGCGTGAGCTATGAGAAAGCGCCACGCT<br>TCCCGAAGGGAGAAAGGCGGACAGGTATCCGGTAAGCGGCAGGGTCGGAACAGGAGAGCGCACGA<br>GGGAGCTTCCAGGGGGAAACGCCTGGTATCTTTATAGTCCTGTCGGGTTTCGCCACCTCTGACTTGAG<br>CGTCGATTTTTGTGATGCTCGTCAGGGGGGCGGAGCCTATGGAAAAACGCCAGCAACGCGGCCTTTT<br>TACGGTTCCTGGCCTTTTGCTGGCCTTTTGCTCACATGTTCTTTCCTGCGTTATCCCCTGATTCTGTGGA<br>TAACCGTATTACCGCCTTTGAGTGAGCTGATACCGCTCGCCGCAGCCGAACGACCGAGCGCAGCGAG

	TCAGTGAGCGAGGAAGCGGAAGAGCGCCTGATGCGGTATTTTCTCCTTACGCATCTGTGCGGTATTTC<br>ACACCGCATATGGTGCACTCTCAGTACAATCTGCTCTGATGCCGCATAGTTAAGCCAGTATACACTCCG<br>CTATCGCTACGTGACTGGGTCATGGCTGCGCCCCGACACCCGCCAACACCCGCTGACGCGCCCTGACG<br>GGCTTGTCTGCTCCCGGCATCCGCTTACAGACAAGCTGTGACCGTCTCCGGGAGCTGCATGTGTCAGA<br>GGTTTTCACCGTCATCACCGAAACGCGCGAGGCAGCAGATCAATTCGCGCGCGAAGGCGAAGCGGCA<br>TGCATAATGTGCCTGTCAAATGGACGAAGCAGGGATTCTGCAAACCCTATGCTACTCCGTCAAGCCGT<br>CAATTGTCTGATTCGTTACCAATTATGACAACTTGACGGCTACATCATTCACTTTTTCTTCACAACCGGC<br>ACGGAACTCGCTCGGGCTGGCCCCGGTGCATTTTTTAAATACCCGCGAGAAATAGAGTTGATCGTCAA<br>AACCAACATTGCGACCGACGGTGGCGATAGGCATCCGGGTGGTGCTCAAAAGCAGCTTCGCCTGGCT<br>GATACGTTGGTCCTCGCGCCAGCTTAAGACGCTAATCCCTAACTGCTGGCGGAAAAGATGTGACAGA<br>CGCGACGGCGACAAGCAAACATGCTGTGCGACGCTGGCGATATCAAAATTGCTGTCTGCCAGGTGAT<br>CGCTGATGTACTGACAAGCCTCGCGTACCCGATTATCCATCGGTGGATGGAGCGACTCGTTAATCGCT<br>TCCATGCGCCGCAGTAACAATTGCTCAAGCAGATTTATCGCCAGCAGCTCCGAATAGCGCCCTTCCCCT<br>TGCCCGGCGTTAATGATTTGCCCAAACAGGTCGCTGAAATGCGGCTGGTGCGCTTCATCCGGGCGAA<br>AGAACCCCGTATTGGCAAATATTGACGGCCAGTTAAGCCATTCATGCCAGTAGGCGCGCGGACGAAA<br>GTAAACCCACTGGTGATACCATTCGCGAGCCTCCGGATGACGACCGTAGTGATGAATCTCTCCTGGCG<br>GGAACAGCAAAATATCACCCGGTCGGCAAACAAATTCTCGTCCCTGATTTTTCACCACCCCCTGACCGC<br>GAATGGTGAGATTGAGAATATAACCTTTCATTCCCAGCGGTCGGTCGATAAAAAAATCGAGATAACC<br>GTTGGCCTCAATCGGCGTTAAACCCGCCACCAGATGGGCATTAAACGAGTATCCCGGCAGCAGGGGA<br>TCATTTTGCGCTTCAGCCATACTTTTCATACTCCCGCCATTCAGAG
pBAD:pre^{MalE}-MalE-TCD-mCherry	AAGAAACCAATTGTCCATATTGCATCAGACATTGCCGTCACTGCGTCTTTTACTGGCTCTTCTCGCTAAC<br>CAAACCGGTAACCCCGCTTATTAAAAGCATTCTGTAACAAAGCGGGACCAAAGCCATGACAAAAACG<br>CGTAACAAAAGTGTCTATAATCACGGCAGAAAAGTCCACATTGATTATTTGCACGGCGTCACACTTTG<br>CTATGCCATAGCATTTTTATCCATAAGATTAGCGGATCCTACCTGACGCTTTTTATCGCAACTCTCTACT<br>GTTTCTCCATACCCGTTTTTTTGGGCTAGAAATAATTTTGTTTAACTTTAAGAAGGAGATATACATATGA<br>AAATAAAAACAGGTGCACGCATCCTCGCATTATCCGCATTAACGACGATGATGTTTTCCGCCTCGGCTC<br>TCGCCAAAATCGAAGAAGGTAAACTGGTAATCTGGATTAACGGCGATAAAGGCTATAACGGTCTCGC<br>TGAAGTCGGTAAGAAATTCGAGAAAGATACCGGAATTAAAGTCACCGTTGAGCATCCGGATAAACTG<br>GAAGAGAAATTCCCACAGGTTGCGGCAACTGGCGATGGCCCTGACATTATCTTCTGGGCACACGACC<br>GCTTTGGTGGCTACGCTCAATCTGGCCTGTTGGCTGAAATCACCCCGGACAAAGCGTTCCAGGACAAG<br>CTGTATCCGTTTACCTGGGATGCCGTACGTTACAACGGCAAGCTGATTGCTTACCCGATCGCTGTTGAA<br>GCGTTATCGCTGATTTATAACAAAGATCTGCTGCCGAACCCGCCAAAAACCTGGGAAGAGATCCCGGC<br>GCTGGATAAAGAACTGAAAGCGAAAGGTAAGAGCGCGCTGATGTTCAACCTGCAAGAACCGTACTTC<br>ACCTGGCCGCTGATTGCTGCTGACGGGGGTTATGCGTTCAAGTATGAAAACGGCAAGTACGACATTA<br>AAGACGTGGGCGTGGATAACGCTGGCGCGAAAGCGGGTCTGACCTTCCTGGTTGACCTGATTAAAAA<br>CAAACACATGAATGCAGACACCGATTACTCCATCGCAGAAGCTGCCTTTAATAAAGGCGAAACAGCG<br>ATGACCATCAACGGCCCGTGGGCATGGTCCAACATCGACACCAGCAAAGTGAATTATGGTGTAACGG<br>TACTGCCGACCTTCAAGGGTCAACCATCCAAACCGTTCGTTGGCGTGCTGAGCGCAGGTATTAACGCC<br>GCCAGTCCGAACAAAGAGCTGGCGAAAGAGTTCCTCGAAAACTATCTGCTGACTGATGAAGGTCTGG<br>AAGCGGTTAATAAAGACAAACCGCTGGGTGCCGTAGCGCTGAAGTCTTACGAGGAAGAGTTGGCGA<br>AAGATCCACGTATTGCCGCCACCATGGAAAACGCCCAGAAAGGTGAAATCATGCCGAACATCCCGCA<br>GATGTCCGCTTTCTGGTATGCCGTGCGTACTGCGGTGATCAACGCCGCCAGCGGTCGTCAGACTGTCG<br>ATGAAGCCCTGAAAGACGCGCAGACTACTAGTCATCATCACCACCATCATTAGGATGGTGGTGATGAT<br>AATGATGAGCAAGGGCGAGGAGGATAACATGGCCATCATCAAGGAGTTCATGCGCTTCAAGGTGCA<br>CATGGAGGGCTCCGTGAACGGCCACGAGTTCGAGATCGAGGGCGAGGGCGAGGGCCGCCCCTACGA<br>GGGCACCCAGACCGCCAAGCTGAAGGTGACCAAGGGTGGCCCCCTGCCCTTCGCCTGGGACATCCTG<br>TCCCCTCAGTTCATGTACGGCTCCAAGGCCTACGTGAAGCACCCCGCCGACATCCCCGACTACTTGAA<br>GCTGTCCTTCCCCGAGGGCTTCAAGTGGGAGCGCGTGATGAACTTCGAGGACGGCGGCGTGGTGACC<br>GTGACCCAGGACTCCTCCCTGCAGGACGGCGAGTTCATCTACAAGGTGAAGCTGCGCGGCACCAACT<br>TCCCCTCCGACGGCCCCGTAATGCAGAAGAAGACCATGGGCTGGGAGGCCTCCTCCGAGCGGATGTA<br>CCCCGAGGACGGCGCCCTGAAGGGCGAGATCAAGCAGAGGCTGAAGCTGAAGGACGGCGGCCACT<br>ACGACGCTGAGGTCAAGACCACCTACAAGGCCAAGAAGCCCGTGCAGCTGCCCGGCGCCTACAACGT<br>CAACATCAAGTTGGACATCACCTCCCACAACGAGGACTACACCATCGTGGAACAGTACGAACGCGCC<br>GAGGGCCGCCACTCCACCGGCGGCATGGACGAGCTGTACAAGTGAAAGCTTACGGTCTCCAGCTTGG<br>CTGTTTTGGCGGATGAGAGAAGATTTTCAGCCTGATACAGATTAAATCAGAACGCAGAAGCGGTCTG<br>ATAAAACAGAATTTGCCTGGCGGCAGTAGCGCGGTGGTCCCACCTGACCCCATGCCGAACTCAGAAG<br>TGAAACGCCGTAGCGCCGATGGTAGTGTGGGGTCTCCCCATGCGAGAGTAGGGAACTGCCAGGCATC<br>AAATAAAACGAAAGGCTCAGTCGAAAGACTGGGCCTTTCGTTTTATCTGTTGTTTGTCGGTGAACGCT<br>CTCCTGAGTAGGACAAATCCGCCGGGAGCGGATTTGAACGTTGCGAAGCAACGGCCCGGAGGGTGG<br>CGGGCAGGACGCCCGCCATAAACTGCCAGGCATCAAATTAAGCAGAAGGCCATCCTGACGGATGGCC<br>TTTTTGCGTTTCTACAAACTCTCATGAACAATAAAACTGTCTGCTTACATAAACAGTAATACAAGGGGT<br>GTTATGAGCCATATTCAACGGGAAACGTCTTGCTCTAGGCCGCGATTAAATTCCAACATGGATGCTGA

	TTTATATGGGTATAAATGGGCTCGCGATAATGTCGGGCAATCAGGTGCGACAATCTATCGATTGTATG<br>GGAAGCCCGATGCGCCAGAGTTGTTTCTGAAACATGGCAAAGGTAGCGTTGCCAATGATGTTACAGA<br>TGAGATGGTCAGACTAAACTGGCTGACGGAATTTATGCCTCTTCCGACCATCAAGCATTTTATCCGTAC<br>TCCTGATGATGCATGGTTACTCACCACTGCGATCCCCGGGAAAACAGCATTCCAGGTATTAGAAGAAT<br>ATCCTGATTCAGGTGAAAATATTGTTGATGCGCTGGCAGTGTTCCTGCGCCGGTTGCATTCGATTCCTG<br>TTTGTAATTGTCCTTTTAACAGCGATCGCGTATTTCGTCTCGCTCAGGCGCAATCACGAATGAATAACG<br>GTTTGGTTGATGCGAGTGATTTTGATGACGAGCGTAATGGCTGGCCTGTTGAACAAGTCTGGAAAGA<br>AATGCATAAACTTTTGCCATTCTCACCGGATTCAGTCGTCACTCATGGTGATTTCTCACTTGATAACCTT<br>ATTTTTGACGAGGGGAAATTAATAGGTTGTATTGATGTTGGACGAGTCGGAATCGCAGACCGATACC<br>AGGATCTTGCCATCCTATGGAACTGCCTCGGTGAGTTTTCTCCTTCATTACAGAAACGGCTTTTTCAAA<br>AATATGGTATTGATAATCCTGATATGAATAAATTGCAGTTTCATTTGATGCTCGATGAGTTTTTCTAAG<br>CTGTCAGACCAAGTTTACTCATATATACTTTAGATTGATTTAAAACTTCATTTTTAATTTAAAAGGATCT<br>AGGTGAAGATCCTTTTTGATAATCTCATGACCAAAATCCCTTAACGTGAGTTTTCGTTCCACTGAGCGT<br>CAGACCCCGTAGAAAAGATCAAAGGATCTTCTTGAGATCCTTTTTTTCTGCGCGTAATCTGCTGCTTGC<br>AAACAAAAAAACCACCGCTACCAGCGGTGGTTTGTTTGCCGGATCAAGAGCTACCAACTCTTTTTCCG<br>AAGGTAACTGGCTTCAGCAGAGCGCAGATACCAAATACTGTCCTTCTAGTGTAGCCGTAGTTAGGCCA<br>CCACTTCAAGAACTCTGTAGCACCGCCTACATACCTCGCTCTGCTAATCCTGTTACCAGTGGCTGCTGC<br>CAGTGGCGATAAGTCGTGTCTTACCGGGTTGGACTCAAGACGATAGTTACCGGATAAGGCGCAGCGG<br>TCGGGCTGAACGGGGGGTTCGTGCACACAGCCCAGCTTGGAGCGAACGACCTACACCGAACTGAGAT<br>ACCTACAGCGTGAGCTATGAGAAAGCGCCACGCTTCCCGAAGGGAGAAAGGCGGACAGGTATCCGG<br>TAAGCGGCAGGGTCGGAACAGGAGAGCGCACGAGGGAGCTTCCAGGGGGAAACGCCTGGTATCTTT<br>ATAGTCCTGTCGGGTTTCGCCACCTCTGACTTGAGCGTCGATTTTTGTGATGCTCGTCAGGGGGGCGG<br>AGCCTATGGAAAAACGCCAGCAACGCGGCCTTTTTACGGTTCCTGGCCTTTTGCTGGCCTTTTGCTCAC<br>ATGTTCTTTCCTGCGTTATCCCCTGATTCTGTGGATAACCGTATTACCGCCTTTGAGTGAGCTGATACC<br>GCTCGCCGCAGCCGAACGACCGAGCGCAGCGAGTCAGTGAGCGAGGAAGCGGAAGAGCGCCTGAT<br>GCGGTATTTTCTCCTTACGCATCTGTGCGGTATTTCACACCGCATATGGTGCACTCTCAGTACAATCTG<br>CTCTGATGCCGCATAGTTAAGCCAGTATACACTCCGCTATCGCTACGTGACTGGGTCATGGCTGCGCC<br>CCGACACCCGCCAACACCCGCTGACGCGCCCTGACGGGCTTGTCTGCTCCCGGCATCCGCTTACAGAC<br>AAGCTGTGACCGTCTCCGGGAGCTGCATGTGTCAGAGGTTTTCACCGTCATCACCGAAACGCGCGAG<br>GCAGCAGATCAATTCGCGCGCGAAGGCGAAGCGGCATGCATAATGTGCCTGTCAAATGGACGAAGC<br>AGGGATTCTGCAAACCCTATGCTACTCCGTCAAGCCGTCAATTGTCTGATTCGTTACCAATTATGACAA<br>CTTGACGGCTACATCATTCACTTTTTCTTCACAACCGGCACGGAACTCGCTCGGGCTGGCCCCGGTGCA<br>TTTTTTAAATACCCGCGAGAAATAGAGTTGATCGTCAAAACCAACATTGCGACCGACGGTGGCGATAG<br>GCATCCGGGTGGTGCTCAAAAGCAGCTTCGCCTGGCTGATACGTTGGTCCTCGCGCCAGCTTAAGACG<br>CTAATCCCTAACTGCTGGCGGAAAAGATGTGACAGACGCGACGGCGACAAGCAAACATGCTGTGCGA<br>CGCTGGCGATATCAAAATTGCTGTCTGCCAGGTGATCGCTGATGTACTGACAAGCCTCGCGTACCCGA<br>TTATCCATCGGTGGATGGAGCGACTCGTTAATCGCTTCCATGCGCCGCAGTAACAATTGCTCAAGCAG<br>ATTTATCGCCAGCAGCTCCGAATAGCGCCCTTCCCCTTGCCCGGCGTTAATGATTTGCCCAAACAGGTC<br>GCTGAAATGCGGCTGGTGCGCTTCATCCGGGCGAAAGAACCCCGTATTGGCAAATATTGACGGCCAG<br>TTAAGCCATTCATGCCAGTAGGCGCGCGGACGAAAGTAAACCCACTGGTGATACCATTCGCGAGCCTC<br>CGGATGACGACCGTAGTGATGAATCTCTCCTGGCGGGAACAGCAAAATATCACCCGGTCGGCAAACA<br>AATTCTCGTCCCTGATTTTTCACCACCCCCTGACCGCGAATGGTGAGATTGAGAATATAACCTTTCATTC<br>CCAGCGGTCGGTCGATAAAAAAATCGAGATAACCGTTGGCCTCAATCGGCGTTAAACCCGCCACCAG<br>ATGGGCATTAAACGAGTATCCCGGCAGCAGGGGATCATTTTGCGCTTCAGCCATACTTTTCATACTCCC<br>GCCATTCAGAG
pBAD:pre^{DsbA}-MalE-TCD-mCherry	AAGAAACCAATTGTCCATATTGCATCAGACATTGCCGTCACTGCGTCTTTTACTGGCTCTTCTCGCTAAC<br>CAAACCGGTAACCCCGCTTATTAAAAGCATTCTGTAACAAAGCGGGACCAAAGCCATGACAAAAACG<br>CGTAACAAAAGTGTCTATAATCACGGCAGAAAAGTCCACATTGATTATTTGCACGGCGTCACACTTTG<br>CTATGCCATAGCATTTTTATCCATAAGATTAGCGGATCCTACCTGACGCTTTTTATCGCAACTCTCTACT<br>GTTTCTCCATACCCGTTTTTTTGGGCTAGAAATAATTTTGTTTAACTTTAAGAAGGAGATATACATATGA<br>AAAAGATTTGGCTGGCGCTGGCTGGTTTAGTTTTAGCGTTTAGCGCATCGGCGAAAATCGAAGAAGG<br>TAAACTGGTAATCTGGATTAACGGCGATAAAGGCTATAACGGTCTCGCTGAAGTCGGTAAGAAATTC<br>GAGAAAGATACCGGAATTAAAGTCACCGTTGAGCATCCGGATAAACTGGAAGAGAAATTCCCACAGG<br>TTGCGGCAACTGGCGATGGCCCTGACATTATCTTCTGGGCACACGACCGCTTTGGTGGCTACGCTCAA<br>TCTGGCCTGTTGGCTGAAATCACCCCGGACAAAGCGTTCCAGGACAAGCTGTATCCGTTTACCTGGGA<br>TGCCGTACGTTACAACGGCAAGCTGATTGCTTACCCGATCGCTGTTGAAGCGTTATCGCTGATTTATAA<br>CAAAGATCTGCTGCCGAACCCGCCAAAAACCTGGGAAGAGATCCCGGCGCTGGATAAAGAACTGAAA<br>GCGAAAGGTAAGAGCGCGCTGATGTTCAACCTGCAAGAACCGTACTTCACCTGGCCGCTGATTGCTG<br>CTGACGGGGGTTATGCGTTCAAGTATGAAAACGGCAAGTACGACATTAAAGACGTGGGCGTGGATA<br>ACGCTGGCGCGAAAGCGGGTCTGACCTTCCTGGTTGACCTGATTAAAAACAAACACATGAATGCAGA<br>CACCGATTACTCCATCGCAGAAGCTGCCTTTAATAAAGGCGAAACAGCGATGACCATCAACGGCCCGT<br>GGGCATGGTCCAACATCGACACCAGCAAAGTGAATTATGGTGTAACGGTACTGCCGACCTTCAAGGG

	TCAACCATCCAAACCGTTCGTTGGCGTGCTGAGCGCAGGTATTAACGCCGCCAGTCCGAACAAAGAG<br>CTGGCGAAAGAGTTCCTCGAAAACTATCTGCTGACTGATGAAGGTCTGGAAGCGGTTAATAAAGACA<br>AACCGCTGGGTGCCGTAGCGCTGAAGTCTTACGAGGAAGAGTTGGCGAAAGATCCACGTATTGCCGC<br>CACCATGGAAAACGCCCAGAAAGGTGAAATCATGCCGAACATCCCGCAGATGTCCGCTTTCTGGTATG<br>CCGTGCGTACTGCGGTGATCAACGCCGCCAGCGGTCGTCAGACTGTCGATGAAGCCCTGAAAGACGC<br>GCAGACTACTAGTCATCATCACCACCATCATTAGGATGGTGGTGATGATAATGATGAGCAAGGGCGA<br>GGAGGATAACATGGCCATCATCAAGGAGTTCATGCGCTTCAAGGTGCACATGGAGGGCTCCGTGAAC<br>GGCCACGAGTTCGAGATCGAGGGCGAGGGCGAGGGCCGCCCCTACGAGGGCACCCAGACCGCCAA<br>GCTGAAGGTGACCAAGGGTGGCCCCCTGCCCTTCGCCTGGGACATCCTGTCCCCTCAGTTCATGTACG<br>GCTCCAAGGCCTACGTGAAGCACCCCGCCGACATCCCCGACTACTTGAAGCTGTCCTTCCCCGAGGGC<br>TTCAAGTGGGAGCGCGTGATGAACTTCGAGGACGGCGGCGTGGTGACCGTGACCCAGGACTCCTCCC<br>TGCAGGACGGCGAGTTCATCTACAAGGTGAAGCTGCGCGGCACCAACTTCCCCTCCGACGGCCCCGT<br>AATGCAGAAGAAGACCATGGGCTGGGAGGCCTCCTCCGAGCGGATGTACCCCGAGGACGGCGCCCT<br>GAAGGGCGAGATCAAGCAGAGGCTGAAGCTGAAGGACGGCGGCCACTACGACGCTGAGGTCAAGA<br>CCACCTACAAGGCCAAGAAGCCCGTGCAGCTGCCCGGCGCCTACAACGTCAACATCAAGTTGGACAT<br>CACCTCCCACAACGAGGACTACACCATCGTGGAACAGTACGAACGCGCCGAGGGCCGCCACTCCACC<br>GGCGGCATGGACGAGCTGTACAAGTGAAAGCTTACGGTCTCCAGCTTGGCTGTTTTGGCGGATGAGA<br>GAAGATTTTCAGCCTGATACAGATTAAATCAGAACGCAGAAGCGGTCTGATAAAACAGAATTTGCCTG<br>GCGGCAGTAGCGCGGTGGTCCCACCTGACCCCATGCCGAACTCAGAAGTGAAACGCCGTAGCGCCGA<br>TGGTAGTGTGGGGTCTCCCCATGCGAGAGTAGGGAACTGCCAGGCATCAAATAAAACGAAAGGCTCA<br>GTCGAAAGACTGGGCCTTTCGTTTTATCTGTTGTTTGTCGGTGAACGCTCTCCTGAGTAGGACAAATCC<br>GCCGGGAGCGGATTTGAACGTTGCGAAGCAACGGCCCGGAGGGTGGCGGGCAGGACGCCCGCCAT<br>AAACTGCCAGGCATCAAATTAAGCAGAAGGCCATCCTGACGGATGGCCTTTTTGCGTTTCTACAAACT<br>CTCATGAACAATAAAACTGTCTGCTTACATAAACAGTAATACAAGGGGTGTTATGAGCCATATTCAAC<br>GGGAAACGTCTTGCTCTAGGCCGCGATTAAATTCCAACATGGATGCTGATTTATATGGGTATAAATGG<br>GCTCGCGATAATGTCGGGCAATCAGGTGCGACAATCTATCGATTGTATGGGAAGCCCGATGCGCCAG<br>AGTTGTTTCTGAAACATGGCAAAGGTAGCGTTGCCAATGATGTTACAGATGAGATGGTCAGACTAAA<br>CTGGCTGACGGAATTTATGCCTCTTCCGACCATCAAGCATTTTATCCGTACTCCTGATGATGCATGGTT<br>ACTCACCACTGCGATCCCCGGGAAAACAGCATTCCAGGTATTAGAAGAATATCCTGATTCAGGTGAAA<br>ATATTGTTGATGCGCTGGCAGTGTTCCTGCGCCGGTTGCATTCGATTCCTGTTTGTAATTGTCCTTTTAA<br>CAGCGATCGCGTATTTCGTCTCGCTCAGGCGCAATCACGAATGAATAACGGTTTGGTTGATGCGAGTG<br>ATTTTGATGACGAGCGTAATGGCTGGCCTGTTGAACAAGTCTGGAAAGAAATGCATAAACTTTTGCCA<br>TTCTCACCGGATTCAGTCGTCACTCATGGTGATTTCTCACTTGATAACCTTATTTTTGACGAGGGGAAA<br>TTAATAGGTTGTATTGATGTTGGACGAGTCGGAATCGCAGACCGATACCAGGATCTTGCCATCCTATG<br>GAACTGCCTCGGTGAGTTTTCTCCTTCATTACAGAAACGGCTTTTTCAAAAATATGGTATTGATAATCC<br>TGATATGAATAAATTGCAGTTTCATTTGATGCTCGATGAGTTTTTCTAAGCTGTCAGACCAAGTTTACT<br>CATATATACTTTAGATTGATTTAAAACTTCATTTTTAATTTAAAAGGATCTAGGTGAAGATCCTTTTTGA<br>TAATCTCATGACCAAAATCCCTTAACGTGAGTTTTCGTTCCACTGAGCGTCAGACCCCGTAGAAAAGAT<br>CAAAGGATCTTCTTGAGATCCTTTTTTTCTGCGCGTAATCTGCTGCTTGCAAACAAAAAAACCACCGCT<br>ACCAGCGGTGGTTTGTTTGCCGGATCAAGAGCTACCAACTCTTTTTCCGAAGGTAACTGGCTTCAGCA<br>GAGCGCAGATACCAAATACTGTCCTTCTAGTGTAGCCGTAGTTAGGCCACCACTTCAAGAACTCTGTA<br>GCACCGCCTACATACCTCGCTCTGCTAATCCTGTTACCAGTGGCTGCTGCCAGTGGCGATAAGTCGTGT<br>CTTACCGGGTTGGACTCAAGACGATAGTTACCGGATAAGGCGCAGCGGTCGGGCTGAACGGGGGGT<br>TCGTGCACACAGCCCAGCTTGGAGCGAACGACCTACACCGAACTGAGATACCTACAGCGTGAGCTAT<br>GAGAAAGCGCCACGCTTCCCGAAGGGAGAAAGGCGGACAGGTATCCGGTAAGCGGCAGGGTCGGA<br>ACAGGAGAGCGCACGAGGGAGCTTCCAGGGGGAAACGCCTGGTATCTTTATAGTCCTGTCGGGTTTC<br>GCCACCTCTGACTTGAGCGTCGATTTTTGTGATGCTCGTCAGGGGGGCGGAGCCTATGGAAAAACGC<br>CAGCAACGCGGCCTTTTTACGGTTCCTGGCCTTTTGCTGGCCTTTTGCTCACATGTTCTTTCCTGCGTTA<br>TCCCCTGATTCTGTGGATAACCGTATTACCGCCTTTGAGTGAGCTGATACCGCTCGCCGCAGCCGAAC<br>GACCGAGCGCAGCGAGTCAGTGAGCGAGGAAGCGGAAGAGCGCCTGATGCGGTATTTTCTCCTTAC<br>GCATCTGTGCGGTATTTCACACCGCATATGGTGCACTCTCAGTACAATCTGCTCTGATGCCGCATAGTT<br>AAGCCAGTATACACTCCGCTATCGCTACGTGACTGGGTCATGGCTGCGCCCCGACACCCGCCAACACC<br>CGCTGACGCGCCCTGACGGGCTTGTCTGCTCCCGGCATCCGCTTACAGACAAGCTGTGACCGTCTCCG<br>GGAGCTGCATGTGTCAGAGGTTTTCACCGTCATCACCGAAACGCGCGAGGCAGCAGATCAATTCGCG<br>CGCGAAGGCGAAGCGGCATGCATAATGTGCCTGTCAAATGGACGAAGCAGGGATTCTGCAAACCCTA<br>TGCTACTCCGTCAAGCCGTCAATTGTCTGATTCGTTACCAATTATGACAACTTGACGGCTACATCATTC<br>ACTTTTTCTTCACAACCGGCACGGAACTCGCTCGGGCTGGCCCCGGTGCATTTTTTAAATACCCGCGAG<br>AAATAGAGTTGATCGTCAAAACCAACATTGCGACCGACGGTGGCGATAGGCATCCGGGTGGTGCTCA<br>AAAGCAGCTTCGCCTGGCTGATACGTTGGTCCTCGCGCCAGCTTAAGACGCTAATCCCTAACTGCTGG<br>CGGAAAAGATGTGACAGACGCGACGGCGACAAGCAAACATGCTGTGCGACGCTGGCGATATCAAAA<br>TTGCTGTCTGCCAGGTGATCGCTGATGTACTGACAAGCCTCGCGTACCCGATTATCCATCGGTGGATG<br>GAGCGACTCGTTAATCGCTTCCATGCGCCGCAGTAACAATTGCTCAAGCAGATTTATCGCCAGCAGCT<br>CCGAATAGCGCCCTTCCCCTTGCCCGGCGTTAATGATTTGCCCAAACAGGTCGCTGAAATGCGGCTGG

	TGCGCTTCATCCGGGCGAAAGAACCCCGTATTGGCAAATATTGACGGCCAGTTAAGCCATTCATGCCA GTAGGCGCGCGGACGAAAGTAAACCCACTGGTGATACCATTCGCGAGCCTCCGGATGACGACCGTAG TGATGAATCTCTCCTGGCGGGAACAGCAAAATATCACCCGGTCGGCAAACAAATTCTCGTCCCTGATT TTTCACCACCCCCTGACCGCGAATGGTGAGATTGAGAATATAACCTTTCATTCCCAGCGGTCGGTCGAT AAAAAAATCGAGATAACCGTTGGCCTCAATCGGCGTTAAACCCGCCACCAGATGGGCATTAAACGAG TATCCCGGCAGCAGGGGATCATTTTGCGCTTCAGCCATACTTTTCATACTCCCGCCATTCAGAG
pBAD:Δss-MalE-TCD-mCherry	AAGAAACCAATTGTCCATATTGCATCAGACATTGCCGTCACTGCGTCTTTTACTGGCTCTTCTCGCTAAC CAAACCGGTAACCCCGCTTATTAAAAGCATTCTGTAACAAAGCGGGACCAAAGCCATGACAAAAACG CGTAACAAAAGTGTCTATAATCACGGCAGAAAAGTCCACATTGATTATTTGCACGGCGTCACACTTTG CTATGCCATAGCATTTTTATCCATAAGATTAGCGGATCCTACCTGACGCTTTTTATCGCAACTCTCTACT GTTTCTCCATACCCGTTTTTTTGGGCTAGAAATAATTTTGTTTAACTTTAAGAAGGAGATATACATATGA AAATCGAAGAAGGTAAACTGGTAATCTGGATTAACGGCGATAAAGGCTATAACGGTCTCGCTGAAGT CGGTAAGAAATTCGAGAAAGATACCGGAATTAAAGTCACCGTTGAGCATCCGGATAAACTGGAAGAG AAATTCCCACAGGTTGCGGCAACTGGCGATGGCCCTGACATTATCTTCTGGGCACACGACCGCTTTGG TGGCTACGCTCAATCTGGCCTGTTGGCTGAAATCACCCCGGACAAAGCGTTCCAGGACAAGCTGTATC CGTTTACCTGGGATGCCGTACGTTACAACGGCAAGCTGATTGCTTACCCGATCGCTGTTGAAGCGTTA TCGCTGATTTATAACAAAGATCTGCTGCCGAACCCGCCAAAAACCTGGGAAGAGATCCCGGCGCTGG ATAAAGAACTGAAAGCGAAAGGTAAGAGCGCGCTGATGTTCAACCTGCAAGAACCGTACTTCACCTG GCCGCTGATTGCTGCTGACGGGGGTTATGCGTTCAAGTATGAAAACGGCAAGTACGACATTAAAGAC GTGGGCGTGGATAACGCTGGCGCGAAAGCGGGTCTGACCTTCCTGGTTGACCTGATTAAAAACAAAC ACATGAATGCAGACACCGATTACTCCATCGCAGAAGCTGCCTTTAATAAAGGCGAAACAGCGATGACC ATCAACGGCCCGTGGGCATGGTCCAACATCGACACCAGCAAAGTGAATTATGGTGTAACGGTACTGC CGACCTTCAAGGGTCAACCATCCAAACCGTTCGTTGGCGTGCTGAGCGCAGGTATTAACGCCGCCAGT CCGAACAAAGAGCTGGCGAAAGAGTTCCTCGAAAACTATCTGCTGACTGATGAAGGTCTGGAAGCGG TTAATAAAGACAAACCGCTGGGTGCCGTAGCGCTGAAGTCTTACGAGGAAGAGTTGGCGAAAGATCC ACGTATTGCCGCCACCATGGAAAACGCCCAGAAAGGTGAAATCATGCCGAACATCCCGCAGATGTCC GCTTTCTGGTATGCCGTGCGTACTGCGGTGATCAACGCCGCCAGCGGTCGTCAGACTGTCGATGAAGC CCTGAAAGACGCGCAGACTACTAGTCATCATCACCACCATCATTAGGATGGTGGTGATGATAATGATG AGCAAGGGCGAGGAGGATAACATGGCCATCATCAAGGAGTTCATGCGCTTCAAGGTGCACATGGAG GGCTCCGTGAACGGCCACGAGTTCGAGATCGAGGGCGAGGGCGAGGGCCGCCCCTACGAGGGCACC CAGACCGCCAAGCTGAAGGTGACCAAGGGTGGCCCCCTGCCCTTCGCCTGGGACATCCTGTCCCCTCA GTTCATGTACGGCTCCAAGGCCTACGTGAAGCACCCCGCCGACATCCCCGACTACTTGAAGCTGTCCT TCCCCGAGGGCTTCAAGTGGGAGCGCGTGATGAACTTCGAGGACGGCGGCGTGGTGACCGTGACCC AGGACTCCTCCCTGCAGGACGGCGAGTTCATCTACAAGGTGAAGCTGCGCGGCACCAACTTCCCCTCC GACGGCCCCGTAATGCAGAAGAAGACCATGGGCTGGGAGGCCTCCTCCGAGCGGATGTACCCCGAG GACGGCGCCCTGAAGGGCGAGATCAAGCAGAGGCTGAAGCTGAAGGACGGCGGCCACTACGACGCT GAGGTCAAGACCACCTACAAGGCCAAGAAGCCCGTGCAGCTGCCCGGCGCCTACAACGTCAACATCA AGTTGGACATCACCTCCCACAACGAGGACTACACCATCGTGGAACAGTACGAACGCGCCGAGGGCCG CCACTCCACCGGCGGCATGGACGAGCTGTACAAGTGAAAGCTTACGGTCTCCAGCTTGGCTGTTTTGG CGGATGAGAGAAGATTTTCAGCCTGATACAGATTAAATCAGAACGCAGAAGCGGTCTGATAAAACAG AATTTGCCTGGCGGCAGTAGCGCGGTGGTCCCACCTGACCCCATGCCGAACTCAGAAGTGAAACGCC GTAGCGCCGATGGTAGTGTGGGGTCTCCCCATGCGAGAGTAGGGAACTGCCAGGCATCAAATAAAAC GAAAGGCTCAGTCGAAAGACTGGGCCTTTCGTTTTATCTGTTGTTTGTCGGTGAACGCTCTCCTGAGT AGGACAAATCCGCCGGGAGCGGATTTGAACGTTGCGAAGCAACGGCCCGGAGGGTGGCGGGCAGG ACGCCCGCCATAAACTGCCAGGCATCAAATTAAGCAGAAGGCCATCCTGACGGATGGCCTTTTTGCGT TTCTACAAACTCTCATGAACAATAAAACTGTCTGCTTACATAAACAGTAATACAAGGGGTGTTATGAG CCATATTCAACGGGAAACGTCTTGCTCTAGGCCGCGATTAAATTCCAACATGGATGCTGATTTATATG GGTATAAATGGGCTCGCGATAATGTCGGGCAATCAGGTGCGACAATCTATCGATTGTATGGGAAGCC CGATGCGCCAGAGTTGTTTCTGAAACATGGCAAAGGTAGCGTTGCCAATGATGTTACAGATGAGATG GTCAGACTAAACTGGCTGACGGAATTTATGCCTCTTCCGACCATCAAGCATTTTATCCGTACTCCTGAT GATGCATGGTTACTCACCACTGCGATCCCCGGGAAAACAGCATTCCAGGTATTAGAAGAATATCCTGA TTCAGGTGAAAATATTGTTGATGCGCTGGCAGTGTTCCTGCGCCGGTTGCATTCGATTCCTGTTTGTAA TTGTCCTTTTAACAGCGATCGCGTATTTCGTCTCGCTCAGGCGCAATCACGAATGAATAACGGTTTGGT TGATGCGAGTGATTTTGATGACGAGCGTAATGGCTGGCCTGTTGAACAAGTCTGGAAAGAAATGCAT AAACTTTTGCCATTCTCACCGGATTCAGTCGTCACTCATGGTGATTTCTCACTTGATAACCTTATTTTTG ACGAGGGGAAATTAATAGGTTGTATTGATGTTGGACGAGTCGGAATCGCAGACCGATACCAGGATCT TGCCATCCTATGGAACTGCCTCGGTGAGTTTTCTCCTTCATTACAGAAACGGCTTTTTCAAAAATATGG TATTGATAATCCTGATATGAATAAATTGCAGTTTCATTTGATGCTCGATGAGTTTTTCTAAGCTGTCAG ACCAAGTTTACTCATATATACTTTAGATTGATTTAAAACTTCATTTTTAATTTAAAAGGATCTAGGTGAA GATCCTTTTTGATAATCTCATGACCAAAATCCCTTAACGTGAGTTTTCGTTCCACTGAGCGTCAGACCCC GTAGAAAAGATCAAAGGATCTTCTTGAGATCCTTTTTTTCTGCGCGTAATCTGCTGCTTGCAAACAAAA AAACCACCGCTACCAGCGGTGGTTTGTTTGCCGGATCAAGAGCTACCAACTCTTTTTCCGAAGGTAAC

	TGGCTTCAGCAGAGCGCAGATACCAAATACTGTCCTTCTAGTGTAGCCGTAGTTAGGCCACCACTTCA<br>AGAACTCTGTAGCACCGCCTACATACCTCGCTCTGCTAATCCTGTTACCAGTGGCTGCTGCCAGTGGCG<br>ATAAGTCGTGTCTTACCGGGTTGGACTCAAGACGATAGTTACCGGATAAGGCGCAGCGGTCGGGCTG<br>AACGGGGGGTTCGTGCACACAGCCCAGCTTGGAGCGAACGACCTACACCGAACTGAGATACCTACAG<br>CGTGAGCTATGAGAAAGCGCCACGCTTCCCGAAGGGAGAAAGGCGGACAGGTATCCGGTAAGCGGC<br>AGGGTCGGAACAGGAGAGCGCACGAGGGAGCTTCCAGGGGGAAACGCCTGGTATCTTTATAGTCCT<br>GTCGGGTTTCGCCACCTCTGACTTGAGCGTCGATTTTTGTGATGCTCGTCAGGGGGGCGGAGCCTATG<br>GAAAAACGCCAGCAACGCGGCCTTTTTACGGTTCCTGGCCTTTTGCTGGCCTTTTGCTCACATGTTCTT<br>TCCTGCGTTATCCCCTGATTCTGTGGATAACCGTATTACCGCCTTTGAGTGAGCTGATACCGCTCGCCG<br>CAGCCGAACGACCGAGCGCAGCGAGTCAGTGAGCGAGGAAGCGGAAGAGCGCCTGATGCGGTATTT<br>TCTCCTTACGCATCTGTGCGGTATTTCACACCGCATATGGTGCACTCTCAGTACAATCTGCTCTGATGC<br>CGCATAGTTAAGCCAGTATACACTCCGCTATCGCTACGTGACTGGGTCATGGCTGCGCCCCGACACCC<br>GCCAACACCCGCTGACGCGCCCTGACGGGCTTGTCTGCTCCCGGCATCCGCTTACAGACAAGCTGTGA<br>CCGTCTCCGGGAGCTGCATGTGTCAGAGGTTTTCACCGTCATCACCGAAACGCGCGAGGCAGCAGAT<br>CAATTCGCGCGCGAAGGCGAAGCGGCATGCATAATGTGCCTGTCAAATGGACGAAGCAGGGATTCTG<br>CAAACCCTATGCTACTCCGTCAAGCCGTCAATTGTCTGATTCGTTACCAATTATGACAACTTGACGGCT<br>ACATCATTCACTTTTTCTTCACAACCGGCACGGAACTCGCTCGGGCTGGCCCCGGTGCATTTTTTAAAT<br>ACCCGCGAGAAATAGAGTTGATCGTCAAAACCAACATTGCGACCGACGGTGGCGATAGGCATCCGGG<br>TGGTGCTCAAAAGCAGCTTCGCCTGGCTGATACGTTGGTCCTCGCGCCAGCTTAAGACGCTAATCCCT<br>AACTGCTGGCGGAAAAGATGTGACAGACGCGACGGCGACAAGCAAACATGCTGTGCGACGCTGGCG<br>ATATCAAAATTGCTGTCTGCCAGGTGATCGCTGATGTACTGACAAGCCTCGCGTACCCGATTATCCATC<br>GGTGGATGGAGCGACTCGTTAATCGCTTCCATGCGCCGCAGTAACAATTGCTCAAGCAGATTTATCGC<br>CAGCAGCTCCGAATAGCGCCCTTCCCCTTGCCCGGCGTTAATGATTTGCCCAAACAGGTCGCTGAAAT<br>GCGGCTGGTGCGCTTCATCCGGGCGAAAGAACCCCGTATTGGCAAATATTGACGGCCAGTTAAGCCA<br>TTCATGCCAGTAGGCGCGCGGACGAAAGTAAACCCACTGGTGATACCATTCGCGAGCCTCCGGATGA<br>CGACCGTAGTGATGAATCTCTCCTGGCGGGAACAGCAAAATATCACCCGGTCGGCAAACAAATTCTCG<br>TCCCTGATTTTTCACCACCCCCTGACCGCGAATGGTGAGATTGAGAATATAACCTTTCATTCCCAGCGG<br>TCGGTCGATAAAAAAATCGAGATAACCGTTGGCCTCAATCGGCGTTAAACCCGCCACCAGATGGGCA<br>TTAAACGAGTATCCCGGCAGCAGGGGATCATTTTGCGCTTCAGCCATACTTTTCATACTCCCGCCATTC<br>AGAG
pBAD:pre^{MalE}-scFv-TCD-mCherry	AAGAAACCAATTGTCCATATTGCATCAGACATTGCCGTCACTGCGTCTTTTACTGGCTCTTCTCGCTAAC<br>CAAACCGGTAACCCCGCTTATTAAAAGCATTCTGTAACAAAGCGGGACCAAAGCCATGACAAAAACG<br>CGTAACAAAAGTGTCTATAATCACGGCAGAAAAGTCCACATTGATTATTTGCACGGCGTCACACTTTG<br>CTATGCCATAGCATTTTTATCCATAAGATTAGCGGATCCTACCTGACGCTTTTTATCGCAACTCTCTACT<br>GTTTCTCCATACCCGTTTTTTTGGGCTAGAAATAATTTTGTTTAACTTTAAGAAGGAGATATACATATGA<br>AAATAAAAACAGGTGCACGCATCCTCGCATTATCCGCATTAACGACGATGATGTTTTCCGCCTCGGCTC<br>TCGCCGAAGTGCAGCTGGTCGAATCGGGTGGCGGATTAGTGCAGCCTGGAGGCTCCTTACGCCTGAG<br>CTGTGCAGCGAGCGGCTTCAACATCAAGGACACCTACATACATTGGGTTCGCCAAGCTCCGGGCAAA<br>GGTCTGGAGTGGGTTGCTCGTATCTATCCCACTAATGGGTATACACGCTATGCCGATAGCGTGAAAGG<br>CCGGTTTACCATTAGCGCCGATACGAGCAAGAATACGGCGTATCTGCAGATGAACTCTCTGCGTGCCG<br>AAGATACAGCGGTCTACTACTGCTCTCGTTGGGGTGGTGACGGGTTTTATGCAATGGACTATTGGGG<br>CCAAGGAACCCTCGTGACGGTTTCCTCAGGCGGAGGTGGTAGTGGTGGCGGTGGGTCTGGCGGCGG<br>TGGGAGCGACATTCAGATGACGCAGTCACCATCGTCGTTGTCAGCGTCGGTAGGTGATCGCGTCACG<br>ATTACCTGTCGTGCATCCCAAGATGTGAACACTGCAGTAGCGTGGTACCAGCAGAAACCGGGGAAAG<br>CTCCGAAACTTCTGATTTACTCGGCGAGTTTCCTGTATAGTGGCGTTCCAAGTCGCTTTAGCGGTTCCC<br>GTTCTGGCACGGATTTCACACTGACCATCTCAAGCTTGCAGCCGGAAGATTTTGCCACCTATTACTGCC<br>AACAGCACTATACCACTCCTCCGACCTTTGGCCAAGGCACCAAAGTGGAGATCAAACGCACTAGTCAT<br>CATCACCACCATCATTAGGATGGTGGTGATGATAATGATGAGCAAGGGCGAGGAGGATAACATGGCC<br>ATCATCAAGGAGTTCATGCGCTTCAAGGTGCACATGGAGGGCTCCGTGAACGGCCACGAGTTCGAGA<br>TCGAGGGCGAGGGCGAGGGCCGCCCCTACGAGGGCACCCAGACCGCCAAGCTGAAGGTGACCAAG<br>GGTGGCCCCCTGCCCTTCGCCTGGGACATCCTGTCCCCTCAGTTCATGTACGGCTCCAAGGCCTACGT<br>GAAGCACCCCGCCGACATCCCCGACTACTTGAAGCTGTCCTTCCCCGAGGGCTTCAAGTGGGAGCGC<br>GTGATGAACTTCGAGGACGGCGGCGTGGTGACCGTGACCCAGGACTCCTCCCTGCAGGACGGCGAG<br>TTCATCTACAAGGTGAAGCTGCGCGGCACCAACTTCCCCTCCGACGGCCCCGTAATGCAGAAGAAGAC<br>CATGGGCTGGGAGGCCTCCTCCGAGCGGATGTACCCCGAGGACGGCGCCCTGAAGGGCGAGATCAA<br>GCAGAGGCTGAAGCTGAAGGACGGCGGCCACTACGACGCTGAGGTCAAGACCACCTACAAGGCCAA<br>GAAGCCCGTGCAGCTGCCCGGCGCCTACAACGTCAACATCAAGTTGGACATCACCTCCCACAACGAG<br>GACTACACCATCGTGGAACAGTACGAACGCGCCGAGGGCCGCCACTCCACCGGCGGCATGGACGAG<br>CTGTACAAGTGAAAGCTTACGGTCTCCAGCTTGGCTGTTTTGGCGGATGAGAGAAGATTTTCAGCCTG<br>ATACAGATTAAATCAGAACGCAGAAGCGGTCTGATAAAACAGAATTTGCCTGGCGGCAGTAGCGCGG<br>TGGTCCCACCTGACCCCATGCCGAACTCAGAAGTGAAACGCCGTAGCGCCGATGGTAGTGTGGGGTC<br>TCCCCATGCGAGAGTAGGGAACTGCCAGGCATCAAATAAAACGAAAGGCTCAGTCGAAAGACTGGG

	CCTTTCGTTTTATCTGTTGTTTGTCGGTGAACGCTCTCCTGAGTAGGACAAATCCGCCGGGAGCGGATT<br>TGAACGTTGCGAAGCAACGGCCCGGAGGGTGGCGGGCAGGACGCCCGCCATAAACTGCCAGGCATC<br>AAATTAAGCAGAAGGCCATCCTGACGGATGGCCTTTTTGCGTTTCTACAAACTCTCATGAACAATAAA<br>ACTGTCTGCTTACATAAACAGTAATACAAGGGGTGTTATGAGCCATATTCAACGGGAAACGTCTTGCT<br>CTAGGCCGCGATTAAATTCCAACATGGATGCTGATTTATATGGGTATAAATGGGCTCGCGATAATGTC<br>GGGCAATCAGGTGCGACAATCTATCGATTGTATGGGAAGCCCGATGCGCCAGAGTTGTTTCTGAAAC<br>ATGGCAAAGGTAGCGTTGCCAATGATGTTACAGATGAGATGGTCAGACTAAACTGGCTGACGGAATT<br>TATGCCTCTTCCGACCATCAAGCATTTTATCCGTACTCCTGATGATGCATGGTTACTCACCACTGCGATC<br>CCCGGGAAAACAGCATTCCAGGTATTAGAAGAATATCCTGATTCAGGTGAAAATATTGTTGATGCGCT<br>GGCAGTGTTCCTGCGCCGGTTGCATTCGATTCCTGTTTGTAATTGTCCTTTTAACAGCGATCGCGTATT<br>TCGTCTCGCTCAGGCGCAATCACGAATGAATAACGGTTTGGTTGATGCGAGTGATTTTGATGACGAGC<br>GTAATGGCTGGCCTGTTGAACAAGTCTGGAAAGAAATGCATAAACTTTTGCCATTCTCACCGGATTCA<br>GTCGTCACTCATGGTGATTTCTCACTTGATAACCTTATTTTTGACGAGGGGAAATTAATAGGTTGTATT<br>GATGTTGGACGAGTCGGAATCGCAGACCGATACCAGGATCTTGCCATCCTATGGAACTGCCTCGGTG<br>AGTTTTCTCCTTCATTACAGAAACGGCTTTTTCAAAAATATGGTATTGATAATCCTGATATGAATAAATT<br>GCAGTTTCATTTGATGCTCGATGAGTTTTTCTAAGCTGTCAGACCAAGTTTACTCATATATACTTTAGAT<br>TGATTTAAAACTTCATTTTTAATTTAAAAGGATCTAGGTGAAGATCCTTTTTGATAATCTCATGACCAAA<br>ATCCCTTAACGTGAGTTTTCGTTCCACTGAGCGTCAGACCCCGTAGAAAAGATCAAAGGATCTTCTTGA<br>GATCCTTTTTTTCTGCGCGTAATCTGCTGCTTGCAAACAAAAAAACCACCGCTACCAGCGGTGGTTTGT<br>TTGCCGGATCAAGAGCTACCAACTCTTTTTCCGAAGGTAACTGGCTTCAGCAGAGCGCAGATACCAAA<br>TACTGTCCTTCTAGTGTAGCCGTAGTTAGGCCACCACTTCAAGAACTCTGTAGCACCGCCTACATACCT<br>CGCTCTGCTAATCCTGTTACCAGTGGCTGCTGCCAGTGGCGATAAGTCGTGTCTTACCGGGTTGGACT<br>CAAGACGATAGTTACCGGATAAGGCGCAGCGGTCGGGCTGAACGGGGGGTTCGTGCACACAGCCCA<br>GCTTGGAGCGAACGACCTACACCGAACTGAGATACCTACAGCGTGAGCTATGAGAAAGCGCCACGCT<br>TCCCGAAGGGAGAAAGGCGGACAGGTATCCGGTAAGCGGCAGGGTCGGAACAGGAGAGCGCACGA<br>GGGAGCTTCCAGGGGGAAACGCCTGGTATCTTTATAGTCCTGTCGGGTTTCGCCACCTCTGACTTGAG<br>CGTCGATTTTTGTGATGCTCGTCAGGGGGGCGGAGCCTATGGAAAAACGCCAGCAACGCGGCCTTTT<br>TACGGTTCCTGGCCTTTTGCTGGCCTTTTGCTCACATGTTCTTTCCTGCGTTATCCCCTGATTCTGTGGA<br>TAACCGTATTACCGCCTTTGAGTGAGCTGATACCGCTCGCCGCAGCCGAACGACCGAGCGCAGCGAG<br>TCAGTGAGCGAGGAAGCGGAAGAGCGCCTGATGCGGTATTTTCTCCTTACGCATCTGTGCGGTATTTC<br>ACACCGCATATGGTGCACTCTCAGTACAATCTGCTCTGATGCCGCATAGTTAAGCCAGTATACACTCCG<br>CTATCGCTACGTGACTGGGTCATGGCTGCGCCCCGACACCCGCCAACACCCGCTGACGCGCCCTGACG<br>GGCTTGTCTGCTCCCGGCATCCGCTTACAGACAAGCTGTGACCGTCTCCGGGAGCTGCATGTGTCAGA<br>GGTTTTCACCGTCATCACCGAAACGCGCGAGGCAGCAGATCAATTCGCGCGCGAAGGCGAAGCGGCA<br>TGCATAATGTGCCTGTCAAATGGACGAAGCAGGGATTCTGCAAACCCTATGCTACTCCGTCAAGCCGT<br>CAATTGTCTGATTCGTTACCAATTATGACAACTTGACGGCTACATCATTCACTTTTTCTTCACAACCGGC<br>ACGGAACTCGCTCGGGCTGGCCCCGGTGCATTTTTTAAATACCCGCGAGAAATAGAGTTGATCGTCAA<br>AACCAACATTGCGACCGACGGTGGCGATAGGCATCCGGGTGGTGCTCAAAAGCAGCTTCGCCTGGCT<br>GATACGTTGGTCCTCGCGCCAGCTTAAGACGCTAATCCCTAACTGCTGGCGGAAAAGATGTGACAGA<br>CGCGACGGCGACAAGCAAACATGCTGTGCGACGCTGGCGATATCAAAATTGCTGTCTGCCAGGTGAT<br>CGCTGATGTACTGACAAGCCTCGCGTACCCGATTATCCATCGGTGGATGGAGCGACTCGTTAATCGCT<br>TCCATGCGCCGCAGTAACAATTGCTCAAGCAGATTTATCGCCAGCAGCTCCGAATAGCGCCCTTCCCCT<br>TGCCCGGCGTTAATGATTTGCCCAAACAGGTCGCTGAAATGCGGCTGGTGCGCTTCATCCGGGCGAA<br>AGAACCCCGTATTGGCAAATATTGACGGCCAGTTAAGCCATTCATGCCAGTAGGCGCGCGGACGAAA<br>GTAAACCCACTGGTGATACCATTCGCGAGCCTCCGGATGACGACCGTAGTGATGAATCTCTCCTGGCG<br>GGAACAGCAAAATATCACCCGGTCGGCAAACAAATTCTCGTCCCTGATTTTTCACCACCCCCTGACCGC<br>GAATGGTGAGATTGAGAATATAACCTTTCATTCCCAGCGGTCGGTCGATAAAAAAATCGAGATAACC<br>GTTGGCCTCAATCGGCGTTAAACCCGCCACCAGATGGGCATTAAACGAGTATCCCGGCAGCAGGGGA<br>TCATTTTGCGCTTCAGCCATACTTTTCATACTCCCGCCATTCAGAG
pBAD:pre^{DsbA}-scFv-TCD-mCherry	AAGAAACCAATTGTCCATATTGCATCAGACATTGCCGTCACTGCGTCTTTTACTGGCTCTTCTCGCTAAC<br>CAAACCGGTAACCCCGCTTATTAAAAGCATTCTGTAACAAAGCGGGACCAAAGCCATGACAAAAACG<br>CGTAACAAAAGTGTCTATAATCACGGCAGAAAAGTCCACATTGATTATTTGCACGGCGTCACACTTTG<br>CTATGCCATAGCATTTTTATCCATAAGATTAGCGGATCCTACCTGACGCTTTTTATCGCAACTCTCTACT<br>GTTTCTCCATACCCGAATAATTTTGTTTAACTTTAAGAAGGAGATTAACGTATGAAAAAGATTTGGCTG<br>GCGCTGGCTGGTTTAGTTTTAGCGTTTAGCGCATCGGCGGAAGTGCAGCTGGTCGAATCGGGTGGCG<br>GATTAGTGCAGCCTGGAGGCTCCTTACGCCTGAGCTGTGCAGCGAGCGGCTTCAACATCAAGGACAC<br>CTACATACATTGGGTTCGCCAAGCTCCGGGCAAAGGTCTGGAGTGGGTTGCTCGTATCTATCCCACTA<br>ATGGGTATACACGCTATGCCGATAGCGTGAAAGGCCGGTTTACCATTAGCGCCGATACGAGCAAGAA<br>TACGGCGTATCTGCAGATGAACTCTCTGCGTGCCGAAGATACAGCGGTCTACTACTGCTCTCGTTGGG<br>GTGGTGACGGGTTTTATGCAATGGACTATTGGGGCCAAGGAACCCTCGTGACGGTTTCCTCAGGCGG<br>AGGTGGTAGTGGTGGCGGTGGGTCTGGCGGCGGTGGGAGCGACATTCAGATGACGCAGTCACCATC<br>GTCGTTGTCAGCGTCGGTAGGTGATCGCGTCACGATTACCTGTCGTGCATCCCAAGATGTGAACACTG

CAGTAGCGTGGTACCAGCAGAAACCGGGGAAAGCTCCGAAACTTCTGATTTACTCGGCGAGTTTCCT
GTATAGTGGCGTTCCAAGTCGCTTTAGCGGTTCCCGTTCTGGCACGGATTTCACACTGACCATCTCAAG
CTTGCAGCCGGAAGATTTTGCCACCTATTACTGCCAACAGCACTATACCACTCCTCCGACCTTTGGCCA
AGGCACCAAAGTGGAGATCAAACGCACTAGTCATCATCACCACCATCATTAGGATGGTGGTGATGAT
AATGATGAGCAAGGGCGAGGAGGATAACATGGCCATCATCAAGGAGTTCATGCGCTTCAAGGTGCA
CATGGAGGGCTCCGTGAACGGCCACGAGTTCGAGATCGAGGGCGAGGGCGAGGGCCGCCCCTACGA
GGGCACCCAGACCGCCAAGCTGAAGGTGACCAAGGGTGGCCCCCTGCCCTTCGCCTGGGACATCCTG
TCCCCTCAGTTCATGTACGGCTCCAAGGCCTACGTGAAGCACCCCGCCGACATCCCCGACTACTTGAA
GCTGTCCTTCCCCGAGGGCTTCAAGTGGGAGCGCGTGATGAACTTCGAGGACGGCGGCGTGGTGACC
GTGACCCAGGACTCCTCCCTGCAGGACGGCGAGTTCATCTACAAGGTGAAGCTGCGCGGCACCAACT
TCCCCTCCGACGGCCCCGTAATGCAGAAGAAGACCATGGGCTGGGAGGCCTCCTCCGAGCGGATGTA
CCCCGAGGACGGCGCCCTGAAGGGCGAGATCAAGCAGAGGCTGAAGCTGAAGGACGGCGGCCACT
ACGACGCTGAGGTCAAGACCACCTACAAGGCCAAGAAGCCCGTGCAGCTGCCCGGCGCCTACAACGT
CAACATCAAGTTGGACATCACCTCCCACAACGAGGACTACACCATCGTGGAACAGTACGAACGCGCC
GAGGGCCGCCACTCCACCGGCGGCATGGACGAGCTGTACAAGTAACATATGGCTAGCATGACTGGTG
GACAGCAAATGGCCAGGATACGCTTGGCTGTTTTGGCGGATGAGAGAAGATTTTCAGCCTGATACAG
ATTAAATCAGAACGCAGAAGCGGTCTGATAAAACAGAATTTGCCTGGCGGCAGTAGCGCGGTGGTCC
CACCTGACCCCATGCCGAACTCAGAAGTGAAACGCCGTAGCGCCGATGGTAGTGTGGGGTCTCCCCA
TGCGAGAGTAGGGAACTGCCAGGCATCAAATAAAACGAAAGGCTCAGTCGAAAGACTGGGCCTTTCG
TTTTATCTGTTGTTTGTCGGTGAACGCTCTCCTGAGTAGGACAAATCCGCCGGGAGCGGATTTGAACG
TTGCGAAGCAACGGCCCGGAGGGTGGCGGGCAGGACGCCCGCCATAAACTGCCAGGCATCAAATTA
AGCAGAAGGCCATCCTGACGGATGGCCTTTTTGCGTTTCTACAAACTCTCATGAACAATAAAACTGTCT
GCTTACATAAACAGTAATACAAGGGGTGTTATGAGCCATATTCAACGGGAAACGTCTTGCTCTAGGCC
GCGATTAAATTCCAACATGGATGCTGATTTATATGGGTATAAATGGGCTCGCGATAATGTCGGGCAAT
CAGGTGCGACAATCTATCGATTGTATGGGAAGCCCGATGCGCCAGAGTTGTTTCTGAAACATGGCAA
AGGTAGCGTTGCCAATGATGTTACAGATGAGATGGTCAGACTAAACTGGCTGACGGAATTTATGCCTC
TTCCGACCATCAAGCATTTTATCCGTACTCCTGATGATGCATGGTTACTCACCACTGCGATCCCCGGGA
AAACAGCATTCCAGGTATTAGAAGAATATCCTGATTCAGGTGAAAATATTGTTGATGCGCTGGCAGTG
TTCCTGCGCCGGTTGCATTCGATTCCTGTTTGTAATTGTCCTTTTAACAGCGATCGCGTATTTCGTCTCG
CTCAGGCGCAATCACGAATGAATAACGGTTTGGTTGATGCGAGTGATTTTGATGACGAGCGTAATGG
CTGGCCTGTTGAACAAGTCTGGAAAGAAATGCATAAACTTTTGCCATTCTCACCGGATTCAGTCGTCAC
TCATGGTGATTTCTCACTTGATAACCTTATTTTTGACGAGGGGAAATTAATAGGTTGTATTGATGTTGG
ACTAGTCGGAATCGCAGACCGATACCAGGATCTTGCCATCCTATGGAACTGCCTCGGTGAGTTTTCTC
CTTCATTACAGAAACGGCTTTTTCAAAAATATGGTATTGATAATCCTGATATGAATAAATTGCAGTTTC
ATTTGATGCTCGATGAGTTTTTCTAAGCTGTCAGACCAAGTTTACTCATATATACTTTAGATTGATTTAC
GCGCCCTGTAGCGGCGCATTAAGCGCGGCGGGTGTGGTGGTTACGCGCAGCGTGACCGCTACACTTG
CCAGCGCCCTAGCGCCCGCTCCTTTCGCTTTCTTCCCTTCCTTTCTCGCCACGTTCGCCGGCTTTCCCCGT
CAAGCTCTAAATCGGGGGCTCCCTTTAGGGTTCCGATTTAGTGCTTTACGGCACCTCGACCCCAAAAA
ACTTGATTTGGGTGATGGTTCACGTAGTGGGCCATCGCCCTGATAGACGGTTTTTCGCCCTTTGACGTT
GGAGTCCACGTTCTTTAATAGTGGACTCTTGTTCCAAACTGGAACAACACTCAACCCTATCTCGGGCTA
TTCTTTTGATTTATAAGGGATTTTGCCGATTTCGGCCTATTGGTTAAAAAATGAGCTGATTTAACAAAA
ATTTAACGCGAATTTTAACAAAATATTAACGTTTACAATTTAAAAGGATCTAGGTGAAGATCCTTTTTG
ATAATCTCATGACCAAAATCCCTTAACGTGAGTTTTCGTTCCACTGAGCGTCAGACCCCGTAGAAAAG
ATCAAAGGATCTTCTTGAGATCCTTTTTTTCTGCGCGTAATCTGCTGCTTGCAAACAAAAAAACCACCG
CTACCAGCGGTGGTTTGTTTGCCGGATCAAGAGCTACCAACTCTTTTTCCGAAGGTAACTGGCTTCAGC
AGAGCGCAGATACCAAATACTGTCCTTCTAGTGTAGCCGTAGTTAGGCCACCACTTCAAGAACTCTGT
AGCACCGCCTACATACCTCGCTCTGCTAATCCTGTTACCAGTGGCTGCTGCCAGTGGCGATAAGTCGT
GTCTTACCGGGTTGGACTCAAGACGATAGTTACCGGATAAGGCGCAGCGGTCGGGCTGAACGGGGG
GTTCGTGCACACAGCCCAGCTTGGAGCGAACGACCTACACCGAACTGAGATACCTACAGCGTGAGCT
ATGAGAAAGCGCCACGCTTCCCGAAGGGAGAAAGGCGGACAGGTATCCGGTAAGCGGCAGGGTCG
GAACAGGAGAGCGCACGAGGGAGCTTCCAGGGGGAAACGCCTGGTATCTTTATAGTCCTGTCGGGTT
TCGCCACCTCTGACTTGAGCGTCGATTTTTGTGATGCTCGTCAGGGGGGCGGAGCCTATGGAAAAAC
GCCAGCAACGCGGCCTTTTTACGGTTCCTGGCCTTTTGCTGGCCTTTTGCTCACATGTTCTTTCCTGCGT
TATCCCCTGATTCTGTGGATAACCGTATTACCGCCTTTGAGTGAGCTGATACCGCTCGCCGCAGCCGAA
CGACCGAGCGCAGCGAGTCAGTGAGCGAGGAAGCGGAAGAGCGCCTGATGCGGTATTTTCTCCTTAC
GCATCTGTGCGGTATTTCACACCGCATAGGGTCATGGCTGCGCCCCGACACCCGCCAACACCCGCTGA
CGCGCCCTGACGGGCTTGTCTGCTCCCGGCATCCGCTTACAGACAAGCTGTGACCGTCTCCGGGAGCT
GCATGTGTCAGAGGTTTTCACCGTCATCACCGAAACGCGCGAGGCAGAAGGAGATGGCGCCCAACAG
TCCCCCGGCCACGGGGCCTGCCACCATACCCACGCCGAAACAAGCGCTCATGAGCCCGAAGTGGCGA
GCCCGATCTTCCCCATCGGTGATGTCGGCGATATAGGCGCCAGCAACCGCACCTGTGGCGCCGGTGA
TGCCGGCCACGATGCGTCCGGCGTAGAGGATCTGCTCATGTTTGACAGCTTATCATCGATGCATAATG
TGCCTGTCAAATGGACGAAGCAGGGATTCTGCAAACCCTATGCTACTCCGTCAAGCCGTCAATTGTCT
GATTCGTTACCAATTATGACAACTTGACGGCTACATCATTCACTTTTTCTTCACAACCGGCACGGAACT

<table>
<tr><td></td><td>CGCTCGGGCTGGCCCCGGTGCATTTTTTAAATACCCGCGAGAAATAGAGTTGATCGTCAAAACCAACA
TTGCGACCGACGGTGGCGATAGGCATCCGGGTGGTGCTCAAAAGCAGCTTCGCCTGGCTGATACGTT
GGTCCTCGCGCCAGCTTAAGACGCTAATCCCTAACTGCTGGCGGAAAAGATGTGACAGACGCGACGG
CGACAAGCAAACATGCTGTGCGACGCTGGCGATATCAAAATTGCTGTCTGCCAGGTGATCGCTGATGT
ACTGACAAGCCTCGCGTACCCGATTATCCATCGGTGGATGGAGCGACTCGTTAATCGCTTCCATGCGC
CGCAGTAACAATTGCTCAAGCAGATTTATCGCCAGCAGCTCCGAATAGCGCCCTTCCCCTTGCCCGGC
GTTAATGATTTGCCCAAACAGGTCGCTGAAATGCGGCTGGTGCGCTTCATCCGGGCGAAAGAACCCC
GTATTGGCAAATATTGACGGCCAGTTAAGCCATTCATGCCAGTAGGCGCGCGGACGAAAGTAAACCC
ACTGGTGATACCATTCGCGAGCCTCCGGATGACGACCGTAGTGATGAATCTCTCCTGGCGGGAACAG
CAAAATATCACCCGGTCGGCAAACAAATTCTCGTCCCTGATTTTTCACCACCCCCTGACCGCGAATGGT
GAGATTGAGAATATAACCTTTCATTCCCAGCGGTCGGTCGATAAAAAAATCGAGATAACCGTTGGCCT
CAATCGGCGTTAAACCCGCCACCAGATGGGCATTAAACGAGTATCCCGGCAGCAGGGGATCATTTTG
CGCTTCAGCCATACTTTTCATACTCCCGCCATTCAGAG</td></tr>
<tr><td>pBAD:Δss-scFv-TCD-mCherry</td><td>AAGAAACCAATTGTCCATATTGCATCAGACATTGCCGTCACTGCGTCTTTTACTGGCTCTTCTCGCTAAC
CAAACCGGTAACCCCGCTTATTAAAAGCATTCTGTAACAAAGCGGGACCAAAGCCATGACAAAAACG
CGTAACAAAAGTGTCTATAATCACGGCAGAAAAGTCCACATTGATTATTTGCACGGCGTCACACTTTG
CTATGCCATAGCATTTTTATCCATAAGATTAGCGGATCCTACCTGACGCTTTTTATCGCAACTCTCTACT
GTTTCTCCATACCCGTTTTTTTGGGCTAGAAATAATTTTGTTTAACTTTAAGAAGGAGATATACATATG
GAAGTGCAGCTGGTCGAATCGGGTGGCGGATTAGTGCAGCCTGGAGGCTCCTTACGCCTGAGCTGTG
CAGCGAGCGGCTTCAACATCAAGGACACCTACATACATTGGGTTCGCCAAGCTCCGGGCAAAGGTCT
GGAGTGGGTTGCTCGTATCTATCCCACTAATGGGTATACACGCTATGCCGATAGCGTGAAAGGCCGG
TTTACCATTAGCGCCGATACGAGCAAGAATACGGCGTATCTGCAGATGAACTCTCTGCGTGCCGAAGA
TACAGCGGTCTACTACTGCTCTCGTTGGGGTGGTGACGGGTTTTATGCAATGGACTATTGGGGCCAAG
GAACCCTCGTGACGGTTTCCTCAGGCGGAGGTGGTAGTGGTGGCGGTGGGTCTGGCGGCGGTGGGA
GCGACATTCAGATGACGCAGTCACCATCGTCGTTGTCAGCGTCGGTAGGTGATCGCGTCACGATTACC
TGTCGTGCATCCCAAGATGTGAACACTGCAGTAGCGTGGTACCAGCAGAAACCGGGGAAAGCTCCGA
AACTTCTGATTTACTCGGCGAGTTTCCTGTATAGTGGCGTTCCAAGTCGCTTTAGCGGTTCCCGTTCTG
GCACGGATTTCACACTGACCATCTCAAGCTTGCAGCCGGAAGATTTTGCCACCTATTACTGCCAACAGC
ACTATACCACTCCTCCGACCTTTGGCCAAGGCACCAAAGTGGAGATCAAACGC<u>ACTAGTCATCATCAC</u>
<u>CACCATCATTAG</u><u>GATGGTGGTGATGATA</u>ATGATGAGCAAGGGCGAGGAGGATAACATGGCCATCATC
AAGGAGTTCATGCGCTTCAAGGTGCACATGGAGGGCTCCGTGAACGGCCACGAGTTCGAGATCGAG
GGCGAGGGCGAGGGCCGCCCCTACGAGGGCACCCAGACCGCCAAGCTGAAGGTGACCAAGGGTGG
CCCCCTGCCCTTCGCCTGGGACATCCTGTCCCCTCAGTTCATGTACGGCTCCAAGGCCTACGTGAAGCA
CCCCGCCGACATCCCCGACTACTTGAAGCTGTCCTTCCCCGAGGGCTTCAAGTGGGAGCGCGTGATGA
ACTTCGAGGACGGCGGCGTGGTGACCGTGACCCAGGACTCCTCCCTGCAGGACGGCGAGTTCATCTA
CAAGGTGAAGCTGCGCGGCACCAACTTCCCCTCCGACGGCCCCGTAATGCAGAAGAAGACCATGGGC
TGGGAGGCCTCCTCCGAGCGGATGTACCCCGAGGACGGCGCCCTGAAGGGCGAGATCAAGCAGAGG
CTGAAGCTGAAGGACGGCGGCCACTACGACGCTGAGGTCAAGACCACCTACAAGGCCAAGAAGCCC
GTGCAGCTGCCCGGCGCCTACAACGTCAACATCAAGTTGGACATCACCTCCCACAACGAGGACTACAC
CATCGTGGAACAGTACGAACGCGCCGAGGGCCGCCACTCCACCGGCGGCATGGACGAGCTGTACAA
GTGAAAGCTTACGGTCTCCAGCTTGGCTGTTTTGGCGGATGAGAGAAGATTTTCAGCCTGATACAGAT
TAAATCAGAACGCAGAAGCGGTCTGATAAAACAGAATTTGCCTGGCGGCAGTAGCGCGGTGGTCCCA
CCTGACCCCATGCCGAACTCAGAAGTGAAACGCCGTAGCGCCGATGGTAGTGTGGGGTCTCCCCATG
CGAGAGTAGGGAACTGCCAGGCATCAAATAAAACGAAAGGCTCAGTCGAAAGACTGGGCCTTTCGTT
TTATCTGTTGTTTGTCGGTGAACGCTCTCCTGAGTAGGACAAATCCGCCGGGAGCGGATTTGAACGTT
GCGAAGCAACGGCCCGGAGGGTGGCGGGCAGGACGCCCGCCATAAACTGCCAGGCATCAAATTAAG
CAGAAGGCCATCCTGACGGATGGCCTTTTTGCGTTTCTACAAACTCTCATGAACAATAAAACTGTCTGC
TTACATAAACAGTAATACAAGGGGTGTTATGAGCCATATTCAACGGGAAACGTCTTGCTCTAGGCCGC
GATTAAATTCCAACATGGATGCTGATTTATATGGGTATAAATGGGCTCGCGATAATGTCGGGCAATCA
GGTGCGACAATCTATCGATTGTATGGGAAGCCCGATGCGCCAGAGTTGTTTCTGAAACATGGCAAAG
GTAGCGTTGCCAATGATGTTACAGATGAGATGGTCAGACTAAACTGGCTGACGGAATTTATGCCTCTT
CCGACCATCAAGCATTTTATCCGTACTCCTGATGATGCATGGTTACTCACCACTGCGATCCCCGGGAAA
ACAGCATTCCAGGTATTAGAAGAATATCCTGATTCAGGTGAAAATATTGTTGATGCGCTGGCAGTGTT
CCTGCGCCGGTTGCATTCGATTCCTGTTTGTAATTGTCCTTTTAACAGCGATCGCGTATTTCGTCTCGCT
CAGGCGCAATCACGAATGAATAACGGTTTGGTTGATGCGAGTGATTTTGATGACGAGCGTAATGGCT
GGCCTGTTGAACAAGTCTGGAAAGAAATGCATAAACTTTTGCCATTCTCACCGGATTCAGTCGTCACTC
ATGGTGATTTCTCACTTGATAACCTTATTTTTGACGAGGGGAAATTAATAGGTTGTATTGATGTTGGAC
GAGTCGGAATCGCAGACCGATACCAGGATCTTGCCATCCTATGGAACTGCCTCGGTGAGTTTTCTCCT
TCATTACAGAAACGGCTTTTTCAAAAATATGGTATTGATAATCCTGATATGAATAAATTGCAGTTTCAT
TTGATGCTCGATGAGTTTTTCTAAGCTGTCAGACCAAGTTTACTCATATATACTTTAGATTGATTTAAAA
CTTCATTTTTAATTTAAAAGGATCTAGGTGAAGATCCTTTTTGATAATCTCATGACCAAAATCCCTTAAC
GTGAGTTTTCGTTCCACTGAGCGTCAGACCCCGTAGAAAAGATCAAAGGATCTTCTTGAGATCCTTTTT</td></tr>
</table>

	TTCTGCGCGTAATCTGCTGCTTGCAAACAAAAAAACCACCGCTACCAGCGGTGGTTTGTTTGCCGGAT<br>CAAGAGCTACCAACTCTTTTTCCGAAGGTAACTGGCTTCAGCAGAGCGCAGATACCAAATACTGTCCT<br>TCTAGTGTAGCCGTAGTTAGGCCACCACTTCAAGAACTCTGTAGCACCGCCTACATACCTCGCTCTGCT<br>AATCCTGTTACCAGTGGCTGCTGCCAGTGGCGATAAGTCGTGTCTTACCGGGTTGGACTCAAGACGAT<br>AGTTACCGGATAAGGCGCAGCGGTCGGGCTGAACGGGGGGTTCGTGCACACAGCCCAGCTTGGAGC<br>GAACGACCTACACCGAACTGAGATACCTACAGCGTGAGCTATGAGAAAGCGCCACGCTTCCCGAAGG<br>GAGAAAGGCGGACAGGTATCCGGTAAGCGGCAGGGTCGGAACAGGAGAGCGCACGAGGGAGCTTC<br>CAGGGGGAAACGCCTGGTATCTTTATAGTCCTGTCGGGTTTCGCCACCTCTGACTTGAGCGTCGATTTT<br>TGTGATGCTCGTCAGGGGGGCGGAGCCTATGGAAAAACGCCAGCAACGCGGCCTTTTTACGGTTCCT<br>GGCCTTTTGCTGGCCTTTTGCTCACATGTTCTTTCCTGCGTTATCCCCTGATTCTGTGGATAACCGTATT<br>ACCGCCTTTGAGTGAGCTGATACCGCTCGCCGCAGCCGAACGACCGAGCGCAGCGAGTCAGTGAGCG<br>AGGAAGCGGAAGAGCGCCTGATGCGGTATTTTCTCCTTACGCATCTGTGCGGTATTTCACACCGCATA<br>TGGTGCACTCTCAGTACAATCTGCTCTGATGCCGCATAGTTAAGCCAGTATACACTCCGCTATCGCTAC<br>GTGACTGGGTCATGGCTGCGCCCCGACACCCGCCAACACCCGCTGACGCGCCCTGACGGGCTTGTCT<br>GCTCCCGGCATCCGCTTACAGACAAGCTGTGACCGTCTCCGGGAGCTGCATGTGTCAGAGGTTTTCAC<br>CGTCATCACCGAAACGCGCGAGGCAGCAGATCAATTCGCGCGCGAAGGCGAAGCGGCATGCATAAT<br>GTGCCTGTCAAATGGACGAAGCAGGGATTCTGCAAACCCTATGCTACTCCGTCAAGCCGTCAATTGTC<br>TGATTCGTTACCAATTATGACAACTTGACGGCTACATCATTCACTTTTTCTTCACAACCGGCACGGAACT<br>CGCTCGGGCTGGCCCCGGTGCATTTTTTAAATACCCGCGAGAAATAGAGTTGATCGTCAAAACCAACA<br>TTGCGACCGACGGTGGCGATAGGCATCCGGGTGGTGCTCAAAAGCAGCTTCGCCTGGCTGATACGTT<br>GGTCCTCGCGCCAGCTTAAGACGCTAATCCCTAACTGCTGGCGGAAAAGATGTGACAGACGCGACGG<br>CGACAAGCAAACATGCTGTGCGACGCTGGCGATATCAAAATTGCTGTCTGCCAGGTGATCGCTGATGT<br>ACTGACAAGCCTCGCGTACCCGATTATCCATCGGTGGATGGAGCGACTCGTTAATCGCTTCCATGCGC<br>CGCAGTAACAATTGCTCAAGCAGATTTATCGCCAGCAGCTCCGAATAGCGCCCTTCCCCTTGCCCGGC<br>GTTAATGATTTGCCCAAACAGGTCGCTGAAATGCGGCTGGTGCGCTTCATCCGGGCGAAAGAACCCC<br>GTATTGGCAAATATTGACGGCCAGTTAAGCCATTCATGCCAGTAGGCGCGCGGACGAAAGTAAACCC<br>ACTGGTGATACCATTCGCGAGCCTCCGGATGACGACCGTAGTGATGAATCTCTCCTGGCGGGAACAG<br>CAAAATATCACCCGGTCGGCAAACAAATTCTCGTCCCTGATTTTTCACCACCCCCTGACCGCGAATGGT<br>GAGATTGAGAATATAACCTTTCATTCCCAGCGGTCGGTCGATAAAAAAATCGAGATAACCGTTGGCCT<br>CAATCGGCGTTAAACCCGCCACCAGATGGGCATTAAACGAGTATCCCGGCAGCAGGGGATCATTTTG<br>CGCTTCAGCCATACTTTTCATACTCCCGCCATTCAGAG
pBAD:Mth1-TCD-<br>mScarlet-I3	AAGAAACCAATTGTCCATATTGCATCAGACATTGCCGTCACTGCGTCTTTTACTGGCTCTTCTCGCTAAC<br>CAAACCGGTAACCCCGCTTATTAAAAGCATTCTGTAACAAAGCGGGACCAAAGCCATGACAAAAACG<br>CGTAACAAAAGTGTCTATAATCACGGCAGAAAAGTCCACATTGATTATTTGCACGGCGTCACACTTTG<br>CTATGCCATAGCATTTTTATCCATAAGATTAGCGGATCCTACCTGACGCTTTTTATCGCAACTCTCTACT<br>GTTTCTCCATACCCGTTTTTTTGGGCTAGAAATAATTTTGTTTAACTTTAAGAAGGAGATATACATATG<br>GGTGCAAGCCGTCTGTATACCCTGGTTCTGGTGCTGCAGCCGCAACGTGTTCTGCTGGGTATGAAAAA<br>ACGTGGTTTTGGTGCAGGTCGTTGGAATGGTTTTGGTGGTAAAGTTCAGGAAGGCGAAACCATTGAA<br>GATGGTGCACGTCGTGAACTGCAGGAAGAAAGCGGTCTGACCGTTGATGCACTGCATAAAGTTGGCC<br>AGATTGTGTTTGAATTTGTGGGTGAACCGGAACTGATGGATGTTCATGTGTTTTGCACCGATAGCATT<br>CAGGGTACACCGGTTGAATCTGATGAAATGCGTCCGTGTTGGTTTCAGCTGGATCAGATTCCGTTTAA<br>AGATATGTGGCCTGATGATAGCTATTGGTTTCCGCTGCTGCTGCAGAAAAAGAAATTCCATGGCTACT<br>TCAAATTTCAGGGCCAGGATACCATTCTGGATTATACCCTGCGTGAAGTTGATACCGTGACTAGTCATC<br>ATCACCACCATCATTAGGATGGTGGTGATGATAATGGATAGCACCGAGGCAGTGATCAAGGAGTTCA<br>TGCGGTTCAAGGTGCACATGGAGGGCTCCATGAACGGCCACGAGTTCGAGATCGAGGGCGAGGGCG<br>AGGGCCGCCCCTACGAGGGCACCCAGACCGCCAAGCTGAAGGTGACCAAGGGTGGCCCCCTGCCCTT<br>CTCCTGGGACATCCTGTCCCCTCAGTTCATGTACGGCTCCAGGGCCTTCATCAAGCACCCCGCCGACAT<br>CCCCGACTACTGGAAGCAGTCCTTCCCCGAGGGCTTCAAGTGGGAGCGCGTGATGATCTTCGAGGAC<br>GGCGGCACCGTGTCCGTGACCCAGGACACCTCCCTGGAGGACGGCACCCTGATCTACAAGGTGAAGC<br>TCCGCGGCGGCAACTTCCCTCCTGACGGCCCCGTAATGCAGAAGAGGACAATGGGCTGGGAAGCATC<br>CACCGAGCGGTTGTACCCCGAGGACGTCGTGCTGAAGGGCGACATTAAGATGGCCCTGCGCCTGAAG<br>GACGGCGGCCGCTACCTGGCGGACTTCAAGACCACCTACAAGGCCAAGAAGCCCGTGCAGATGCCCG<br>GCGCCTTCAACATCGACCGCAAGTTGGACATCACCTCCCACAACGAGGACTACACCGTGGTGGAACA<br>GTACGAACGCTCCGTGGCCCGCCACTCCACCGGCGGCTCCGGTGGCTCCTAAAAGCTTACGGTCTCCA<br>GCTTGGCTGTTTTGGCGGATGAGAGAAGATTTTCAGCCTGATACAGATTAAATCAGAACGCAGAAGC<br>GGTCTGATAAAACAGAATTTGCCTGGCGGCAGTAGCGCGGTGGTCCCACCTGACCCCATGCCGAACT<br>CAGAAGTGAAACGCCGTAGCGCCGATGGTAGTGTGGGGTCTCCCCATGCGAGAGTAGGGAACTGCC<br>AGGCATCAAATAAAACGAAAGGCTCAGTCGAAAGACTGGGCCTTTCGTTTTATCTGTTGTTTGTCGGT<br>GAACGCTCTCCTGAGTAGGACAAATCCGCCGGGAGCGGATTTGAACGTTGCGAAGCAACGGCCCGG<br>AGGGTGGCGGGCAGGACGCCCGCCATAAACTGCCAGGCATCAAATTAAGCAGAAGGCCATCCTGAC<br>GGATGGCCTTTTTGCGTTTCTACAAACTCTCATGAACAATAAAACTGTCTGCTTACATAAACAGTAATA<br>CAAGGGGTGTTATGAGCCATATTCAACGGGAAACGTCTTGCTCTAGGCCGCGATTAAATTCCAACATG

	GATGCTGATTTATATGGTATAAATGGGCTCGCGATAATGTCGGGCAATCAGGTGCGACAATCTATCG<br>ATTGTATGGGAAGCCCGATGCGCCAGAGTTGTTTCTGAAACATGGCAAAGGTAGCGTTGCCAATGAT<br>GTTACAGATGAGATGGTCAGACTAAACTGGCTGACGGAATTTATGCCTCTTCCGACCATCAAGCATTT<br>TATCCGTACTCCTGATGATGCATGGTTACTCACCACTGCGATCCCCGGGAAAACAGCATTCCAGGTATT<br>AGAAGAATATCCTGATTCAGGTGAAAATATTGTTGATGCGCTGGCAGTGTTCCTGCGCCGGTTGCATT<br>CGATTCCTGTTTGTAATTGTCCTTTTAACAGCGATCGCGTATTTCGTCTCGCTCAGGCGCAATCACGAA<br>TGAATAACGGTTTGGTTGATGCGAGTGATTTTGATGACGAGCGTAATGGCTGGCCTGTTGAACAAGTC<br>TGGAAAGAAATGCATAAACTTTTGCCATTCTCACCGGATTCAGTCGTCACTCATGGTGATTTCTCACTT<br>GATAACCTTATTTTTGACGAGGGGAAATTAATAGGTTGTATTGATGTTGGACGAGTCGGAATCGCAGA<br>CCGATACCAGGATCTTGCCATCCTATGGAACTGCCTCGGTGAGTTTTCTCCTTCATTACAGAAACGGCT<br>TTTTCAAAAATATGGTATTGATAATCCTGATATGAATAAATTGCAGTTTCATTTGATGCTCGATGAGTTT<br>TTCTAAGCTGTCAGACCAAGTTTACTCATATATACTTTAGATTGATTTAAAACTTCATTTTTAATTTAAA<br>AGGATCTAGGTGAAGATCCTTTTTGATAATCTCATGACCAAAATCCCTTAACGTGAGTTTTCGTTCCAC<br>TGAGCGTCAGACCCCGTAGAAAAGATCAAAGGATCTTCTTGAGATCCTTTTTTTCTGCGCGTAATCTGC<br>TGCTTGCAAACAAAAAAACCACCGCTACCAGCGGTGGTTTGTTTGCCGGATCAAGAGCTACCAACTCT<br>TTTTCCGAAGGTAACTGGCTTCAGCAGAGCGCAGATACCAAATACTGTCCTTCTAGTGTAGCCGTAGT<br>TAGGCCACCACTTCAAGAACTCTGTAGCACCGCCTACATACCTCGCTCTGCTAATCCTGTTACCAGTGG<br>CTGCTGCCAGTGGCGATAAGTCGTGTCTTACCGGGTTGGACTCAAGACGATAGTTACCGGATAAGGC<br>GCAGCGGTCGGGCTGAACGGGGGGTTCGTGCACACAGCCCAGCTTGGAGCGAACGACCTACACCGA<br>ACTGAGATACCTACAGCGTGAGCTATGAGAAAGCGCCACGCTTCCCGAAGGGAGAAAGGCGGACAG<br>GTATCCGGTAAGCGGCAGGGTCGGAACAGGAGAGCGCACGAGGGAGCTTCCAGGGGGAAACGCCT<br>GGTATCTTTATAGTCCTGTCGGGTTTCGCCACCTCTGACTTGAGCGTCGATTTTTGTGATGCTCGTCAG<br>GGGGGCGGAGCCTATGGAAAAACGCCAGCAACGCGGCCTTTTTACGGTTCCTGGCCTTTTGCTGGCCT<br>TTTGCTCACATGTTCTTTCCTGCGTTATCCCCTGATTCTGTGGATAACCGTATTACCGCCTTTGAGTGAG<br>CTGATACCGCTCGCCGCAGCCGAACGACCGAGCGCAGCGAGTCAGTGAGCGAGGAAGCGGAAGAGC<br>GCCTGATGCGGTATTTTCTCCTTACGCATCTGTGCGGTATTTCACACCGCATATGGTGCACTCTCAGTA<br>CAATCTGCTCTGATGCCGCATAGTTAAGCCAGTATACACTCCGCTATCGCTACGTGACTGGGTCATGG<br>CTGCGCCCCGACACCCGCCAACACCCGCTGACGCGCCCTGACGGGCTTGTCTGCTCCCGGCATCCGCT<br>TACAGACAAGCTGTGACCGTCTCCGGGAGCTGCATGTGTCAGAGGTTTTCACCGTCATCACCGAAACG<br>CGCGAGGCAGCAGATCAATTCGCGCGCGAAGGCGAAGCGGCATGCATAATGTGCCTGTCAAATGGA<br>CGAAGCAGGGATTCTGCAAACCCTATGCTACTCCGTCAAGCCGTCAATTGTCTGATTCGTTACCAATTA<br>TGACAACTTGACGGCTACATCATTCACTTTTTCTTCACAACCGGCACGGAACTCGCTCGGGCTGGCCCC<br>GGTGCATTTTTTAAATACCCGCGAGAAATAGAGTTGATCGTCAAAACCAACATTGCGACCGACGGTGG<br>CGATAGGCATCCGGGTGGTGCTCAAAAGCAGCTTCGCCTGGCTGATACGTTGGTCCTCGCGCCAGCTT<br>AAGACGCTAATCCCTAACTGCTGGCGGAAAAGATGTGACAGACGCGACGGCGACAAGCAAACATGCT<br>GTGCGACGCTGGCGATATCAAAATTGCTGTCTGCCAGGTGATCGCTGATGTACTGACAAGCCTCGCGT<br>ACCCGATTATCCATCGGTGGATGGAGCGACTCGTTAATCGCTTCCATGCGCCGCAGTAACAATTGCTC<br>AAGCAGATTTATCGCCAGCAGCTCCGAATAGCGCCCTTCCCCTTGCCCGGCGTTAATGATTTGCCCAAA<br>CAGGTCGCTGAAATGCGGCTGGTGCGCTTCATCCGGGCGAAAGAACCCCGTATTGGCAAATATTGAC<br>GGCCAGTTAAGCCATTCATGCCAGTAGGCGCGCGGACGAAAGTAAACCCACTGGTGATACCATTCGC<br>GAGCCTCCGGATGACGACCGTAGTGATGAATCTCTCCTGGCGGGAACAGCAAAATATCACCCGGTCG<br>GCAAACAAATTCTCGTCCCTGATTTTTCACCACCCCCTGACCGCGAATGGTGAGATTGAGAATATAACC<br>TTTCATTCCCAGCGGTCGGTCGATAAAAAAATCGAGATAACCGTTGGCCTCAATCGGCGTTAAACCCG<br>CCACCAGATGGGCATTAAACGAGTATCCCGGCAGCAGGGGATCATTTTGCGCTTCAGCCATACTTTTC<br>ATACTCCCGCCATTCAGAG
pBAD:Δss-MalE-TCD-mScarlet-I3	AAGAAACCAATTGTCCATATTGCATCAGACATTGCCGTCACTGCGTCTTTTACTGGCTCTTCTCGCTAAC<br>CAAACCGGTAACCCCGCTTATTAAAAGCATTCTGTAACAAAGCGGGACCAAAGCCATGACAAAAACG<br>CGTAACAAAAGTGTCTATAATCACGGCAGAAAAGTCCACATTGATTATTTGCACGGCGTCACACTTTG<br>CTATGCCATAGCATTTTTATCCATAAGATTAGCGGATCCTACCTGACGCTTTTTATCGCAACTCTCTACT<br>GTTTCTCCATACCCGTTTTTTTGGGCTAGAAATAATTTTGTTTAACTTTAAGAAGGAGATATACATATGA<br>AAATCGAAGAAGGTAAACTGGTAATCTGGATTAACGGCGATAAAGGCTATAACGGTCTCGCTGAAGT<br>CGGTAAGAAATTCGAGAAAGATACCGGAATTAAAGTCACCGTTGAGCATCCGGATAAACTGGAAGAG<br>AAATTCCCACAGGTTGCGGCAACTGGCGATGGCCCTGACATTATCTTCTGGGCACACGACCGCTTTGG<br>TGGCTACGCTCAATCTGGCCTGTTGGCTGAAATCACCCCGGACAAAGCGTTCCAGGACAAGCTGTATC<br>CGTTTACCTGGGATGCCGTACGTTACAACGGCAAGCTGATTGCTTACCCGATCGCTGTTGAAGCGTTA<br>TCGCTGATTTATAACAAAGATCTGCTGCCGAACCCGCCAAAAACCTGGGAAGAGATCCCGGCGCTGG<br>ATAAAGAACTGAAAGCGAAAGGTAAGAGCGCGCTGATGTTCAACCTGCAAGAACCGTACTTCACCTG<br>GCCGCTGATTGCTGCTGACGGGGGTTATGCGTTCAAGTATGAAAACGGCAAGTACGACATTAAAGAC<br>GTGGGCGTGGATAACGCTGGCGCGAAAGCGGGTCTGACCTTCCTGGTTGACCTGATTAAAAACAAAC<br>ACATGAATGCAGACACCGATTACTCCATCGCAGAAGCTGCCTTTAATAAAGGCGAAACAGCGATGACC<br>ATCAACGGCCCGTGGGCATGGTCCAACATCGACACCAGCAAAGTGAATTATGGTGTAACGGTACTGC<br>CGACCTTCAAGGGTCAACCATCCAAACCGTTCGTTGGCGTGCTGAGCGCAGGTATTAACGCCGCCAGT

	CCGAACAAAGAGCTGGCGAAAGAGTTCCTCGAAAACTATCTGCTGACTGATGAAGGTCTGGAAGCGG<br>TTAATAAAGACAAACCGCTGGGTGCCGTAGCGCTGAAGTCTTACGAGGAAGAGTTGGCGAAAGATCC<br>ACGTATTGCCGCCACCATGGAAAACGCCCAGAAAGGTGAAATCATGCCGAACATCCCGCAGATGTCC<br>GCTTTCTGGTATGCCGTGCGTACTGCGGTGATCAACGCCGCCAGCGGTCGTCAGACTGTCGATGAAGC<br>CCTGAAAGACGCGCAGACTACTAGTCATCATCACCACCATCATTAGGATGGTGGTGATGATAATGGAT<br>AGCACCGAGGCAGTGATCAAGGAGTTCATGCGGTTCAAGGTGCACATGGAGGGCTCCATGAACGGC<br>CACGAGTTCGAGATCGAGGGCGAGGGCGAGGGCCGCCCCTACGAGGGCACCCAGACCGCCAAGCTG<br>AAGGTGACCAAGGGTGGCCCCCTGCCCTTCTCCTGGGACATCCTGTCCCCTCAGTTCATGTACGGCTCC<br>AGGGCCTTCATCAAGCACCCCGCCGACATCCCCGACTACTGGAAGCAGTCCTTCCCCGAGGGCTTCAA<br>GTGGGAGCGCGTGATGATCTTCGAGGACGGCGGCACCGTGTCCGTGACCCAGGACACCTCCCTGGAG<br>GACGGCACCCTGATCTACAAGGTGAAGCTCCGCGGCGGCAACTTCCCTCCTGACGGCCCCGTAATGCA<br>GAAGAGGACAATGGGCTGGGAAGCATCCACCGAGCGGTTGTACCCCGAGGACGTCGTGCTGAAGGG<br>CGACATTAAGATGGCCCTGCGCCTGAAGGACGGCGGCCGCTACCTGGCGGACTTCAAGACCACCTAC<br>AAGGCCAAGAAGCCCGTGCAGATGCCCGGCGCCTTCAACATCGACCGCAAGTTGGACATCACCTCCC<br>ACAACGAGGACTACACCGTGGTGGAACAGTACGAACGCTCCGTGGCCCGCCACTCCACCGGCGGCTC<br>CGGTGGCTCCTAAAAGCTTACGGTCTCCAGCTTGGCTGTTTTGGCGGATGAGAGAAGATTTTCAGCCT<br>GATACAGATTAAATCAGAACGCAGAAGCGGTCTGATAAAACAGAATTTGCCTGGCGGCAGTAGCGCG<br>GTGGTCCCACCTGACCCCATGCCGAACTCAGAAGTGAAACGCCGTAGCGCCGATGGTAGTGTGGGGT<br>CTCCCCATGCGAGAGTAGGGAACTGCCAGGCATCAAATAAAACGAAAGGCTCAGTCGAAAGACTGG<br>GCCTTTCGTTTTATCTGTTGTTTGTCGGTGAACGCTCTCCTGAGTAGGACAAATCCGCCGGGAGCGGA<br>TTTGAACGTTGCGAAGCAACGGCCCGGAGGGTGGCGGGCAGGACGCCCGCCATAAACTGCCAGGCA<br>TCAAATTAAGCAGAAGGCCATCCTGACGGATGGCCTTTTTGCGTTTCTACAAACTCTCATGAACAATAA<br>AACTGTCTGCTTACATAAACAGTAATACAAGGGGTGTTATGAGCCATATTCAACGGGAAACGTCTTGC<br>TCTAGGCCGCGATTAAATTCCAACATGGATGCTGATTTATATGGGTATAAATGGGCTCGCGATAATGT<br>CGGGCAATCAGGTGCGACAATCTATCGATTGTATGGGAAGCCCGATGCGCCAGAGTTGTTTCTGAAA<br>CATGGCAAAGGTAGCGTTGCCAATGATGTTACAGATGAGATGGTCAGACTAAACTGGCTGACGGAAT<br>TTATGCCTCTTCCGACCATCAAGCATTTTATCCGTACTCCTGATGATGCATGGTTACTCACCACTGCGAT<br>CCCCGGGAAAACAGCATTCCAGGTATTAGAAGAATATCCTGATTCAGGTGAAAATATTGTTGATGCGC<br>TGGCAGTGTTCCTGCGCCGGTTGCATTCGATTCCTGTTTGTAATTGTCCTTTTAACAGCGATCGCGTAT<br>TTCGTCTCGCTCAGGCGCAATCACGAATGAATAACGGTTTGGTTGATGCGAGTGATTTTGATGACGAG<br>CGTAATGGCTGGCCTGTTGAACAAGTCTGGAAAGAAATGCATAAACTTTTGCCATTCTCACCGGATTC<br>AGTCGTCACTCATGGTGATTTCTCACTTGATAACCTTATTTTTGACGAGGGGAAATTAATAGGTTGTAT<br>TGATGTTGGACGAGTCGGAATCGCAGACCGATACCAGGATCTTGCCATCCTATGGAACTGCCTCGGTG<br>AGTTTTCTCCTTCATTACAGAAACGGCTTTTTCAAAAATATGGTATTGATAATCCTGATATGAATAAATT<br>GCAGTTTCATTTGATGCTCGATGAGTTTTTCTAAGCTGTCAGACCAAGTTTACTCATATATACTTTAGAT<br>TGATTTAAAACTTCATTTTTAATTTAAAAGGATCTAGGTGAAGATCCTTTTTGATAATCTCATGACCAAA<br>ATCCCTTAACGTGAGTTTTCGTTCCACTGAGCGTCAGACCCCGTAGAAAAGATCAAAGGATCTTCTTGA<br>GATCCTTTTTTTCTGCGCGTAATCTGCTGCTTGCAAACAAAAAAACCACCGCTACCAGCGGTGGTTTGT<br>TTGCCGGATCAAGAGCTACCAACTCTTTTTCCGAAGGTAACTGGCTTCAGCAGAGCGCAGATACCAAA<br>TACTGTCCTTCTAGTGTAGCCGTAGTTAGGCCACCACTTCAAGAACTCTGTAGCACCGCCTACATACCT<br>CGCTCTGCTAATCCTGTTACCAGTGGCTGCTGCCAGTGGCGATAAGTCGTGTCTTACCGGGTTGGACT<br>CAAGACGATAGTTACCGGATAAGGCGCAGCGGTCGGGCTGAACGGGGGGTTCGTGCACACAGCCCA<br>GCTTGGAGCGAACGACCTACACCGAACTGAGATACCTACAGCGTGAGCTATGAGAAAGCGCCACGCT<br>TCCCGAAGGGAGAAAGGCGGACAGGTATCCGGTAAGCGGCAGGGTCGGAACAGGAGAGCGCACGA<br>GGGAGCTTCCAGGGGGAAACGCCTGGTATCTTTATAGTCCTGTCGGGTTTCGCCACCTCTGACTTGAG<br>CGTCGATTTTTGTGATGCTCGTCAGGGGGGCGGAGCCTATGGAAAAACGCCAGCAACGCGGCCTTTT<br>TACGGTTCCTGGCCTTTTGCTGGCCTTTTGCTCACATGTTCTTTCCTGCGTTATCCCCTGATTCTGTGGA<br>TAACCGTATTACCGCCTTTGAGTGAGCTGATACCGCTCGCCGCAGCCGAACGACCGAGCGCAGCGAG<br>TCAGTGAGCGAGGAAGCGGAAGAGCGCCTGATGCGGTATTTTCTCCTTACGCATCTGTGCGGTATTTC<br>ACACCGCATATGGTGCACTCTCAGTACAATCTGCTCTGATGCCGCATAGTTAAGCCAGTATACACTCCG<br>CTATCGCTACGTGACTGGGTCATGGCTGCGCCCCGACACCCGCCAACACCCGCTGACGCGCCCTGACG<br>GGCTTGTCTGCTCCCGGCATCCGCTTACAGACAAGCTGTGACCGTCTCCGGGAGCTGCATGTGTCAGA<br>GGTTTTCACCGTCATCACCGAAACGCGCGAGGCAGCAGATCAATTCGCGCGCGAAGGCGAAGCGGCA<br>TGCATAATGTGCCTGTCAAATGGACGAAGCAGGGATTCTGCAAACCCTATGCTACTCCGTCAAGCCGT<br>CAATTGTCTGATTCGTTACCAATTATGACAACTTGACGGCTACATCATTCACTTTTTCTTCACAACCGGC<br>ACGGAACTCGCTCGGGCTGGCCCCGGTGCATTTTTTAAATACCCGCGAGAAATAGAGTTGATCGTCAA<br>AACCAACATTGCGACCGACGGTGGCGATAGGCATCCGGGTGGTGCTCAAAAGCAGCTTCGCCTGGCT<br>GATACGTTGGTCCTCGCGCCAGCTTAAGACGCTAATCCCTAACTGCTGGCGGAAAAGATGTGACAGA<br>CGCGACGGCGACAAGCAAACATGCTGTGCGACGCTGGCGATATCAAAATTGCTGTCTGCCAGGTGAT<br>CGCTGATGTACTGACAAGCCTCGCGTACCCGATTATCCATCGGTGGATGGAGCGACTCGTTAATCGCT<br>TCCATGCGCCGCAGTAACAATTGCTCAAGCAGATTTATCGCCAGCAGCTCCGAATAGCGCCCTTCCCCT<br>TGCCCGGCGTTAATGATTTGCCCAAACAGGTCGCTGAAATGCGGCTGGTGCGCTTCATCCGGGCGAA<br>AGAACCCCGTATTGGCAAATATTGACGGCCAGTTAAGCCATTCATGCCAGTAGGCGCGCGGACGAAA

	GTAAACCCACTGGTGATACCATTCGCGAGCCTCCGGATGACGACCGTAGTGATGAATCTCTCCTGGCG<br>GGAACAGCAAAATATCACCCGGTCGGCAAACAAATTCTCGTCCCTGATTTTTCACCACCCCCTGACCGC<br>GAATGGTGAGATTGAGAATATAACCTTTCATTCCCAGCGGTCGGTCGATAAAAAAATCGAGATAACC<br>GTTGGCCTCAATCGGCGTTAAACCCGCCACCAGATGGGCATTAAACGAGTATCCCGGCAGCAGGGGA<br>TCATTTTGCGCTTCAGCCATACTTTTCATACTCCCGCCATTCAGAG
pBAD:pre^{MalE}-MalE-TCD-mScarlet-I3	AAGAAACCAATTGTCCATATTGCATCAGACATTGCCGTCACTGCGTCTTTTACTGGCTCTTCTCGCTAAC<br>CAAACCGGTAACCCCGCTTATTAAAAGCATTCTGTAACAAAGCGGGACCAAAGCCATGACAAAAACG<br>CGTAACAAAAGTGTCTATAATCACGGCAGAAAAGTCCACATTGATTATTTGCACGGCGTCACACTTTG<br>CTATGCCATAGCATTTTTATCCATAAGATTAGCGGATCCTACCTGACGCTTTTTATCGCAACTCTCTACT<br>GTTTCTCCATACCCGTTTTTTTGGGCTAGAAATAATTTTGTTTAACTTTAAGAAGGAGATATACATATGA<br>AAATAAAAACAGGTGCACGCATCCTCGCATTATCCGCATTAACGACGATGATGTTTTCCGCCTCGGCTC<br>TCGCCAAAATCGAAGAAGGTAAACTGGTAATCTGGATTAACGGCGATAAAGGCTATAACGGTCTCGC<br>TGAAGTCGGTAAGAAATTCGAGAAAGATACCGGAATTAAAGTCACCGTTGAGCATCCGGATAAACTG<br>GAAGAGAAATTCCCACAGGTTGCGGCAACTGGCGATGGCCCTGACATTATCTTCTGGGCACACGACC<br>GCTTTGGTGGCTACGCTCAATCTGGCCTGTTGGCTGAAATCACCCCGGACAAAGCGTTCCAGGACAAG<br>CTGTATCCGTTTACCTGGGATGCCGTACGTTACAACGGCAAGCTGATTGCTTACCCGATCGCTGTTGAA<br>GCGTTATCGCTGATTTATAACAAAGATCTGCTGCCGAACCCGCCAAAAACCTGGGAAGAGATCCCGGC<br>GCTGGATAAAGAACTGAAAGCGAAAGGTAAGAGCGCGCTGATGTTCAACCTGCAAGAACCGTACTTC<br>ACCTGGCCGCTGATTGCTGCTGACGGGGGTTATGCGTTCAAGTATGAAAACGGCAAGTACGACATTA<br>AAGACGTGGGCGTGGATAACGCTGGCGCGAAAGCGGGTCTGACCTTCCTGGTTGACCTGATTAAAAA<br>CAAACACATGAATGCAGACACCGATTACTCCATCGCAGAAGCTGCCTTTAATAAAGGCGAAACAGCG<br>ATGACCATCAACGGCCCGTGGGCATGGTCCAACATCGACACCAGCAAAGTGAATTATGGTGTAACGG<br>TACTGCCGACCTTCAAGGGTCAACCATCCAAACCGTTCGTTGGCGTGCTGAGCGCAGGTATTAACGCC<br>GCCAGTCCGAACAAAGAGCTGGCGAAAGAGTTCCTCGAAAACTATCTGCTGACTGATGAAGGTCTGG<br>AAGCGGTTAATAAAGACAAACCGCTGGGTGCCGTAGCGCTGAAGTCTTACGAGGAAGAGTTGGCGA<br>AAGATCCACGTATTGCCGCCACCATGGAAAACGCCCAGAAAGGTGAAATCATGCCGAACATCCCGCA<br>GATGTCCGCTTTCTGGTATGCCGTGCGTACTGCGGTGATCAACGCCGCCAGCGGTCGTCAGACTGTCG<br>ATGAAGCCCTGAAAGACGCGCAGACTACTAGTCATCATCACCACCATCATTAGGATGGTGGTGATGAT<br>AATGGATAGCACCGAGGCAGTGATCAAGGAGTTCATGCGGTTCAAGGTGCACATGGAGGGCTCCAT<br>GAACGGCCACGAGTTCGAGATCGAGGGCGAGGGCGAGGGCCGCCCCTACGAGGGCACCCAGACCG<br>CCAAGCTGAAGGTGACCAAGGGTGGCCCCCTGCCCTTCTCCTGGGACATCCTGTCCCCTCAGTTCATG<br>TACGGCTCCAGGGCCTTCATCAAGCACCCCGCCGACATCCCCGACTACTGGAAGCAGTCCTTCCCCGA<br>GGGCTTCAAGTGGGAGCGCGTGATGATCTTCGAGGACGGCGGCACCGTGTCCGTGACCCAGGACAC<br>CTCCCTGGAGGACGGCACCCTGATCTACAAGGTGAAGCTCCGCGGCGGCAACTTCCCTCCTGACGGCC<br>CCGTAATGCAGAAGAGGACAATGGGCTGGGAAGCATCCACCGAGCGGTTGTACCCCGAGGACGTCG<br>TGCTGAAGGGCGACATTAAGATGGCCCTGCGCCTGAAGGACGGCGGCCGCTACCTGGCGGACTTCAA<br>GACCACCTACAAGGCCAAGAAGCCCGTGCAGATGCCCGGCGCCTTCAACATCGACCGCAAGTTGGAC<br>ATCACCTCCCACAACGAGGACTACACCGTGGTGGAACAGTACGAACGCTCCGTGGCCCGCCACTCCAC<br>CGGCGGCTCCGGTGGCTCCTAAAAGCTTACGGTCTCCAGCTTGGCTGTTTTGGCGGATGAGAGAAGA<br>TTTTCAGCCTGATACAGATTAAATCAGAACGCAGAAGCGGTCTGATAAAACAGAATTTGCCTGGCGGC<br>AGTAGCGCGGTGGTCCCACCTGACCCCATGCCGAACTCAGAAGTGAAACGCCGTAGCGCCGATGGTA<br>GTGTGGGGTCTCCCCATGCGAGAGTAGGGAACTGCCAGGCATCAAATAAAACGAAAGGCTCAGTCG<br>AAAGACTGGGCCTTTCGTTTTATCTGTTGTTTGTCGGTGAACGCTCTCCTGAGTAGGACAAATCCGCCG<br>GGAGCGGATTTGAACGTTGCGAAGCAACGGCCCGGAGGGTGGCGGGCAGGACGCCCGCCATAAACT<br>GCCAGGCATCAAATTAAGCAGAAGGCCATCCTGACGGATGGCCTTTTTGCGTTTCTACAAACTCTCAT<br>GAACAATAAAACTGTCTGCTTACATAAACAGTAATACAAGGGGTGTTATGAGCCATATTCAACGGGAA<br>ACGTCTTGCTCTAGGCCGCGATTAAATTCCAACATGGATGCTGATTTATATGGGTATAAATGGGCTCG<br>CGATAATGTCGGGCAATCAGGTGCGACAATCTATCGATTGTATGGGAAGCCCGATGCGCCAGAGTTG<br>TTTCTGAAACATGGCAAAGGTAGCGTTGCCAATGATGTTACAGATGAGATGGTCAGACTAAACTGGCT<br>GACGGAATTTATGCCTCTTCCGACCATCAAGCATTTTATCCGTACTCCTGATGATGCATGGTTACTCAC<br>CACTGCGATCCCCGGGAAAACAGCATTCCAGGTATTAGAAGAATATCCTGATTCAGGTGAAAATATTG<br>TTGATGCGCTGGCAGTGTTCCTGCGCCGGTTGCATTCGATTCCTGTTTGTAATTGTCCTTTTAACAGCG<br>ATCGCGTATTTCGTCTCGCTCAGGCGCAATCACGAATGAATAACGGTTTGGTTGATGCGAGTGATTTT<br>GATGACGAGCGTAATGGCTGGCCTGTTGAACAAGTCTGGAAAGAAATGCATAAACTTTTGCCATTCTC<br>ACCGGATTCAGTCGTCACTCATGGTGATTTCTCACTTGATAACCTTATTTTTGACGAGGGGAAATTAAT<br>AGGTTGTATTGATGTTGGACGAGTCGGAATCGCAGACCGATACCAGGATCTTGCCATCCTATGGAACT<br>GCCTCGGTGAGTTTTCTCCTTCATTACAGAAACGGCTTTTTCAAAAATATGGTATTGATAATCCTGATAT<br>GAATAAATTGCAGTTTCATTTGATGCTCGATGAGTTTTTCTAAGCTGTCAGACCAAGTTTACTCATATAT<br>ACTTTAGATTGATTTAAAACTTCATTTTTAATTTAAAAGGATCTAGGTGAAGATCCTTTTTGATAATCTC<br>ATGACCAAAATCCCTTAACGTGAGTTTTCGTTCCACTGAGCGTCAGACCCCGTAGAAAAGATCAAAGG<br>ATCTTCTTGAGATCCTTTTTTTCTGCGCGTAATCTGCTGCTTGCAAACAAAAAAACCACCGCTACCAGC<br>GGTGGTTTGTTTGCCGGATCAAGAGCTACCAACTCTTTTTCCGAAGGTAACTGGCTTCAGCAGAGCGC

	AGATACCAAATACTGTCCTTCTAGTGTAGCCGTAGTTAGGCCACCACTTCAAGAACTCTGTAGCACCGC<br>CTACATACCTCGCTCTGCTAATCCTGTTACCAGTGGCTGCTGCCAGTGGCGATAAGTCGTGTCTTACCG<br>GGTTGGACTCAAGACGATAGTTACCGGATAAGGCGCAGCGGTCGGGCTGAACGGGGGGTTCGTGCA<br>CACAGCCCAGCTTGGAGCGAACGACCTACACCGAACTGAGATACCTACAGCGTGAGCTATGAGAAAG<br>CGCCACGCTTCCCGAAGGGAGAAAGGCGGACAGGTATCCGGTAAGCGGCAGGGTCGGAACAGGAG<br>AGCGCACGAGGGAGCTTCCAGGGGGAAACGCCTGGTATCTTTATAGTCCTGTCGGGTTTCGCCACCTC<br>TGACTTGAGCGTCGATTTTTGTGATGCTCGTCAGGGGGGCGGAGCCTATGGAAAAACGCCAGCAACG<br>CGGCCTTTTTACGGTTCCTGGCCTTTTGCTGGCCTTTTGCTCACATGTTCTTTCCTGCGTTATCCCCTGAT<br>TCTGTGGATAACCGTATTACCGCCTTTGAGTGAGCTGATACCGCTCGCCGCAGCCGAACGACCGAGCG<br>CAGCGAGTCAGTGAGCGAGGAAGCGGAAGAGCGCCTGATGCGGTATTTTCTCCTTACGCATCTGTGC<br>GGTATTTCACACCGCATATGGTGCACTCTCAGTACAATCTGCTCTGATGCCGCATAGTTAAGCCAGTAT<br>ACACTCCGCTATCGCTACGTGACTGGGTCATGGCTGCGCCCCGACACCCGCCAACACCCGCTGACGCG<br>CCCTGACGGGCTTGTCTGCTCCCGGCATCCGCTTACAGACAAGCTGTGACCGTCTCCGGGAGCTGCAT<br>GTGTCAGAGGTTTTCACCGTCATCACCGAAACGCGCGAGGCAGCAGATCAATTCGCGCGCGAAGGCG<br>AAGCGGCATGCATAATGTGCCTGTCAAATGGACGAAGCAGGGATTCTGCAAACCCTATGCTACTCCGT<br>CAAGCCGTCAATTGTCTGATTCGTTACCAATTATGACAACTTGACGGCTACATCATTCACTTTTTCTTCA<br>CAACCGGCACGGAACTCGCTCGGGCTGGCCCCGGTGCATTTTTTAAATACCCGCGAGAAATAGAGTT<br>GATCGTCAAAACCAACATTGCGACCGACGGTGGCGATAGGCATCCGGGTGGTGCTCAAAAGCAGCTT<br>CGCCTGGCTGATACGTTGGTCCTCGCGCCAGCTTAAGACGCTAATCCCTAACTGCTGGCGGAAAAGAT<br>GTGACAGACGCGACGGCGACAAGCAAACATGCTGTGCGACGCTGGCGATATCAAAATTGCTGTCTGC<br>CAGGTGATCGCTGATGTACTGACAAGCCTCGCGTACCCGATTATCCATCGGTGGATGGAGCGACTCGT<br>TAATCGCTTCCATGCGCCGCAGTAACAATTGCTCAAGCAGATTTATCGCCAGCAGCTCCGAATAGCGC<br>CCTTCCCCTTGCCCGGCGTTAATGATTTGCCCAAACAGGTCGCTGAAATGCGGCTGGTGCGCTTCATCC<br>GGGCGAAAGAACCCCGTATTGGCAAATATTGACGGCCAGTTAAGCCATTCATGCCAGTAGGCGCGCG<br>GACGAAAGTAAACCCACTGGTGATACCATTCGCGAGCCTCCGGATGACGACCGTAGTGATGAATCTCT<br>CCTGGCGGGAACAGCAAAATATCACCCGGTCGGCAAACAAATTCTCGTCCCTGATTTTTCACCACCCCC<br>TGACCGCGAATGGTGAGATTGAGAATATAACCTTTCATTCCCAGCGGTCGGTCGATAAAAAAATCGA<br>GATAACCGTTGGCCTCAATCGGCGTTAAACCCGCCACCAGATGGGCATTAAACGAGTATCCCGGCAG<br>CAGGGGATCATTTTGCGCTTCAGCCATACTTTTCATACTCCCGCCATTCAGAG
pBAD:pre^{MalE}-scFv-TCD-mScarlet-I3	AAGAAACCAATTGTCCATATTGCATCAGACATTGCCGTCACTGCGTCTTTTACTGGCTCTTCTCGCTAAC<br>CAAACCGGTAACCCCGCTTATTAAAAGCATTCTGTAACAAAGCGGGACCAAAGCCATGACAAAAACG<br>CGTAACAAAAGTGTCTATAATCACGGCAGAAAAGTCCACATTGATTATTTGCACGGCGTCACACTTTG<br>CTATGCCATAGCATTTTTATCCATAAGATTAGCGGATCCTACCTGACGCTTTTTATCGCAACTCTCTACT<br>GTTTCTCCATACCCGTTTTTTTGGGCTAGAAATAATTTTGTTTAACTTTAAGAAGGAGATATACATATGA<br>AAATAAAAACAGGTGCACGCATCCTCGCATTATCCGCATTAACGACGATGATGTTTTCCGCCTCGGCTC<br>TCGCCGAAGTGCAGCTGGTCGAATCGGGTGGCGGATTAGTGCAGCCTGGAGGCTCCTTACGCCTGAG<br>CTGTGCAGCGAGCGGCTTCAACATCAAGGACACCTACATACATTGGGTTCGCCAAGCTCCGGGCAAA<br>GGTCTGGAGTGGGTTGCTCGTATCTATCCCACTAATGGGTATACACGCTATGCCGATAGCGTGAAAGG<br>CCGGTTTACCATTAGCGCCGATACGAGCAAGAATACGGCGTATCTGCAGATGAACTCTCTGCGTGCCG<br>AAGATACAGCGGTCTACTACTGCTCTCGTTGGGGTGGTGACGGGTTTTATGCAATGGACTATTGGGG<br>CCAAGGAACCCTCGTGACGGTTTCCTCAGGCGGAGGTGGTAGTGGTGGCGGTGGGTCTGGCGGCGG<br>TGGGAGCGACATTCAGATGACGCAGTCACCATCGTCGTTGTCAGCGTCGGTAGGTGATCGCGTCACG<br>ATTACCTGTCGTGCATCCCAAGATGTGAACACTGCAGTAGCGTGGTACCAGCAGAAACCGGGGAAAG<br>CTCCGAAACTTCTGATTTACTCGGCGAGTTTCCTGTATAGTGGCGTTCCAAGTCGCTTTAGCGGTTCCC<br>GTTCTGGCACGGATTTCACACTGACCATCTCAAGCTTGCAGCCGGAAGATTTTGCCACCTATTACTGCC<br>AACAGCACTATACCACTCCTCCGACCTTTGGCCAAGGCACCAAAGTGGAGATCAAACGCACTAGTCAT<br>CATCACCACCATCATTAGGATGGTGGTGATGATAATGGATAGCACCGAGGCAGTGATCAAGGAGTTC<br>ATGCGGTTCAAGGTGCACATGGAGGGCTCCATGAACGGCCACGAGTTCGAGATCGAGGGCGAGGGC<br>GAGGGCCGCCCCTACGAGGGCACCCAGACCGCCAAGCTGAAGGTGACCAAGGGTGGCCCCCTGCCC<br>TTCTCCTGGGACATCCTGTCCCCTCAGTTCATGTACGGCTCCAGGGCCTTCATCAAGCACCCCGCCGAC<br>ATCCCCGACTACTGGAAGCAGTCCTTCCCCGAGGGCTTCAAGTGGGAGCGCGTGATGATCTTCGAGG<br>ACGGCGGCACCGTGTCCGTGACCCAGGACACCTCCCTGGAGGACGGCACCCTGATCTACAAGGTGAA<br>GCTCCGCGGCGGCAACTTCCCTCCTGACGGCCCCGTAATGCAGAAGAGGACAATGGGCTGGGAAGCA<br>TCCACCGAGCGGTTGTACCCCGAGGACGTCGTGCTGAAGGGCGACATTAAGATGGCCCTGCGCCTGA<br>AGGACGGCGGCCGCTACCTGGCGGACTTCAAGACCACCTACAAGGCCAAGAAGCCCGTGCAGATGCC<br>CGGCGCCTTCAACATCGACCGCAAGTTGGACATCACCTCCCACAACGAGGACTACACCGTGGTGGAA<br>CAGTACGAACGCTCCGTGGCCCGCCACTCCACCGGCGGCTCCGGTGGCTCCTAAAAGCTTACGGTCTC<br>CAGCTTGGCTGTTTTGGCGGATGAGAGAAGATTTTCAGCCTGATACAGATTAAATCAGAACGCAGAA<br>GCGGTCTGATAAAACAGAATTTGCCTGGCGGCAGTAGCGCGGTGGTCCCACCTGACCCCATGCCGAA<br>CTCAGAAGTGAAACGCCGTAGCGCCGATGGTAGTGTGGGGTCTCCCCATGCGAGAGTAGGGAACTG<br>CCAGGCATCAAATAAAACGAAAGGCTCAGTCGAAAGACTGGGCCTTTCGTTTTATCTGTTGTTTGTCG<br>GTGAACGCTCTCCTGAGTAGGACAAATCCGCCGGGAGCGGATTTGAACGTTGCGAAGCAACGGCCCG

	GAGGGTGGCGGGCAGGACGCCCGCCATAAACTGCCAGGCATCAAATTAAGCAGAAGGCCATCCTGA<br>CGGATGGCCTTTTTGCGTTTCTACAAACTCTCATGAACAATAAAACTGTCTGCTTACATAAACAGTAAT<br>ACAAGGGGTGTTATGAGCCATATTCAACGGGAAACGTCTTGCTCTAGGCCGCGATTAAATTCCAACAT<br>GGATGCTGATTTATATGGGTATAAATGGGCTCGCGATAATGTCGGGCAATCAGGTGCGACAATCTATC<br>GATTGTATGGGAAGCCCGATGCGCCAGAGTTGTTTCTGAAACATGGCAAAGGTAGCGTTGCCAATGA<br>TGTTACAGATGAGATGGTCAGACTAAACTGGCTGACGGAATTTATGCCTCTTCCGACCATCAAGCATT<br>TTATCCGTACTCCTGATGATGCATGGTTACTCACCACTGCGATCCCCGGGAAAACAGCATTCCAGGTAT<br>TAGAAGAATATCCTGATTCAGGTGAAAATATTGTTGATGCGCTGGCAGTGTTCCTGCGCCGGTTGCAT<br>TCGATTCCTGTTTGTAATTGTCCTTTTAACAGCGATCGCGTATTTCGTCTCGCTCAGGCGCAATCACGA<br>ATGAATAACGGTTTGGTTGATGCGAGTGATTTTGATGACGAGCGTAATGGCTGGCCTGTTGAACAAG<br>TCTGGAAAGAAATGCATAAACTTTTGCCATTCTCACCGGATTCAGTCGTCACTCATGGTGATTTCTCAC<br>TTGATAACCTTATTTTTGACGAGGGGAAATTAATAGGTTGTATTGATGTTGGACGAGTCGGAATCGCA<br>GACCGATACCAGGATCTTGCCATCCTATGGAACTGCCTCGGTGAGTTTTCTCCTTCATTACAGAAACGG<br>CTTTTTCAAAAATATGGTATTGATAATCCTGATATGAATAAATTGCAGTTTCATTTGATGCTCGATGAG<br>TTTTTCTAAGCTGTCAGACCAAGTTTACTCATATATACTTTAGATTGATTTAAAACTTCATTTTTAATTTA<br>AAAGGATCTAGGTGAAGATCCTTTTTGATAATCTCATGACCAAAATCCCTTAACGTGAGTTTTCGTTCC<br>ACTGAGCGTCAGACCCCGTAGAAAAGATCAAAGGATCTTCTTGAGATCCTTTTTTTCTGCGCGTAATCT<br>GCTGCTTGCAAACAAAAAAACCACCGCTACCAGCGGTGGTTTGTTTGCCGGATCAAGAGCTACCAACT<br>CTTTTTCCGAAGGTAACTGGCTTCAGCAGAGCGCAGATACCAAATACTGTCCTTCTAGTGTAGCCGTA<br>GTTAGGCCACCACTTCAAGAACTCTGTAGCACCGCCTACATACCTCGCTCTGCTAATCCTGTTACCAGT<br>GGCTGCTGCCAGTGGCGATAAGTCGTGTCTTACCGGGTTGGACTCAAGACGATAGTTACCGGATAAG<br>GCGCAGCGGTCGGGCTGAACGGGGGGTTCGTGCACACAGCCCAGCTTGGAGCGAACGACCTACACC<br>GAACTGAGATACCTACAGCGTGAGCTATGAGAAAGCGCCACGCTTCCCGAAGGGAGAAAGGCGGAC<br>AGGTATCCGGTAAGCGGCAGGGTCGGAACAGGAGAGCGCACGAGGGAGCTTCCAGGGGGAAACGC<br>CTGGTATCTTTATAGTCCTGTCGGGTTTCGCCACCTCTGACTTGAGCGTCGATTTTTGTGATGCTCGTCA<br>GGGGGGCGGAGCCTATGGAAAAACGCCAGCAACGCGGCCTTTTTACGGTTCCTGGCCTTTTGCTGGC<br>CTTTTGCTCACATGTTCTTTCCTGCGTTATCCCCTGATTCTGTGGATAACCGTATTACCGCCTTTGAGTG<br>AGCTGATACCGCTCGCCGCAGCCGAACGACCGAGCGCAGCGAGTCAGTGAGCGAGGAAGCGGAAGA<br>GCGCCTGATGCGGTATTTTCTCCTTACGCATCTGTGCGGTATTTCACACCGCATATGGTGCACTCTCAG<br>TACAATCTGCTCTGATGCCGCATAGTTAAGCCAGTATACACTCCGCTATCGCTACGTGACTGGGTCATG<br>GCTGCGCCCCGACACCCGCCAACACCCGCTGACGCGCCCTGACGGGCTTGTCTGCTCCCGGCATCCGC<br>TTACAGACAAGCTGTGACCGTCTCCGGGAGCTGCATGTGTCAGAGGTTTTCACCGTCATCACCGAAAC<br>GCGCGAGGCAGCAGATCAATTCGCGCGCGAAGGCGAAGCGGCATGCATAATGTGCCTGTCAAATGG<br>ACGAAGCAGGGATTCTGCAAACCCTATGCTACTCCGTCAAGCCGTCAATTGTCTGATTCGTTACCAATT<br>ATGACAACTTGACGGCTACATCATTCACTTTTTCTTCACAACCGGCACGGAACTCGCTCGGGCTGGCCC<br>CGGTGCATTTTTTAAATACCCGCGAGAAATAGAGTTGATCGTCAAAACCAACATTGCGACCGACGGTG<br>GCGATAGGCATCCGGGTGGTGCTCAAAAGCAGCTTCGCCTGGCTGATACGTTGGTCCTCGCGCCAGC<br>TTAAGACGCTAATCCCTAACTGCTGGCGGAAAAGATGTGACAGACGCGACGGCGACAAGCAAACATG<br>CTGTGCGACGCTGGCGATATCAAAATTGCTGTCTGCCAGGTGATCGCTGATGTACTGACAAGCCTCGC<br>GTACCCGATTATCCATCGGTGGATGGAGCGACTCGTTAATCGCTTCCATGCGCCGCAGTAACAATTGC<br>TCAAGCAGATTTATCGCCAGCAGCTCCGAATAGCGCCCTTCCCCTTGCCCGGCGTTAATGATTTGCCCA<br>AACAGGTCGCTGAAATGCGGCTGGTGCGCTTCATCCGGGCGAAAGAACCCCGTATTGGCAAATATTG<br>ACGGCCAGTTAAGCCATTCATGCCAGTAGGCGCGCGGACGAAAGTAAACCCACTGGTGATACCATTC<br>GCGAGCCTCCGGATGACGACCGTAGTGATGAATCTCTCCTGGCGGGAACAGCAAAATATCACCCGGT<br>CGGCAAACAAATTCTCGTCCCTGATTTTTCACCACCCCCTGACCGCGAATGGTGAGATTGAGAATATA<br>ACCTTTCATTCCCAGCGGTCGGTCGATAAAAAAATCGAGATAACCGTTGGCCTCAATCGGCGTTAAAC<br>CCGCCACCAGATGGGCATTAAACGAGTATCCCGGCAGCAGGGGATCATTTTGCGCTTCAGCCATACTT<br>TTCATACTCCCGCCATTCAGAG

Table S5. Details on the construction of the expression plasmids.

Plasmid	Construction notes	Primers used (5'-3')
pBAD:Mth1-TCD-mCherry	This plasmid was constructed in the following steps; (1) The pBAD:pre^{PelB}-hGH-TCD-mCherry expression plasmid [5] was amplified by PCR using the ST8 / ST9 primers. (2) The coding sequence for Mth1 was amplified by PCR from pET28:Mth1 [6] using the MM83 / MM84 primers. (3) PCR products were *DpnI* treated and gel-purified, then ligated using the *in vivo* DNA assembly method in the *E. coli* MC1061 strain [4].	ST8: ATGTATATCTCCTTCTTAAAGTTAAAC<br>ST9: ACTAGTCATCATCACCACCATCATTAG<br>MM83: GCTAGAAATAATTTTGTTTAACTTTAAGAAGGAGATATACATATGGGTGCAAGCCGTCTGTATAC<br>MM84: CCATCCTAATGATGGTGGTGATGATGACTAGTCACGGTATCAACTTCACGCAGG
pBAD:pre^{MalE}-MalE-TCD-mCherry	This plasmid was constructed in the following steps; (1) The pBAD:pre^{MalE}-hGH-TCD-mCherry expression plasmid (see below) was amplified by PCR using the ST8 / ST9 primers. (2) The coding sequence for pre^{MalE}-MalE was amplified by PCR from pBAD:pre^{MalE}-MalE [5] using the MM88 / MM89 primers. (3) PCR products were *DpnI* treated and gel-purified, then ligated using the *in vivo* DNA assembly method in the *E. coli* MC1061 strain [4].	ST8: ATGTATATCTCCTTCTTAAAGTTAAAC<br>ST9: ACTAGTCATCATCACCACCATCATTAG<br>MM88: CCATCCTAATGATGGTGGTGATGATGACTAGTAGTCTGCGCGTCTTTCAGG<br>MM89: GCTAGAAATAATTTTGTTTAACTTTAAGAAGGAGATATACATATGAAAATAAAAACAGGTGCACGC
pBAD:pre^{DsbA}-MalE-TCD-mCherry	This plasmid was constructed in the following steps; (1) The pBAD:pre^{MalE}-MalE-TCD-mCherry expression plasmid was amplified by PCR using the MM88 / MM143 primers. These primers swap the MalE signal peptide with the DsbA signal peptide. (2) PCR products were *DpnI* treated and gel-purified, then ligated using the *in vivo* DNA assembly method in the *E. coli* MC1061 strain [4].	MM88: CCATCCTAATGATGGTGGTGATGATGACTAGTAGTCTGCGCGTCTTTCAGG<br>MM143: TAGCGTTTAGCGCATCGGCGAAAATCGAAGAAGGTAAACTGG
pBAD:Δss-MalE-TCD-mCherry	This plasmid was constructed in the following steps; (1) The pBAD:pre^{MalE}-MalE-TCD-mCherry was amplified by PCR using the ST8 / MM101 primers. (2) The resulting PCR product was *DpnI* treated and gel-purified, then ligated using the *in vivo* DNA assembly method in the *E. coli* MC1061 strain [4].	ST8: ATGTATATCTCCTTCTTAAAGTTAAAC<br>MM101: AGATATACATATGAAAATCGAAGAAGGTAAACTGG
pBAD:pre^{MalE}-scFv-TCD-mCherry	This plasmid was constructed in the following steps; (1) The pBAD:pre^{PelB}-hGH-TCD-mCherry expression plasmid [5] was amplified by PCR using the ST8 / ST9 primers. (2) The coding sequence for pre^{MalE}-scFv was amplified by PCR from pBAD:pre^{MalE}-scFv (obtained from Max Lindow, Stockholm University) using the ST5 / MM89 primers. (3) PCR products were *DpnI* treated and gel-purified,	ST8: ATGTATATCTCCTTCTTAAAGTTAAAC<br>ST9: ACTAGTCATCATCACCACCATCATTAG<br>ST5: CATCCTAATGATGGTGGTGATGATGACTAGTGCGTTTGATCTCCACTTTGGTGC<br>MM89: GCTAGAAATAATTTTGTTTAACTTTAAGAAGGAGATATACATATGAAAATAAAAACAGGTGCACGC

	then ligated using the *in vivo* DNA assembly method in the *E. coli* MC1061 strain [4]	
pBAD:pre^{DsbA}-scFv-TCD-mCherry	This plasmid was constructed in the following steps; (1) The pBAD:pre^{MalE}-scFv-TCD-mCherry expression plasmid was amplified by PCR using the ST9 / ST20 primers. (2) The coding sequence for pre^{DsbA}-scFv was amplified by PCR from pBAD:pre^{DsbA}-scFv (obtained from Max Lindow, Stockholm University) using the ST5 / ST16 primers. (3) PCR products were *DpnI* treated and gel-purified, then ligated using the *in vivo* DNA assembly method in the *E. coli* MC1061 strain [4]	ST5: CATCCTAATGATGGTGGTGATGATGACTAGTGCGTTTGATCTCCACTTTGGTGC<br><br>ST9: ACTAGTCATCATCACCACCATCATTAG<br><br>ST16: GAAGGAGATTAACGTATGAAAAAGATTTGGCTGGCGC<br><br>ST20: ACGTTAATCTCCTTCTTAAAGTTAAAC
pBAD:Δss-scFv-TCD-mCherry	This plasmid was constructed in the following steps; (1) The pBAD:pre^{MalE}-scFv-TCD-mCherry was amplified by PCR using the ST8 / MM97 primers. (2) The resulting PCR product was *DpnI* treated and gel-purified, then ligated using the *in vivo* DNA assembly method in the *E. coli* MC1061 strain [4].	ST8: ATGTATATCTCCTTCTTAAAGTTAAAC<br><br>MM97: GAAGGAGATATACATATGGAAGTGCAGCTGGTCGAATC
pBAD:pre^{MalE}-hGH-TCD-mCherry	This plasmid was constructed in the following steps; (1) The pBAD-pre^{PelB}-hGH expression plasmid [5] was amplified by PCR using the ST1 / ST2 primers. (2) The coding sequence for mCherry was amplified by PCR from pBAD-mCherry [7] using the ST3 / ST4 primers. This strategy simultaneously inserted the TCD. (3) PCR products were *DpnI* treated and gel-purified, then ligated using the *in vivo* DNA assembly method in the *E. coli* MC1061 strain [4]. (4) To replace the pelB signal sequence with the MalE signal sequence, the resulting plasmid was amplified by PCR using the ST23 / ST27 primers. (5) PCR products were *DpnI* treated and gel-purified, then ligated using the *in vivo* DNA assembly method in the *E. coli* MC1061 strain [4]	ST1: CCATCCTAATGATGGTGGTGATGATGACTAGTGAAACCGCAGCTACCCTCAAC<br><br>ST2: CGGCATGGACGAGCTGTACAAGTGAAAGCTTACGGTCTCCAGC<br><br>ST3: TCACCACCATCATTAGGATGGTGGTGATGATAATGATGAGCAAGGGCGAGGAGG<br><br>ST4: GCTGGAGACCGTAAGCTTTCACTTGTACAGCTCGTCCATGCCGC<br><br>ST23: CCTCGCATTATCCGCATTAACGACGATGATGTTTTCCGCCTCGGCTCTCGCCTTTCCGACCATCCCGCTG<br><br>ST27: CGTCGTTAATGCGGATAATGCGAGGATGCGTGCACCTGTTTTATTTTCATATGTATATCTCCTTCTTAAAGTTAAAC
pBAD:Mth1-TCD-mScarlet-I3	This plasmid was constructed in three steps; (1) The pBAD:Mth1-TCD-mCherry expression was amplified by PCR using the MM162 / MM176 primers. (2) The coding sequence for mScarlet-I3 was amplified from pBAD:mScarlet-I3 (see Table S6) using the MM177 / MM178 primers. (3) PCR products were *DpnI* treated and gel-purified. Fragments were then ligated using the *in vivo* DNA	MM162: AAGCTTACGGTCTCCAGCTTGGC<br><br>MM176: TATCATCACCACCATCCTAATGATGGTG<br><br>MM177: TCACCACCATCATTAGGATGGTGGTGATGATAATGGATAGCACCGAGGCAGTGATCAAGG

	assembly method in the *E. coli* MC1061 strain [4].	MM178:<br>CAAGCTGGAGACCGTAAGCTTTTAGGAGCCACCGGAGCCGCCG
pBAD:Δss-MalE-TCD-mScarlet-I3	This plasmid was constructed in three steps; (1) The pBAD:Δss-MalE-TCD-mCherry expression was amplified by PCR using the MM162 / MM176 primers. (2) The coding sequence for mScarlet-I3 was amplified from pBAD:mScarlet-I3 (see Table S6) using the MM177 / MM178 primers. (3) PCR products were *DpnI* treated and gel-purified. Fragments were then ligated using the *in vivo* DNA assembly method in the *E. coli* MC1061 strain [4].	MM162:<br>AAGCTTACGGTCTCCAGCTTGGC<br><br>MM176:<br>TATCATCACCACCATCCTAATGATGGTG<br><br>MM177:<br>TCACCACCATCATTAGGATGGTGGTGATGATAATGGATAGCACCGAGGCAGTGATCAAGG<br><br>MM178:<br>CAAGCTGGAGACCGTAAGCTTTTAGGAGCCACCGGAGCCGCCG
pBAD:pre^{MalE}-MalE-TCD-mScarlet-I3	This plasmid was constructed in three steps; (1) The pBAD:pre^{MalE}-MalE-TCD-mCherry expression was amplified by PCR using the MM162 / MM176 primers. (2) The coding sequence for mScarlet-I3 was amplified from pBAD:mScarlet-I3 (see Table S6) using the MM177 / MM178 primers. (3) PCR products were *DpnI* treated and gel-purified. Fragments were then ligated using the *in vivo* DNA assembly method in the *E. coli* MC1061 strain [4].	MM162:<br>AAGCTTACGGTCTCCAGCTTGGC<br><br>MM176:<br>TATCATCACCACCATCCTAATGATGGTG<br><br>MM177:<br>TCACCACCATCATTAGGATGGTGGTGATGATAATGGATAGCACCGAGGCAGTGATCAAGG<br><br>MM178:<br>CAAGCTGGAGACCGTAAGCTTTTAGGAGCCACCGGAGCCGCCG
pBAD:pre^{MalE}-scFv-TCD-mScarlet-I3	This plasmid was constructed in three steps; (1) The pBAD:pre^{MalE}-scFv^{HER2}-TCD-mCherry expression was amplified by PCR using the MM162 / MM176 primers. (2) The coding sequence for mScarlet-I3 was amplified from pBAD:mScarlet-I3 (see Table S6) using the MM177 / MM178 primers. (3) PCR products were *DpnI* treated and gel-purified. Fragments were then ligated using the *in vivo* DNA assembly method in the *E. coli* MC1061 strain [4].	MM162:<br>AAGCTTACGGTCTCCAGCTTGGC<br><br>MM176:<br>TATCATCACCACCATCCTAATGATGGTG<br><br>MM177:<br>TCACCACCATCATTAGGATGGTGGTGATGATAATGGATAGCACCGAGGCAGTGATCAAGG<br><br>MM178:<br>CAAGCTGGAGACCGTAAGCTTTTAGGAGCCACCGGAGCCGCCG

Table S6. Sequences of the expression plasmids used to measure maturation and degradation of fluorescent proteins. *For pBAD:GFPmut3, pBAD:GFPmut3$_{ASV}$* and *pBAD:GFPmut3$_{LVA}$* the entire sequence is shown. For all other expression plasmids only the sequence encoding for the fluorescent protein is shown. *The coding sequence that was expressed is marked in green, the coding sequence for the ASV degradation tag in orange and the coding sequence the LVA degradation tag in blue.*

**Expression plasmid**	**Linear sequence**
pBAD:GFPmut3	AAGAAACCAATTGTCCATATTGCATCAGACATTGCCGTCACTGCGTCTTTTACTGGCTCTTCTCGCTAAC<br>CAAACCGGTAACCCCGCTTATTAAAAGCATTCTGTAACAAAGCGGGACCAAAGCCATGACAAAAACG<br>CGTAACAAAAGTGTCTATAATCACGGCAGAAAAGTCCACATTGATTATTTGCACGGCGTCACACTTTG<br>CTATGCCATAGCATTTTTATCCATAAGATTAGCGGATCCTACCTGACGCTTTTTATCGCAACTCTCTACT<br>GTTTCTCCATACCCGTTTTTTTGGGCTAGAAATAATTTTGTTTAACTTTAAGAAGGAGATATACATATGC<br>GTAAAGGAGAAGAACTTTTCACTGGAGTTGTCCCAATTCTTGTTGAATTAGATGGTGATGTTAATGGG<br>CACAAATTTTCTGTCAGTGGAGAGGGTGAAGGTGATGCAACATACGGAAAACTTACCCTTAAATTTAT<br>TTGCACTACTGGAAAACTACCTGTTCCATGGCCAACACTTGTCACTACTTTCGGTTATGGTGTTCAATG<br>CTTTGCGAGATACCCAGATCATATGAAACAGCATGACTTTTTCAAGAGTGCCATGCCCGAAGGTTATG<br>TACAGGAAAGAACTATATTTTTCAAAGATGACGGGAACTACAAGACACGTGCTGAAGTCAAGTTTGA<br>AGGTGATACCCTTGTTAATAGAATCGAGTTAAAAGGTATTGATTTTAAAGAAGATGGAAACATTCTTG<br>GACACAAATTGGAATACAACTATAACTCACACAATGTATACATCATGGCAGACAAACAAAAGAATGG<br>AATCAAAGTTAACTTCAAAATTAGACACAACATTGAAGATGGAAGCGTTCAACTAGCAGACCATTATC<br>AACAAAATACTCCAATTGGCGATGGCCCTGTCCTTTTACCAGACAACCATTACCTGTCCACACAATCTG<br>CCCTTTCGAAAGATCCCAACGAAAAGAGAGACCACATGGTCCTTCTTGAGTTTGTAACAGCTGCTGGG<br>ATTACACATGGCATGGATGAACTATACAAATAAAAGCTTACGGTCTCCAGCTTGGCTGTTTTGGCGGA<br>TGAGAGAAGATTTTCAGCCTGATACAGATTAAATCAGAACGCAGAAGCGGTCTGATAAAACAGAATT<br>TGCCTGGCGGCAGTAGCGCGGTGGTCCCACCTGACCCCATGCCGAACTCAGAAGTGAAACGCCGTAG<br>CGCCGATGGTAGTGTGGGGTCTCCCCATGCGAGAGTAGGGAACTGCCAGGCATCAAATAAAACGAA<br>AGGCTCAGTCGAAAGACTGGGCCTTTCGTTTTATCTGTTGTTTGTCGGTGAACGCTCTCCTGAGTAGG<br>ACAAATCCGCCGGGAGCGGATTTGAACGTTGCGAAGCAACGGCCCGGAGGGTGGCGGGCAGGACG<br>CCCGCCATAAACTGCCAGGCATCAAATTAAGCAGAAGGCCATCCTGACGGATGGCCTTTTTGCGTTTC<br>TACAAACTCTCATGAACAATAAAACTGTCTGCTTACATAAACAGTAATACAAGGGGTGTTATGAGCCA<br>TATTCAACGGGAAACGTCTTGCTCTAGGCCGCGATTAAATTCCAACATGGATGCTGATTTATATGGGT<br>ATAAATGGGCTCGCGATAATGTCGGGCAATCAGGTGCGACAATCTATCGATTGTATGGGAAGCCCGA<br>TGCGCCAGAGTTGTTTCTGAAACATGGCAAAGGTAGCGTTGCCAATGATGTTACAGATGAGATGGTC<br>AGACTAAACTGGCTGACGGAATTTATGCCTCTTCCGACCATCAAGCATTTTATCCGTACTCCTGATGAT<br>GCATGGTTACTCACCACTGCGATCCCCGGGAAAACAGCATTCCAGGTATTAGAAGAATATCCTGATTC<br>AGGTGAAAATATTGTTGATGCGCTGGCAGTGTTCCTGCGCCGGTTGCATTCGATTCCTGTTTGTAATTG<br>TCCTTTTAACAGCGATCGCGTATTTCGTCTCGCTCAGGCGCAATCACGAATGAATAACGGTTTGGTTGA<br>TGCGAGTGATTTTGATGACGAGCGTAATGGCTGGCCTGTTGAACAAGTCTGGAAAGAAATGCATAAA<br>CTTTTGCCATTCTCACCGGATTCAGTCGTCACTCATGGTGATTTCTCACTTGATAACCTTATTTTTGACG<br>AGGGGAAATTAATAGGTTGTATTGATGTTGGACGAGTCGGAATCGCAGACCGATACCAGGATCTTGC<br>CATCCTATGGAACTGCCTCGGTGAGTTTTCTCCTTCATTACAGAAACGGCTTTTTCAAAAATATGGTATT<br>GATAATCCTGATATGAATAAATTGCAGTTTCATTTGATGCTCGATGAGTTTTTCTAAGCTGTCAGACCA<br>AGTTTACTCATATATACTTTAGATTGATTTAAAACTTCATTTTTAATTTAAAAGGATCTAGGTGAAGATC<br>CTTTTTGATAATCTCATGACCAAAATCCCTTAACGTGAGTTTTCGTTCCACTGAGCGTCAGACCCCGTA<br>GAAAAGATCAAAGGATCTTCTTGAGATCCTTTTTTTCTGCGCGTAATCTGCTGCTTGCAAACAAAAAAA<br>CCACCGCTACCAGCGGTGGTTTGTTTGCCGGATCAAGAGCTACCAACTCTTTTTCCGAAGGTAACTGG<br>CTTCAGCAGAGCGCAGATACCAAATACTGTCCTTCTAGTGTAGCCGTAGTTAGGCCACCACTTCAAGA<br>ACTCTGTAGCACCGCCTACATACCTCGCTCTGCTAATCCTGTTACCAGTGGCTGCTGCCAGTGGCGATA<br>AGTCGTGTCTTACCGGGTTGGACTCAAGACGATAGTTACCGGATAAGGCGCAGCGGTCGGGCTGAAC<br>GGGGGGTTCGTGCACACAGCCCAGCTTGGAGCGAACGACCTACACCGAACTGAGATACCTACAGCGT<br>GAGCTATGAGAAAGCGCCACGCTTCCCGAAGGGAGAAAGGCGGACAGGTATCCGGTAAGCGGCAG<br>GGTCGGAACAGGAGAGCGCACGAGGGAGCTTCCAGGGGGAAACGCCTGGTATCTTTATAGTCCTGTC<br>GGGTTTCGCCACCTCTGACTTGAGCGTCGATTTTTGTGATGCTCGTCAGGGGGGCGGAGCCTATGGA<br>AAAACGCCAGCAACGCGGCCTTTTTACGGTTCCTGGCCTTTTGCTGGCCTTTTGCTCACATGTTCTTTCC<br>TGCGTTATCCCCTGATTCTGTGGATAACCGTATTACCGCCTTTGAGTGAGCTGATACCGCTCGCCGCAG<br>CCGAACGACCGAGCGCAGCGAGTCAGTGAGCGAGGAAGCGGAAGAGCGCCTGATGCGGTATTTTCT<br>CCTTACGCATCTGTGCGGTATTTCACACCGCATATGGTGCACTCTCAGTACAATCTGCTCTGATGCCGC<br>ATAGTTAAGCCAGTATACACTCCGCTATCGCTACGTGACTGGGTCATGGCTGCGCCCCGACACCCGCC<br>AACACCCGCTGACGCGCCCTGACGGGCTTGTCTGCTCCCGGCATCCGCTTACAGACAAGCTGTGACCG<br>TCTCCGGGAGCTGCATGTGTCAGAGGTTTTCACCGTCATCACCGAAACGCGCGAGGCAGCAGATCAA<br>TTCGCGCGCGAAGGCGAAGCGGCATGCATAATGTGCCTGTCAAATGGACGAAGCAGGGATTCTGCAA

	ACCCTATGCTACTCCGTCAAGCCGTCAATTGTCTGATTCGTTACCAATTATGACAACTTGACGGCTACA<br>TCATTCACTTTTTCTTCACAACCGGCACGGAACTCGCTCGGGCTGGCCCCGGTGCATTTTTTAAATACC<br>CGCGAGAAATAGAGTTGATCGTCAAAACCAACATTGCGACCGACGGTGGCGATAGGCATCCGGGTG<br>GTGCTCAAAAGCAGCTTCGCCTGGCTGATACGTTGGTCCTCGCGCCAGCTTAAGACGCTAATCCCTAA<br>CTGCTGGCGGAAAAGATGTGACAGACGCGACGGCGACAAGCAAACATGCTGTGCGACGCTGGCGAT<br>ATCAAAATTGCTGTCTGCCAGGTGATCGCTGATGTACTGACAAGCCTCGCGTACCCGATTATCCATCG<br>GTGGATGGAGCGACTCGTTAATCGCTTCCATGCGCCGCAGTAACAATTGCTCAAGCAGATTTATCGCC<br>AGCAGCTCCGAATAGCGCCCTTCCCCTTGCCCGGCGTTAATGATTTGCCCAAACAGGTCGCTGAAATG<br>CGGCTGGTGCGCTTCATCCGGGCGAAAGAACCCCGTATTGGCAAATATTGACGGCCAGTTAAGCCATT<br>CATGCCAGTAGGCGCGCGGACGAAAGTAAACCCACTGGTGATACCATTCGCGAGCCTCCGGATGACG<br>ACCGTAGTGATGAATCTCTCCTGGCGGGAACAGCAAAATATCACCCGGTCGGCAAACAAATTCTCGTC<br>CCTGATTTTTCACCACCCCCTGACCGCGAATGGTGAGATTGAGAATATAACCTTTCATTCCCAGCGGTC<br>GGTCGATAAAAAAATCGAGATAACCGTTGGCCTCAATCGGCGTTAAACCCGCCACCAGATGGGCATT<br>AAACGAGTATCCCGGCAGCAGGGGATCATTTTGCGCTTCAGCCATACTTTTCATACTCCCGCCATTCAG<br>AG
pBAD:GFPmut3$_{ASV}$	AAGAAACCAATTGTCCATATTGCATCAGACATTGCCGTCACTGCGTCTTTTACTGGCTCTTCTCGCTAAC<br>CAAACCGGTAACCCCGCTTATTAAAAGCATTCTGTAACAAAGCGGGACCAAAGCCATGACAAAAACG<br>CGTAACAAAAGTGTCTATAATCACGGCAGAAAAGTCCACATTGATTATTTGCACGGCGTCACACTTTG<br>CTATGCCATAGCATTTTTATCCATAAGATTAGCGGATCCTACCTGACGCTTTTTATCGCAACTCTCTACT<br>GTTTCTCCATACCCGTTTTTTTGGGCTAGAAATAATTTTGTTTAACTTTAAGAAGGAGATATACATATGC<br>GTAAAGGAGAAGAACTTTTCACTGGAGTTGTCCCAATTCTTGTTGAATTAGATGGTGATGTTAATGGG<br>CACAAATTTTCTGTCAGTGGAGAGGGTGAAGGTGATGCAACATACGGAAAACTTACCCTTAAATTTAT<br>TTGCACTACTGGAAAACTACCTGTTCCATGGCCAACACTTGTCACTACTTTCGGTTATGGTGTTCAATG<br>CTTTGCGAGATACCCAGATCATATGAAACAGCATGACTTTTTCAAGAGTGCCATGCCCGAAGGTTATG<br>TACAGGAAAGAACTATATTTTTCAAAGATGACGGGAACTACAAGACACGTGCTGAAGTCAAGTTTGA<br>AGGTGATACCCTTGTTAATAGAATCGAGTTAAAAGGTATTGATTTTAAAGAAGATGGAAACATTCTTG<br>GACACAAATTGGAATACAACTATAACTCACACAATGTATACATCATGGCAGACAAACAAAAGAATGG<br>AATCAAAGTTAACTTCAAAATTAGACACAACATTGAAGATGGAAGCGTTCAACTAGCAGACCATTATC<br>AACAAAATACTCCAATTGGCGATGGCCCTGTCCTTTTACCAGACAACCATTACCTGTCCACACAATCTG<br>CCCTTTCGAAAGATCCCAACGAAAAGAGAGACCACATGGTCCTTCTTGAGTTTGTAACAGCTGCTGGG<br>ATTACACATGGCATGGATGAACTATACAAAAGGCCTGCAGCAAACGACGAAAACTACGCTGCATCAG<br>TTTAAAAGCTTACGGTCTCCAGCTTGGCTGTTTTGGCGGATGAGAGAAGATTTTCAGCCTGATACAGA<br>TTAAATCAGAACGCAGAAGCGGTCTGATAAAACAGAATTTGCCTGGCGGCAGTAGCGCGGTGGTCCC<br>ACCTGACCCCATGCCGAACTCAGAAGTGAAACGCCGTAGCGCCGATGGTAGTGTGGGGTCTCCCCAT<br>GCGAGAGTAGGGAACTGCCAGGCATCAAATAAAACGAAAGGCTCAGTCGAAAGACTGGGCCTTTCGT<br>TTTATCTGTTGTTTGTCGGTGAACGCTCTCCTGAGTAGGACAAATCCGCCGGGAGCGGATTTGAACGT<br>TGCGAAGCAACGGCCCGGAGGGTGGCGGGCAGGACGCCCGCCATAAACTGCCAGGCATCAAATTAA<br>GCAGAAGGCCATCCTGACGGATGGCCTTTTTGCGTTTCTACAAACTCTCATGAACAATAAAACTGTCTG<br>CTTACATAAACAGTAATACAAGGGGTGTTATGAGCCATATTCAACGGGAAACGTCTTGCTCTAGGCCG<br>CGATTAAATTCCAACATGGATGCTGATTTATATGGGTATAAATGGGCTCGCGATAATGTCGGGCAATC<br>AGGTGCGACAATCTATCGATTGTATGGGAAGCCCGATGCGCCAGAGTTGTTTCTGAAACATGGCAAA<br>GGTAGCGTTGCCAATGATGTTACAGATGAGATGGTCAGACTAAACTGGCTGACGGAATTTATGCCTCT<br>TCCGACCATCAAGCATTTTATCCGTACTCCTGATGATGCATGGTTACTCACCACTGCGATCCCCGGGAA<br>AACAGCATTCCAGGTATTAGAAGAATATCCTGATTCAGGTGAAAATATTGTTGATGCGCTGGCAGTGT<br>TCCTGCGCCGGTTGCATTCGATTCCTGTTTGTAATTGTCCTTTTAACAGCGATCGCGTATTTCGTCTCGC<br>TCAGGCGCAATCACGAATGAATAACGGTTTGGTTGATGCGAGTGATTTTGATGACGAGCGTAATGGC<br>TGGCCTGTTGAACAAGTCTGGAAAGAAATGCATAAACTTTTGCCATTCTCACCGGATTCAGTCGTCACT<br>CATGGTGATTTCTCACTTGATAACCTTATTTTTGACGAGGGGAAATTAATAGGTTGTATTGATGTTGGA<br>CGAGTCGGAATCGCAGACCGATACCAGGATCTTGCCATCCTATGGAACTGCCTCGGTGAGTTTTCTCC<br>TTCATTACAGAAACGGCTTTTTCAAAAATATGGTATTGATAATCCTGATATGAATAAATTGCAGTTTCA<br>TTTGATGCTCGATGAGTTTTTCTAAGCTGTCAGACCAAGTTTACTCATATATACTTTAGATTGATTTAA<br>ACTTCATTTTTAATTTAAAAGGATCTAGGTGAAGATCCTTTTTGATAATCTCATGACCAAAATCCCTTAA<br>CGTGAGTTTTCGTTCCACTGAGCGTCAGACCCCGTAGAAAAGATCAAAGGATCTTCTTGAGATCCTTTT<br>TTTCTGCGCGTAATCTGCTGCTTGCAAACAAAAAAACCACCGCTACCAGCGGTGGTTTGTTTGCCGGA<br>TCAAGAGCTACCAACTCTTTTTCCGAAGGTAACTGGCTTCAGCAGAGCGCAGATACCAAATACTGTCC<br>TTCTAGTGTAGCCGTAGTTAGGCCACCACTTCAAGAACTCTGTAGCACCGCCTACATACCTCGCTCTGC<br>TAATCCTGTTACCAGTGGCTGCTGCCAGTGGCGATAAGTCGTGTCTTACCGGGTTGGACTCAAGACGA<br>TAGTTACCGGATAAGGCGCAGCGGTCGGGCTGAACGGGGGGTTCGTGCACACAGCCCAGCTTGGAG<br>CGAACGACCTACACCGAACTGAGATACCTACAGCGTGAGCTATGAGAAAGCGCCACGCTTCCCGAAG<br>GGAGAAAGGCGGACAGGTATCCGGTAAGCGGCAGGGTCGGAACAGGAGAGCGCACGAGGGAGCTT<br>CCAGGGGGAAACGCCTGGTATCTTTATAGTCCTGTCGGGTTTCGCCACCTCTGACTTGAGCGTCGATT<br>TTTGTGATGCTCGTCAGGGGGGCGGAGCCTATGGAAAAACGCCAGCAACGCGGCCTTTTTACGGTTC

	CTGGCCTTTTGCTGGCCTTTTGCTCACATGTTCTTTCCTGCGTTATCCCCTGATTCTGTGGATAACCGTA<br>TTACCGCCTTTGAGTGAGCTGATACCGCTCGCCGCAGCCGAACGACCGAGCGCAGCGAGTCAGTGAG<br>CGAGGAAGCGGAAGAGCGCCTGATGCGGTATTTTCTCCTTACGCATCTGTGCGGTATTTCACACCGCA<br>TATGGTGCACTCTCAGTACAATCTGCTCTGATGCCGCATAGTTAAGCCAGTATACACTCCGCTATCGCT<br>ACGTGACTGGGTCATGGCTGCGCCCCGACACCCGCCAACACCCGCTGACGCGCCCTGACGGGCTTGT<br>CTGCTCCCGGCATCCGCTTACAGACAAGCTGTGACCGTCTCCGGGAGCTGCATGTGTCAGAGGTTTTC<br>ACCGTCATCACCGAAACGCGCGAGGCAGCAGATCAATTCGCGCGCGAAGGCGAAGCGGCATGCATA<br>ATGTGCCTGTCAAATGGACGAAGCAGGGATTCTGCAAACCCTATGCTACTCCGTCAAGCCGTCAATTG<br>TCTGATTCGTTACCAATTATGACAACTTGACGGCTACATCATTCACTTTTTCTTCACAACCGGCACGGAA<br>CTCGCTCGGGCTGGCCCCGGTGCATTTTTTAAATACCCGCGAGAAATAGAGTTGATCGTCAAAACCAA<br>CATTGCGACCGACGGTGGCGATAGGCATCCGGGTGGTGCTCAAAAGCAGCTTCGCCTGGCTGATACG<br>TTGGTCCTCGCGCCAGCTTAAGACGCTAATCCCTAACTGCTGGCGGAAAAGATGTGACAGACGCGAC<br>GGCGACAAGCAAACATGCTGTGCGACGCTGGCGATATCAAAATTGCTGTCTGCCAGGTGATCGCTGA<br>TGTACTGACAAGCCTCGCGTACCCGATTATCCATCGGTGGATGGAGCGACTCGTTAATCGCTTCCATG<br>CGCCGCAGTAACAATTGCTCAAGCAGATTTATCGCCAGCAGCTCCGAATAGCGCCCTTCCCCTTGCCC<br>GGCGTTAATGATTTGCCCAAACAGGTCGCTGAAATGCGGCTGGTGCGCTTCATCCGGGCGAAAGAAC<br>CCCGTATTGGCAAATATTGACGGCCAGTTAAGCCATTCATGCCAGTAGGCGCGCGGACGAAAGTAAA<br>CCCACTGGTGATACCATTCGCGAGCCTCCGGATGACGACCGTAGTGATGAATCTCTCCTGGCGGGAAC<br>AGCAAAATATCACCCGGTCGGCAAACAAATTCTCGTCCCTGATTTTTCACCACCCCCTGACCGCGAATG<br>GTGAGATTGAGAATATAACCTTTCATTCCCAGCGGTCGGTCGATAAAAAAATCGAGATAACCGTTGGC<br>CTCAATCGGCGTTAAACCCGCCACCAGATGGGCATTAAACGAGTATCCCGGCAGCAGGGGATCATTTT<br>GCGCTTCAGCCATACTTTTCATACTCCCGCCATTCAGAG
pBAD:GFPmut3$_{LVA}$	AAGAAACCAATTGTCCATATTGCATCAGACATTGCCGTCACTGCGTCTTTTACTGGCTCTTCTCGCTAAC<br>CAAACCGGTAACCCCGCTTATTAAAAGCATTCTGTAACAAAGCGGGACCAAAGCCATGACAAAAACG<br>CGTAACAAAAGTGTCTATAATCACGGCAGAAAAGTCCACATTGATTATTTGCACGGCGTCACACTTTG<br>CTATGCCATAGCATTTTTATCCATAAGATTAGCGGATCCTACCTGACGCTTTTTATCGCAACTCTCTACT<br>GTTTCTCCATACCCGTTTTTTTGGGCTAGAAATAATTTTGTTTAACTTTAAGAAGGAGATATACATATGC<br>GTAAAGGAGAAGAACTTTTCACTGGAGTTGTCCCAATTCTTGTTGAATTAGATGGTGATGTTAATGGG<br>CACAAATTTTCTGTCAGTGGAGAGGGTGAAGGTGATGCAACATACGGAAAACTTACCCTTAAATTTAT<br>TTGCACTACTGGAAAACTACCTGTTCCATGGCCAACACTTGTCACTACTTTCGGTTATGGTGTTCAATG<br>CTTTGCGAGATACCCAGATCATATGAAACAGCATGACTTTTTCAAGAGTGCCATGCCCGAAGGTTATG<br>TACAGGAAAGAACTATATTTTTCAAAGATGACGGGAACTACAAGACACGTGCTGAAGTCAAGTTTGA<br>AGGTGATACCCTTGTTAATAGAATCGAGTTAAAAGGTATTGATTTTAAAGAAGATGGAAACATTCTTG<br>GACACAAATTGGAATACAACTATAACTCACACAATGTATACATCATGGCAGACAAACAAAAGAATGG<br>AATCAAAGTTAACTTCAAAATTAGACACAACATTGAAGATGGAAGCGTTCAACTAGCAGACCATTATC<br>AACAAAATACTCCAATTGGCGATGGCCCTGTCCTTTTACCAGACAACCATTACCTGTCCACACAATCTG<br>CCCTTTCGAAAGATCCCAACGAAAAGAGAGACCACATGGTCCTTCTTGAGTTTGTAACAGCTGCTGGG<br>ATTACACATGGCATGGATGAACTATACAAAAGGCCTGCAGCAAACGACGAAAACTACGCTTTAGTAG<br>CTTAAAAGCTTACGGTCTCCAGCTTGGCTGTTTTGGCGGATGAGAGAAGATTTTCAGCCTGATACAGA<br>TTAAATCAGAACGCAGAAGCGGTCTGATAAAACAGAATTTGCCTGGCGGCAGTAGCGCGGTGGTCCC<br>ACCTGACCCCATGCCGAACTCAGAAGTGAAACGCCGTAGCGCCGATGGTAGTGTGGGGTCTCCCCAT<br>GCGAGAGTAGGGAACTGCCAGGCATCAAATAAAACGAAAGGCTCAGTCGAAAGACTGGGCCTTTCGT<br>TTTATCTGTTGTTTGTCGGTGAACGCTCTCCTGAGTAGGACAAATCCGCCGGGAGCGGATTTGAACGT<br>TGCGAAGCAACGGCCCGGAGGGTGGCGGGCAGGACGCCCGCCATAAACTGCCAGGCATCAAATTAA<br>GCAGAAGGCCATCCTGACGGATGGCCTTTTTGCGTTTCTACAAACTCTCATGAACAATAAAACTGTCTG<br>CTTACATAAACAGTAATACAAGGGGTGTTATGAGCCATATTCAACGGGAAACGTCTTGCTCTAGGCCG<br>CGATTAAATTCCAACATGGATGCTGATTTATATGGGTATAAATGGGCTCGCGATAATGTCGGGCAATC<br>AGGTGCGACAATCTATCGATTGTATGGGAAGCCCGATGCGCCAGAGTTGTTTCTGAAACATGGCAAA<br>GGTAGCGTTGCCAATGATGTTACAGATGAGATGGTCAGACTAAACTGGCTGACGGAATTTATGCCTCT<br>TCCGACCATCAAGCATTTTATCCGTACTCCTGATGATGCATGGTTACTCACCACTGCGATCCCCGGGAA<br>AACAGCATTCCAGGTATTAGAAGAATATCCTGATTCAGGTGAAAATATTGTTGATGCGCTGGCAGTGT<br>TCCTGCGCCGGTTGCATTCGATTCCTGTTTGTAATTGTCCTTTTAACAGCGATCGCGTATTTCGTCTCGC<br>TCAGGCGCAATCACGAATGAATAACGGTTTGGTTGATGCGAGTGATTTTGATGACGAGCGTAATGGC<br>TGGCCTGTTGAACAAGTCTGGAAAGAAATGCATAAACTTTTGCCATTCTCACCGGATTCAGTCGTCACT<br>CATGGTGATTTCTCACTTGATAACCTTATTTTTGACGAGGGGAAATTAATAGGTTGTATTGATGTTGGA<br>CGAGTCGGAATCGCAGACCGATACCAGGATCTTGCCATCCTATGGAACTGCCTCGGTGAGTTTTCTCC<br>TTCATTACAGAAACGGCTTTTTCAAAAATATGGTATTGATAATCCTGATATGAATAAATTGCAGTTTCA<br>TTTGATGCTCGATGAGTTTTTCTAAGCTGTCAGACCAAGTTTACTCATATATACTTTAGATTGATTTAAA<br>ACTTCATTTTTAATTTAAAAGGATCTAGGTGAAGATCCTTTTTGATAATCTCATGACCAAAATCCCTTAA<br>CGTGAGTTTTCGTTCCACTGAGCGTCAGACCCCGTAGAAAAGATCAAAGGATCTTCTTGAGATCCTTTT<br>TTTCTGCGCGTAATCTGCTGCTTGCAAACAAAAAAACCACCGCTACCAGCGGTGGTTTGTTTGCCGGA<br>TCAAGAGCTACCAACTCTTTTTCCGAAGGTAACTGGCTTCAGCAGAGCGCAGATACCAAATACTGTCC

	TTCTAGTGTAGCCGTAGTTAGGCCACCACTTCAAGAACTCTGTAGCACCGCCTACATACCTCGCTCTGC<br>TAATCCTGTTACCAGTGGCTGCTGCCAGTGGCGATAAGTCGTGTCTTACCGGGTTGGACTCAAGACGA<br>TAGTTACCGGATAAGGCGCAGCGGTCGGGCTGAACGGGGGGTTCGTGCACACAGCCCAGCTTGGAG<br>CGAACGACCTACACCGAACTGAGATACCTACAGCGTGAGCTATGAGAAAGCGCCACGCTTCCCGAAG<br>GGAGAAAGGCGGACAGGTATCCGGTAAGCGGCAGGGTCGGAACAGGAGAGCGCACGAGGGAGCTT<br>CCAGGGGGAAACGCCTGGTATCTTTATAGTCCTGTCGGGTTTCGCCACCTCTGACTTGAGCGTCGATT<br>TTTGTGATGCTCGTCAGGGGGGCGGAGCCTATGGAAAAACGCCAGCAACGCGGCCTTTTTACGGTTC<br>CTGGCCTTTTGCTGGCCTTTTGCTCACATGTTCTTTCCTGCGTTATCCCCTGATTCTGTGGATAACCGTA<br>TTACCGCCTTTGAGTGAGCTGATACCGCTCGCCGCAGCCGAACGACCGAGCGCAGCGAGTCAGTGAG<br>CGAGGAAGCGGAAGAGCGCCTGATGCGGTATTTTCTCCTTACGCATCTGTGCGGTATTTCACACCGCA<br>TATGGTGCACTCTCAGTACAATCTGCTCTGATGCCGCATAGTTAAGCCAGTATACACTCCGCTATCGCT<br>ACGTGACTGGGTCATGGCTGCGCCCCGACACCCGCCAACACCCGCTGACGCGCCCTGACGGGCTTGT<br>CTGCTCCCGGCATCCGCTTACAGACAAGCTGTGACCGTCTCCGGGAGCTGCATGTGTCAGAGGTTTTC<br>ACCGTCATCACCGAAACGCGCGAGGCAGCAGATCAATTCGCGCGCGAAGGCGAAGCGGCATGCATA<br>ATGTGCCTGTCAAATGGACGAAGCAGGGATTCTGCAAACCCTATGCTACTCCGTCAAGCCGTCAATTG<br>TCTGATTCGTTACCAATTATGACAACTTGACGGCTACATCATTCACTTTTTCTTCACAACCGGCACGGAA<br>CTCGCTCGGGCTGGCCCCGGTGCATTTTTTAAATACCCGCGAGAAATAGAGTTGATCGTCAAAACCAA<br>CATTGCGACCGACGGTGGCGATAGGCATCCGGGTGGTGCTCAAAAGCAGCTTCGCCTGGCTGATACG<br>TTGGTCCTCGCGCCAGCTTAAGACGCTAATCCCTAACTGCTGGCGGAAAAGATGTGACAGACGCGAC<br>GGCGACAAGCAAACATGCTGTGCGACGCTGGCGATATCAAAATTGCTGTCTGCCAGGTGATCGCTGA<br>TGTACTGACAAGCCTCGCGTACCCGATTATCCATCGGTGGATGGAGCGACTCGTTAATCGCTTCCATG<br>CGCCGCAGTAACAATTGCTCAAGCAGATTTATCGCCAGCAGCTCCGAATAGCGCCCTTCCCCTTGCCC<br>GGCGTTAATGATTTGCCCAAACAGGTCGCTGAAATGCGGCTGGTGCGCTTCATCCGGGCGAAAGAAC<br>CCCGTATTGGCAAATATTGACGGCCAGTTAAGCCATTCATGCCAGTAGGCGCGCGGACGAAAGTAAA<br>CCCACTGGTGATACCATTCGCGAGCCTCCGGATGACGACCGTAGTGATGAATCTCTCCTGGCGGGAAC<br>AGCAAAATATCACCCGGTCGGCAAACAAATTCTCGTCCCTGATTTTTCACCACCCCCTGACCGCGAATG<br>GTGAGATTGAGAATATAACCTTTCATTCCCAGCGGTCGGTCGATAAAAAAATCGAGATAACCGTTGGC<br>CTCAATCGGCGTTAAACCCGCCACCAGATGGGCATTAAACGAGTATCCCGGCAGCAGGGGATCATTTT<br>GCGCTTCAGCCATACTTTTCATACTCCCGCCATTCAGAG
mCherry	ATGTCGAAAGGCGAGGAGGATAACATGGCCATCATCAAGGAGTTCATGCGCTTCAAGGTGCACATGG<br>AGGGCTCCGTGAACGGCCACGAGTTCGAGATCGAGGGCGAGGGCGAGGGCCGCCCCTACGAGGGC<br>ACCCAGACCGCCAAGCTGAAGGTGACCAAGGGTGGCCCCCTGCCCTTCGCCTGGGACATCCTGTCCCC<br>TCAGTTCATGTACGGCTCCAAGGCCTACGTGAAGCACCCCGCCGACATCCCCGACTACTTGAAGCTGT<br>CCTTCCCCGAGGGCTTCAAGTGGGAGCGCGTGATGAACTTCGAGGACGGCGGCGTGGTGACCGTGA<br>CCCAGGACTCCTCCCTGCAGGACGGCGAGTTCATCTACAAGGTGAAGCTGCGCGGCACCAACTTCCCC<br>TCCGACGGCCCCGTAATGCAGAAGAAGACCATGGGCTGGGAGGCCTCCTCCGAGCGGATGTACCCCG<br>AGGACGGCGCCCTGAAGGGCGAGATCAAGCAGAGGCTGAAGCTGAAGGACGGCGGCCACTACGAC<br>GCTGAGGTCAAGACCACCTACAAGGCCAAGAAGCCCGTGCAGCTGCCCGGCGCCTACAACGTCAACA<br>TCAAGTTGGACATCACCTCCCACAACGAGGACTACACCATCGTGGAACAGTACGAACGCGCCGAGGG<br>CCGCCACTCCACCGGCGGCATGGACGAGCTGTACAAGTAA
mScarlet-I3	ATGGATAGCACCGAGGCAGTGATCAAGGAGTTCATGCGGTTCAAGGTGCACATGGAGGGCTCCATG<br>AACGGCCACGAGTTCGAGATCGAGGGCGAGGGCGAGGGCCGCCCCTACGAGGGCACCCAGACCGCC<br>AAGCTGAAGGTGACCAAGGGTGGCCCCCTGCCCTTCTCCTGGGACATCCTGTCCCCTCAGTTCATGTA<br>CGGCTCCAGGGCCTTCATCAAGCACCCCGCCGACATCCCCGACTACTGGAAGCAGTCCTTCCCCGAGG<br>GCTTCAAGTGGGAGCGCGTGATGATCTTCGAGGACGGCGGCACCGTGTCCGTGACCCAGGACACCTC<br>CCTGGAGGACGGCACCCTGATCTACAAGGTGAAGCTCCGCGGCGGCAACTTCCCTCCTGACGGCCCC<br>GTAATGCAGAAGAGGACAATGGGCTGGGAAGCATCCACCGAGCGGTTGTACCCCGAGGACGTCGTG<br>CTGAAGGGCGACATTAAGATGGCCCTGCGCCTGAAGGACGGCGGCCGCTACCTGGCGGACTTCAAG<br>ACCACCTACAAGGCCAAGAAGCCCGTGCAGATGCCCGGCGCCTTCAACATCGACCGCAAGTTGGACA<br>TCACCTCCCACAACGAGGACTACACCGTGGTGGAACAGTACGAACGCTCCGTGGCCCGCCACTCCACC<br>GGCGGCTCCGGTGGCTCCTAA
mCerulean	ATGAGTAAAGGAGAAGAACTTTTCACCGGGGTGGTGCCCATCCTGGTCGAGCTGGACGGCGACGTAA<br>ACGGCCACAAGTTCAGCGTGTCCGGCGAGGGCGAGGGCGATGCCACCTACGGCAAGCTGACCCTGA<br>AGTTCATCTGCACCACCGGCAAGCTGCCCGTGCCCTGGCCCACCCTCGTGACCACCCTGACCTGGGGC<br>GTGCAGTGCTTCGCCCGCTACCCCGACCACATGAAGCAGCACGACTTCTTCAAGTCCGCCATGCCCGA<br>AGGCTACGTCCAGGAGCGCACCATCTTCTTCAAGGACGACGGCAACTACAAGACCCGCGCCGAGGTG<br>AAGTTCGAGGGCGACACCCTGGTGAACCGCATCGAGCTGAAGGGCATCGACTTCAAGGAGGACGGC<br>AACATCCTGGGGCACAAGCTGGAGTACAACGCCATCAGCGACAACGTCTATATCACCGCCGACAAGC<br>AGAAGAACGGCATCAAGGCCAACTTCAAGATCCGCCACAACATCGAGGACGGCAGCGTGCAGCTCGC<br>CGACCACTACCAGCAGAACACCCCCATCGGCGACGGCCCCGTGCTGCTGCCCGACAACCACTACCTGA

	GCACCCAGTCCAAGCTGAGCAAAGACCCCAACGAGAAGCGCGATCACATGGTCCTGCTGGAGTTCGT GACCGCCGCCGGGATTACACTAGGCATGGATGAACTATACAAATAa

Table S7. Details on the construction of the expression plasmids used to measure maturation and degradation of fluorescent proteins.

**Plasmid**	**Construction notes**	**Primers used (5'-3')**
pBAD:GFPmut3$_{ASV}$<br>pBAD:mCherry$_{ASV}$	These plasmids were constructed in three steps. (1) For the backbone the pBAD:Mth1-TCD-mCherry expression plasmid was amplified by PCR using the ST8 / MM102 primers. (2) The respective fluorescence proteins were amplified as follows. GFPmut3 from [P$_{ibpA/fxsA}$-gfp] using the AR5 / AR6 primers. mCherry from pBAD:Mth1-TCD-mCherry using the AR5 / AR8 primers. (3) PCR products were *DpnI* treated and gel-purified, then ligated using the *in vivo* DNA assembly method in the *E. coli* MC1061 strain [4].	ST8:<br>ATGTATATCTCCTTCTTAAAGTTAAAC<br><br>MM102:<br>AAGCTTACGGTCTCCAGC<br><br>AR5:<br>GCTGGAGACCGTAAGCTTTTAAACTGATGCAGCGTAGTTTTCGTCG<br><br>AR6:<br>TTTAAGAAGGAGATATACATATGCGTAAAGGAGAAGAACTTTTCACTGG<br><br>AR8:<br>TTTAAGAAGGAGATATACATTATGTCGAAAGGCGAGGAGGATAAC
pBAD:mCerulean$_{ASV}$	This plasmid was constructed in three steps. (1) The backbone from pBAD:GFPmut3$_{ASV}$ was amplified using the ST8 / LK5 primers. (2) mCerulean was amplified from pKS1 [8] using the LK2 / LK3 primers. (3) PCR products were *DpnI* treated and gel-purified, then ligated using the *in vivo* DNA assembly method in the *E. coli* MC1061 strain [4].	ST8:<br>ATGTATATCTCCTTCTTAAAGTTAAAC<br><br>LK2:<br>CTTTAAGAAGGAGATATACATATGAGTAAAGGAGAAGAACTTTTCACTGGAGTTGTCC<br><br>LK3:<br>TTTCGTCGTTTGCTGCAGGCCTTTTGTATAGTTCATCCATGCCTAGTGTAATCCCAGC<br><br>LK5:<br>AGGCCTGCAGCAAACGACG
pBAD:GFP<br>pBAD:mCherry<br>pBAD:mCerulean	These plasmids were constructed in three steps. (1) For each fluorescent protein, the respective plasmid containing the fluorescent protein with ASV-tag (listed above) was amplified to exclude the ASV-tag using the MM162 primer and a primer for the respective protein; GFP: MM163, mCherry: MM164 and mCerulean: LK1. (2) PCR products were *DpnI* treated and gel-purified, then ligated using the *in vivo* DNA assembly method in the *E. coli* MC1061 strain [4].	MM162:<br>AAGCTTACGGTCTCCAGCTTGGC<br><br>MM163:<br>GAGACCGTAAGCTTTTATTTGTATAGTTCATCCATGCCATGTGTAATCCC<br><br>MM164:<br>GAGACCGTAAGCTTTTACTTGTACAGCTCGTCCATGCCGC<br><br>LK1:<br>CTGGAGACCGTAAGCTTTTATTTGTATAGTTCATCCATGCCTAGTGTAATCCCAGC
pBAD:mScarlet-I3	This plasmid was constructed in three steps. (1) The backbone from pBAD:GFPmut3$_{ASV}$ was amplified using the ST8 / MM102 primers. (2) The coding sequence for mScarlet-I3 with overhangs matching the plasmid backbone was synthesized by GenScript. (3) PCR products were *DpnI* treated and gel-purified. Fragments were then ligated using the *in vivo* DNA assembly method in the *E. coli* MC1061 strain [4].	ST8:<br>ATGTATATCTCCTTCTTAAAGTTAAAC<br><br>MM102:<br>AAGCTTACGGTCTCCAGC

pBAD:mScarlet-$I3_{ASV}$	This plasmid was constructed in two steps. (1) pBAD:mScarlet-I3 was amplified using the MM162 / MM174 primers, which add the ASV degradation tag (2) PCR products were *DpnI* treated and gel-purified. Fragments were then ligated using the *in vivo* DNA assembly method in the *E. coli* MC1061 strain [4].	MM162:<br>AAGCTTACGGTCTCCAGCTTGGC<br><br>MM174:<br>GCTGGAGACCGTAAGCTTTTAAACTGATGCAGCGTAGTTTTCGTCGTTTGCTGCAGGCCTGGAGCCACCGGAGCCGCCG
pBAD:GFP_{LVA}<br>pBAD:$mCherry_{LVA}$<br>pBAD:$mCerulean_{LVA}$<br>pBAD:mScarlet-$I3_{LVA}$	These plasmids were constructed in three steps. (1) For each fluorescent protein, the respective plasmid containing the fluorescent protein with ASV-tag (listed above) was amplified to exchange the ASV-tag for the LVA-tag using the MM102 / MM129 primers. (2) PCR products were *DpnI* treated and gel-purified. Fragments were then ligated using the *in vivo* DNA assembly method in the *E. coli* MC1061 strain [4].	MM102:<br>AAGCTTACGGTCTCCAGC<br><br>MM129:<br>CCGTAAGCTTTTAAGCTACTAAAGCGTAGTTTTCGTCGTTTGC

References

(1) Zutz, A., Hamborg, L., Pedersen, L. E., Kassem, M. M., Papaleo, E., Koza, A., Herrgård, M. J., Jensen, S. I., Teilum, K., Lindorff-Larsen, K., and Nielsen, A. T. (2021) A dual-reporter system for investigating and optimizing protein translation and folding in E. coli. *Nat Commun 12*, 6093.
(2) Kraft, M., Knüpfer, U., Wenderoth, R., Pietschmann, P., Hock, B., and Horn, U. (2007) An online monitoring system based on a synthetic sigma32-dependent tandem promoter for visualization of insoluble proteins in the cytoplasm of Escherichia coli. *Appl Microbiol Biotechnol 75*, 397–406.
(3) Englund, E., Schmidt, M., Nava, A. A., Klass, S., Keiser, L., Dan, Q., Katz, L., Yuzawa, S., and Keasling, J. D. (2023) Biosensor Guided Polyketide Synthases Engineering for Optimization of Domain Exchange Boundaries. *Nat Commun 14*, 4871.
(4) Watson, J. F., and García-Nafría, J. (2019) In vivo DNA assembly using common laboratory bacteria: A re-emerging tool to simplify molecular cloning. *J Biol Chem 294*, 15271–15281.
(5) Cumming, A. J., Khananisho, D., Balka, M., Liljestrand, N., and Daley, D. O. (2024) Biosensor that Detects Stress Caused by Periplasmic Proteins. *ACS Synth. Biol. 13*, 1477–1491.
(6) Shilling, P. J., Mirzadeh, K., Cumming, A. J., Widesheim, M., Köck, Z., and Daley, D. O. (2020) Improved designs for pET expression plasmids increase protein production yield in Escherichia coli. *Commun Biol 3*, 214.
(7) Shilling, P. J., Khananisho, D., Cumming, A. J., Söderström, B., and Daley, D. O. (2022) Signal amplification of *araC pBAD* using a standardized translation initiation region. *Synthetic Biology 7*, ysac009.
(8) Söderström, B., Skoog, K., Blom, H., Weiss, D. S., Von Heijne, G., and Daley, D. O. (2014) Disassembly of the divisome in *E scherichia coli* : evidence that FtsZ dissociates before compartmentalization. *Molecular Microbiology 92*, 1–9.
(9) Paetzel, M. (2014) Structure and mechanism of Escherichia coli type I signal peptidase. *Biochimica et Biophysica Acta (BBA) - Molecular Cell Research 1843*, 1497–1508.
(10) Price, N. L., and Raivio, T. L. (2009) Characterization of the Cpx regulon in Escherichia coli strain MC4100. *J Bacteriol 191*, 1798–1815.
(11) Betton, J.-M., Sassoon, N., Hofnung, M., and Laurent, M. (1998) Degradation versus Aggregation of Misfolded Maltose-binding Protein in the Periplasm of Escherichia coli. *Journal of Biological Chemistry 273*, 8897–8902.
